# Supplementary material for: Transactive Response DNA-Binding Protein (TARDBP/TDP-43) Regulates Cell Permissivity to HIV-1 Infection by Acting on HDAC6
Source: Int J Mol Sci. 2022 May 31;23(11):6180. doi: 10.3390/ijms23116180 (PMC9181786; doi:10.3390/ijms23116180)

Figure S1. Western-blot replicates associated with Figure 1A Cabrera-Rodríguez R., *et al.*

Replicate 1 as figure format

A

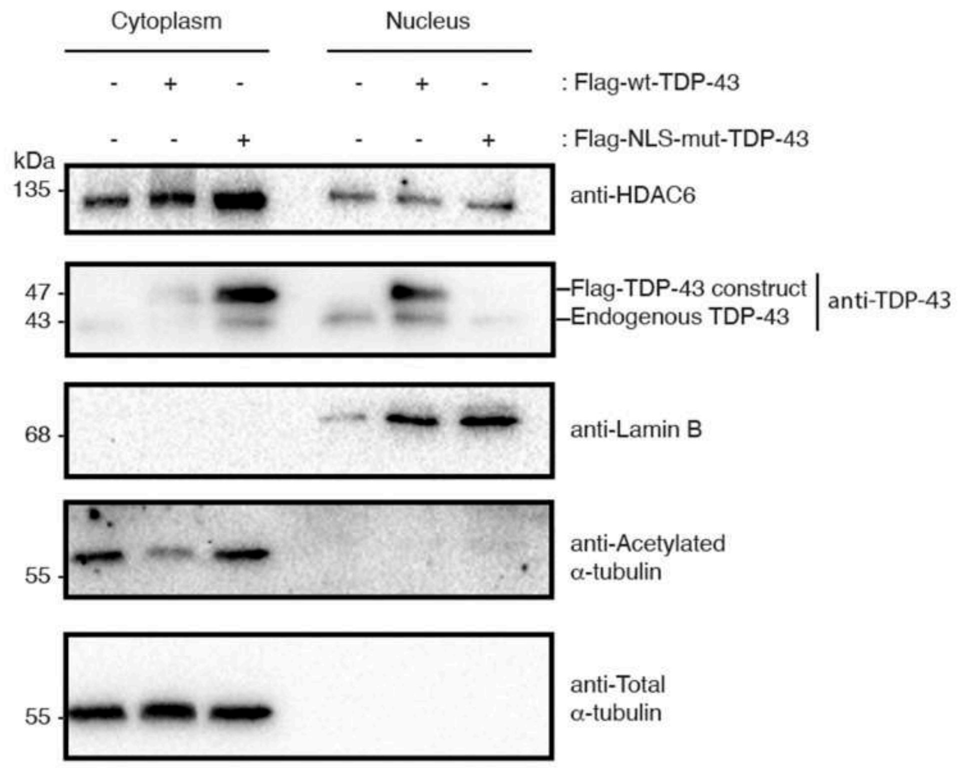

Replicate 2 as figure format

A

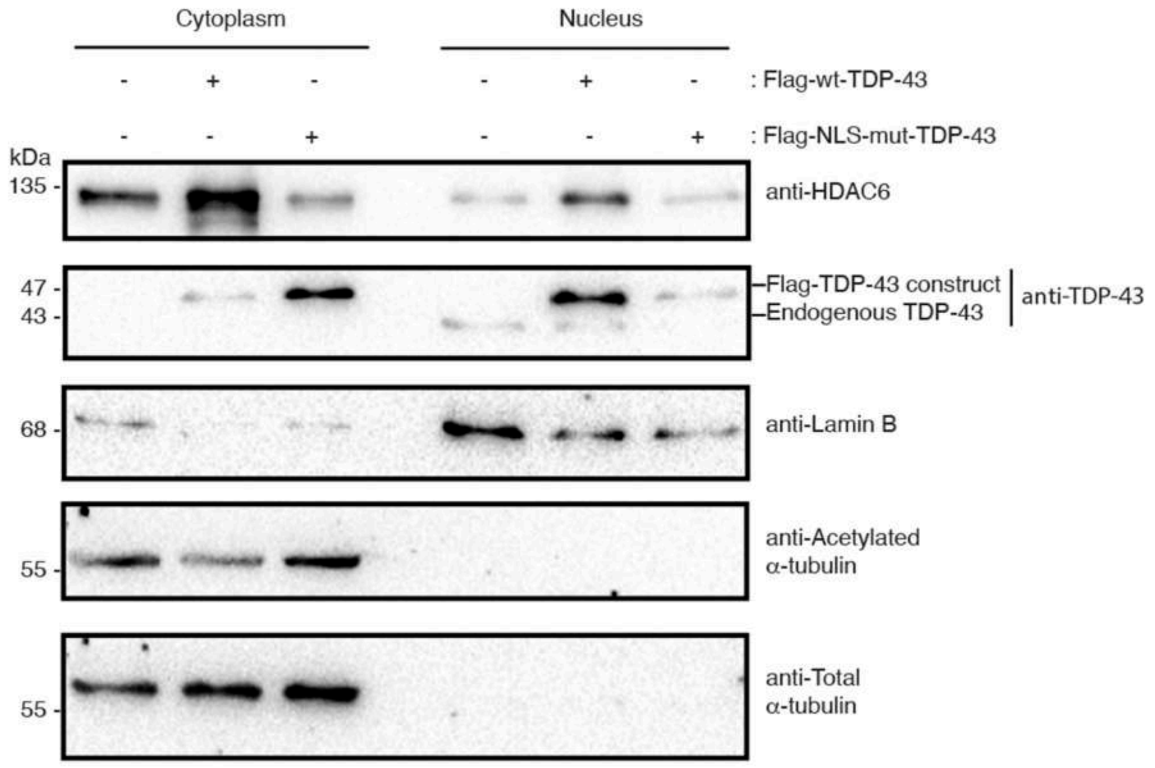

Replicate 3 as figure format

A

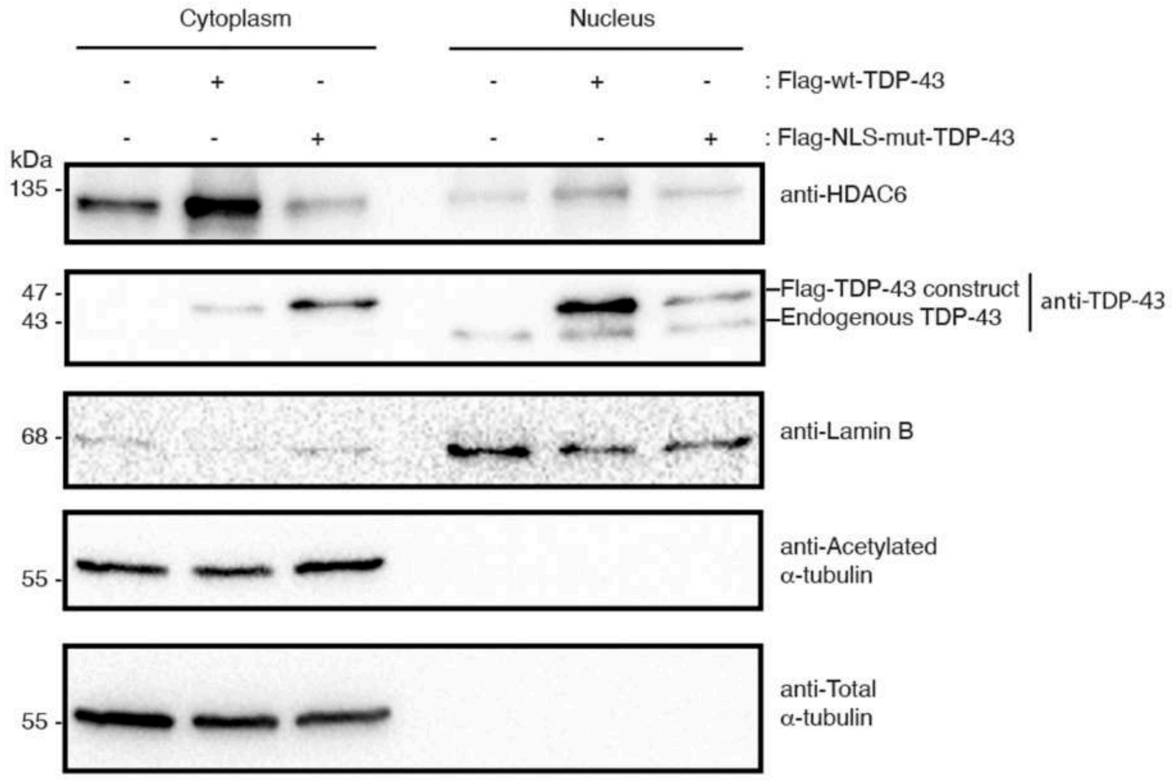

Figure S1. Replicate 1 HDAC6 complete gel   Western-blot associated with Figure 1A
Cabrera-Rodríguez, R., *et al.*

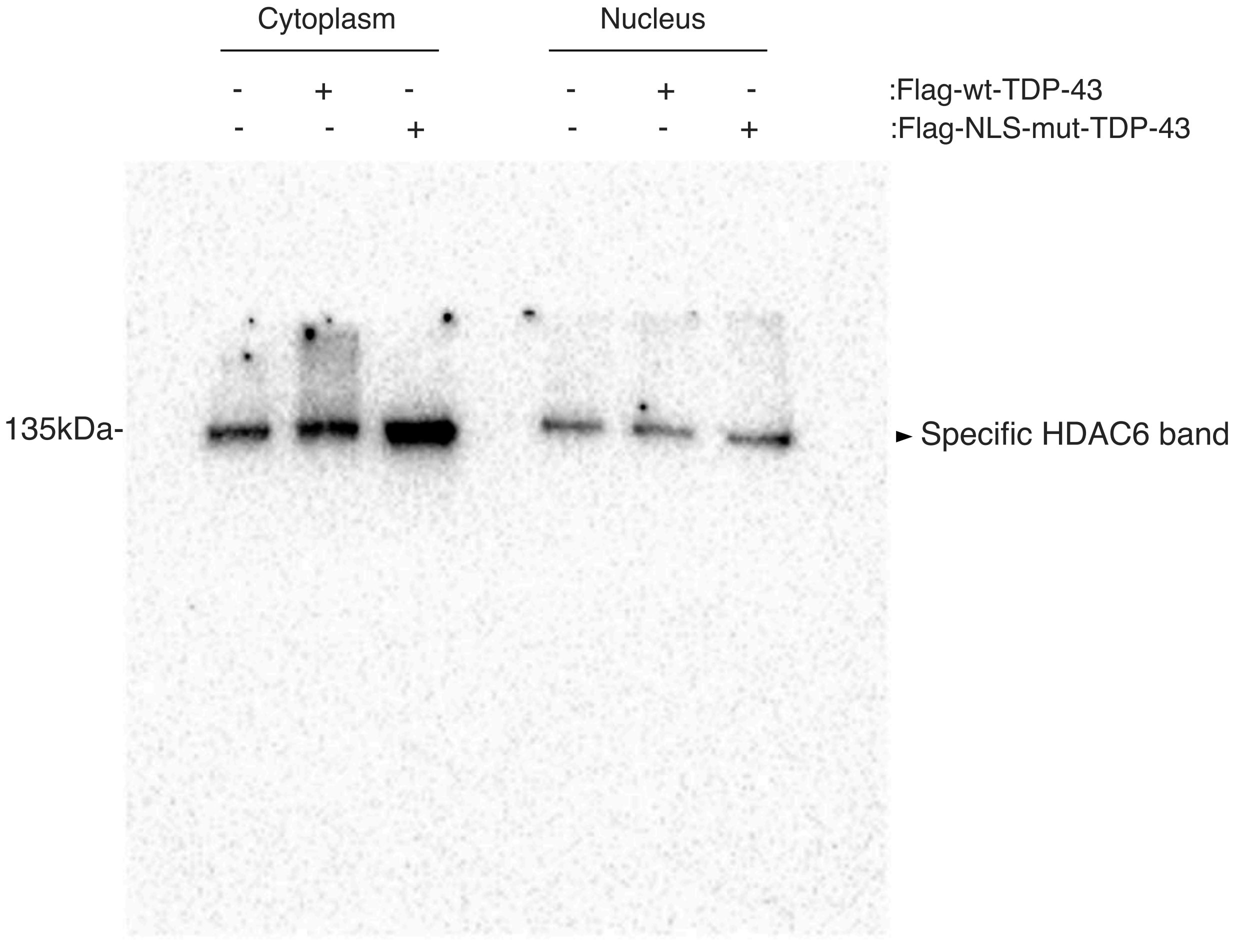

Figure S1. Replicate 1 TDP-43 complete gel Western-blot associated with Figure 1A

Cabrera-Rodríguez, R., *et al.*

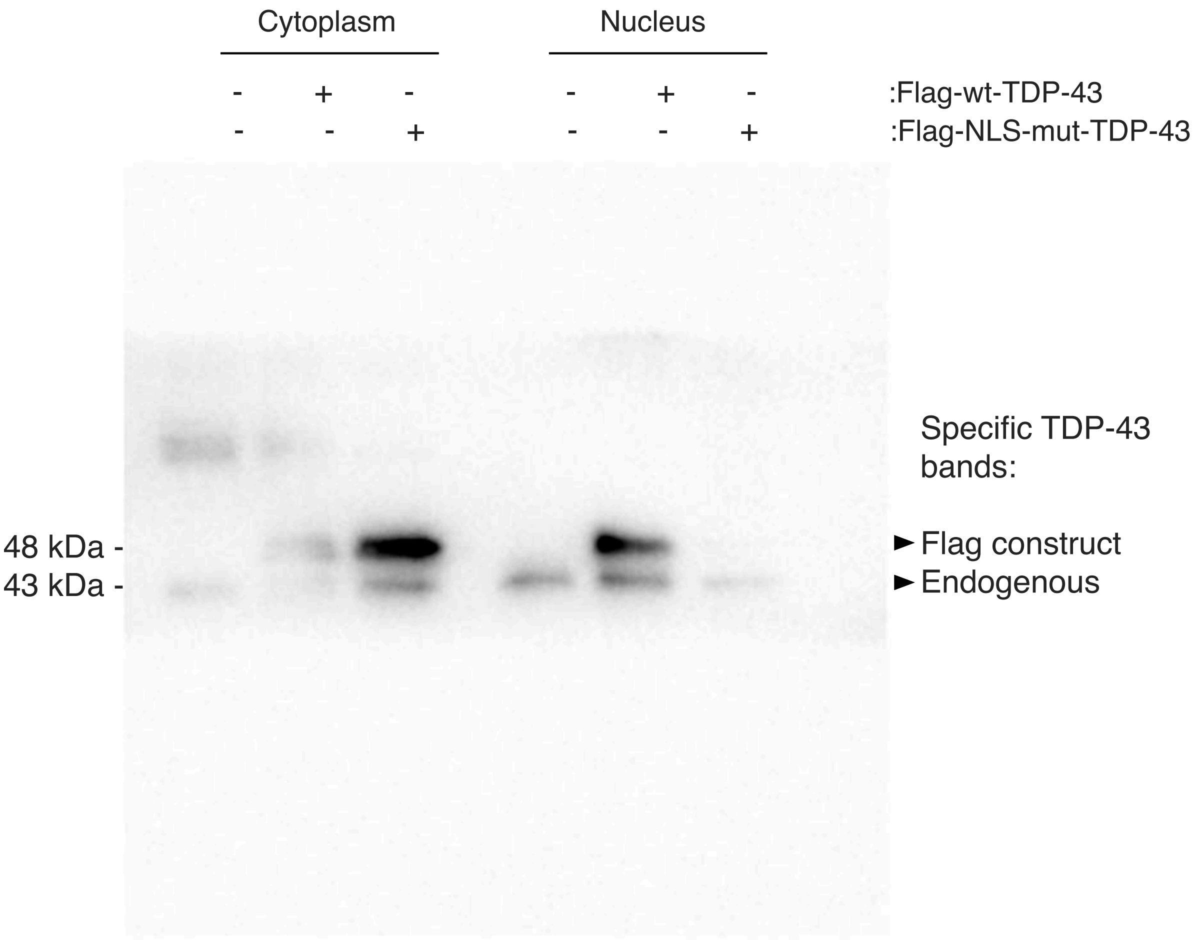

Figure S1. Replicate 1 Lamin B complete gel   Western-blot associated with Figure 1A
Cabrera-Rodríguez, R., *et al.*

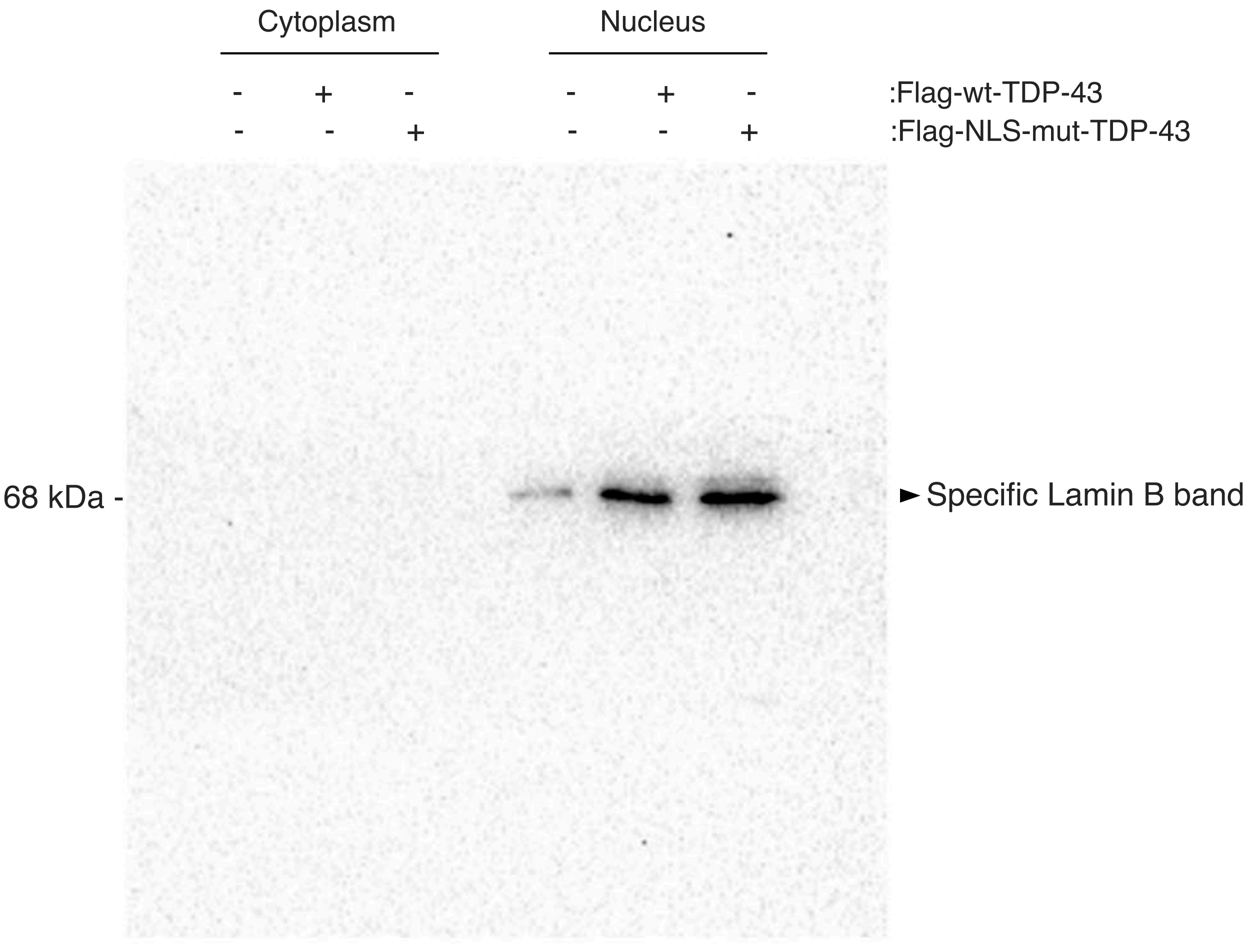

Figure S1. Replicate 1 Acetylated α-tubulin complete gel   Western-blot associated with Figure 1A

Cabrera-Rodríguez, R., *et al.*

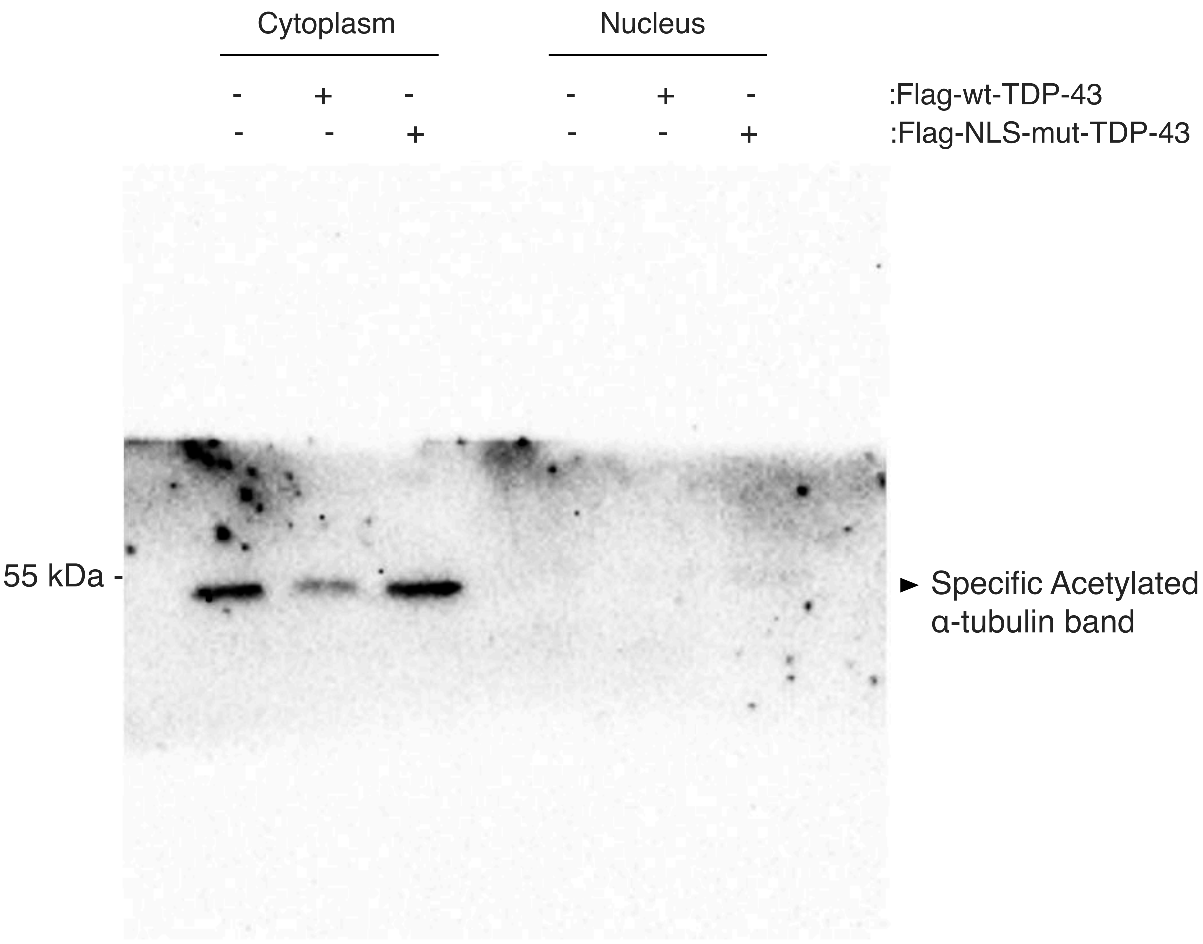

Figure S1. Replicate 1 Total α-tubulin complete gel   Western-blot associated with Figure 1A
Cabrera-Rodríguez, R., *et al.*

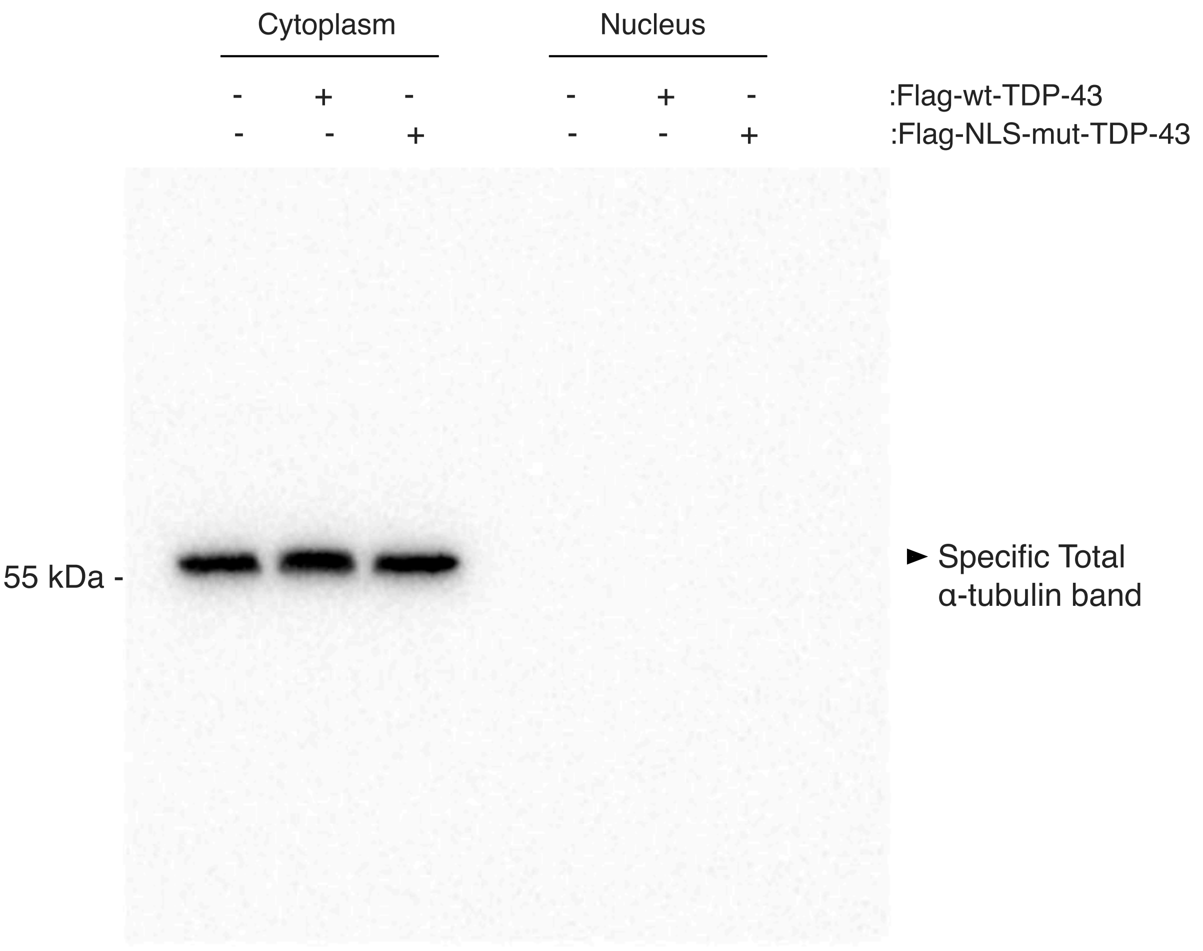

Figure S1. Replicate 2 HDAC6 complete gel   Western-blot associated with Figure 1A
Cabrera-Rodríguez, R., *et al.*.

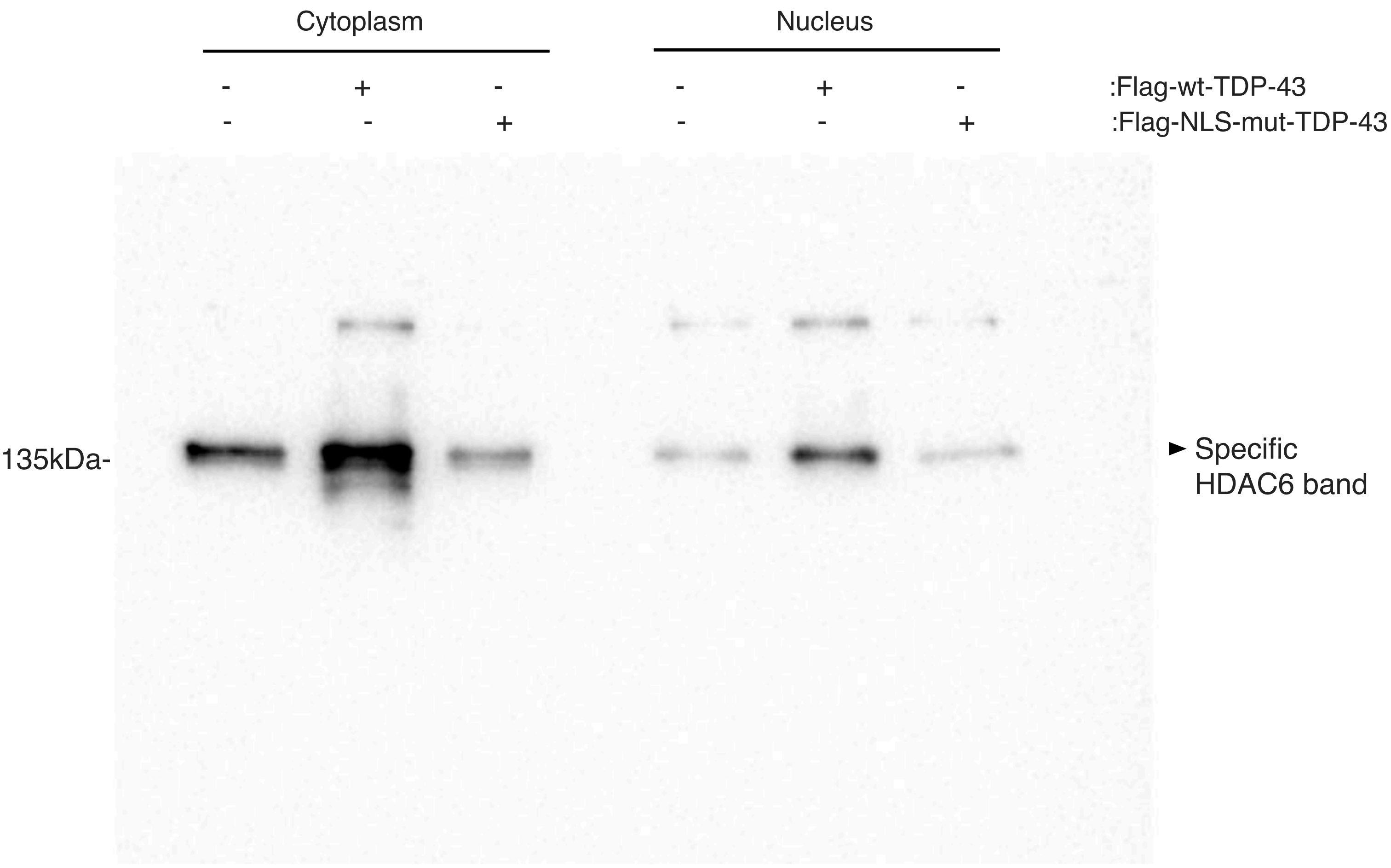

Figure S1. Replicate 2 TDP-43 complete gel Western-blot associated with Figure 1A
Cabrera-Rodríguez, R., *et al.*.

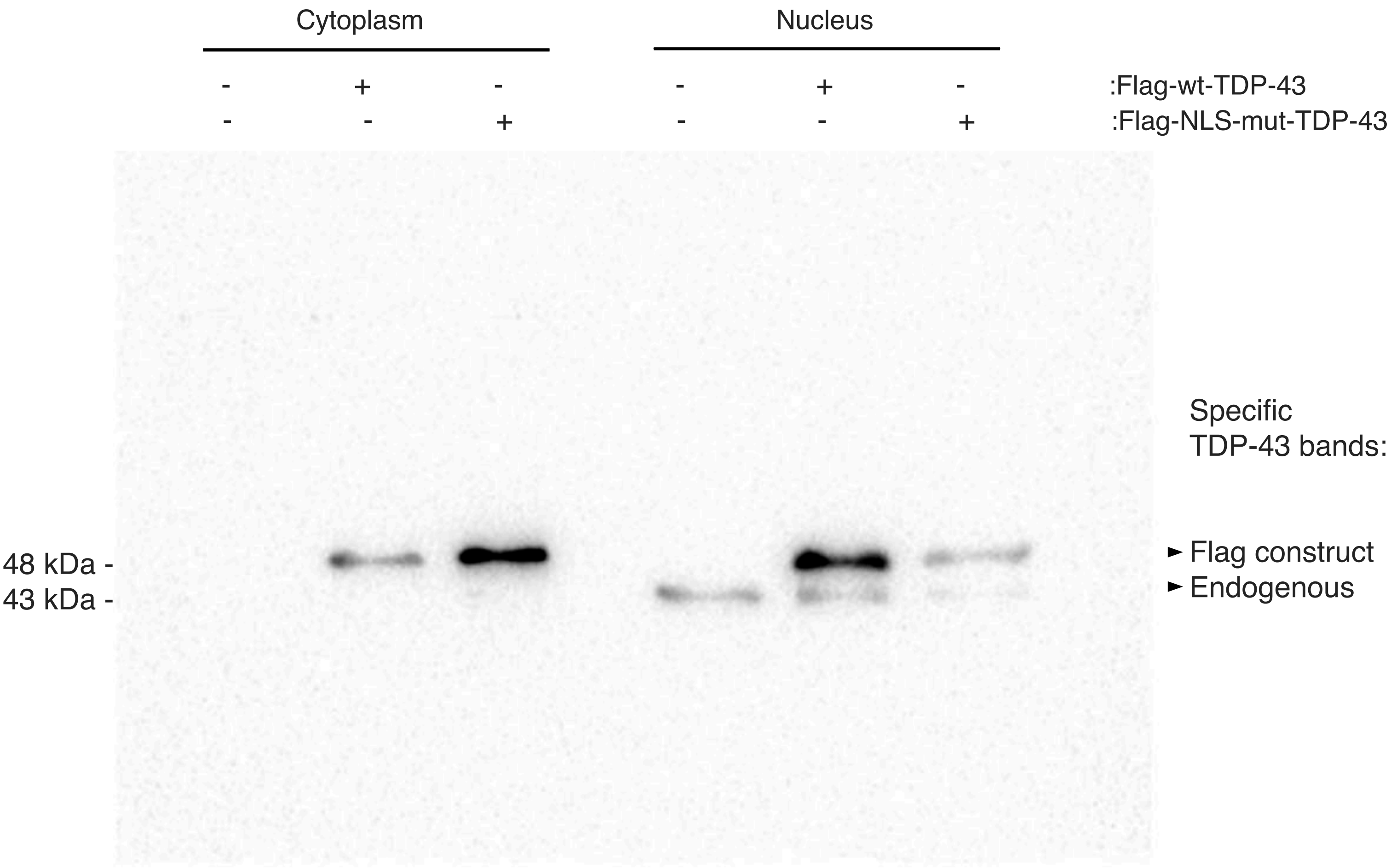

Figure S1. Replicate 2 Lamin B complete gel Western-blot associated with Figure 1A

Cabrera-Rodríguez, R., *et al.*.

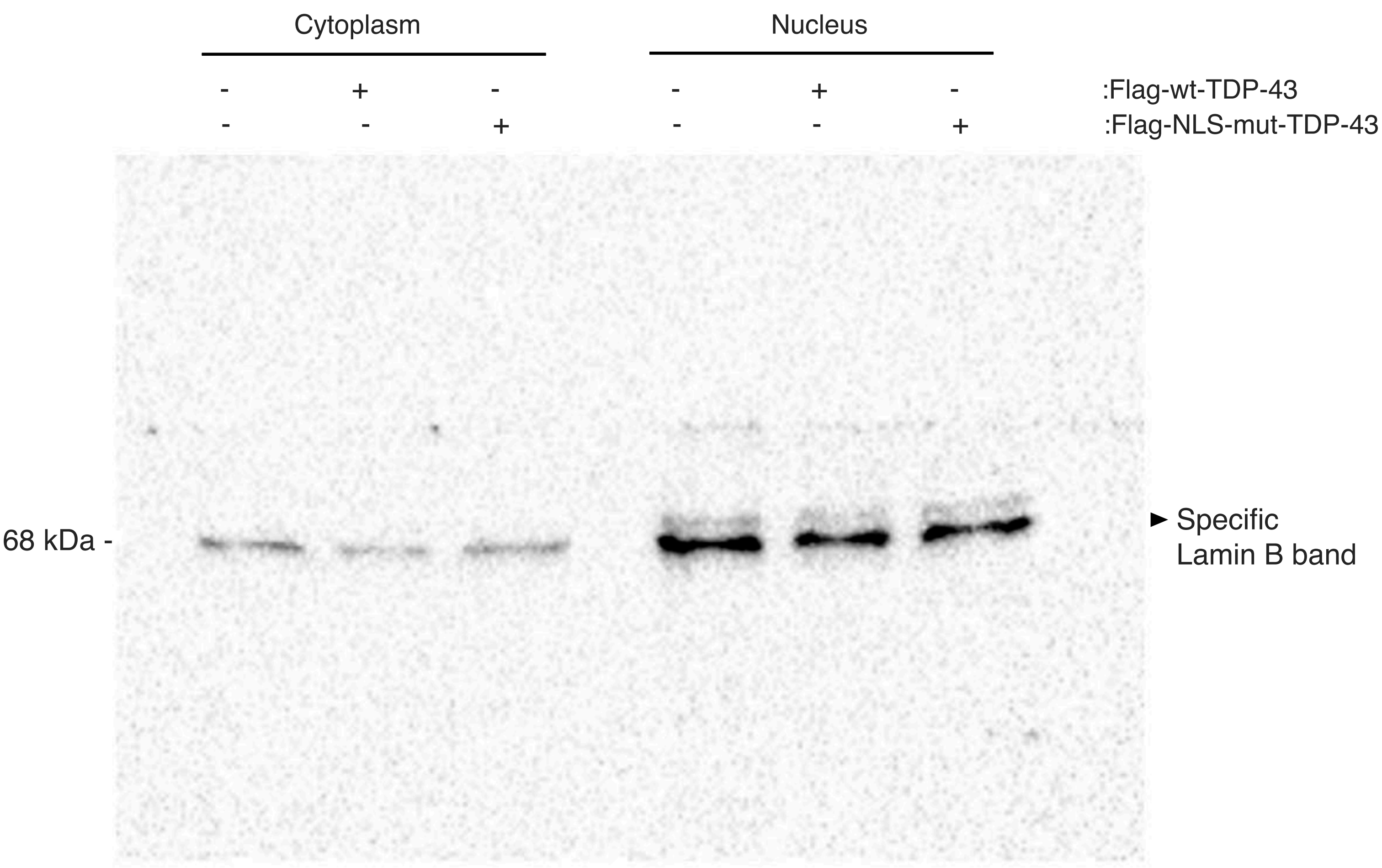

Figure S1. Replicate 2 Acetylated α-tubulin complete gel   Western-blot associated with Figure 1A
Cabrera-Rodríguez, R., *et al.*

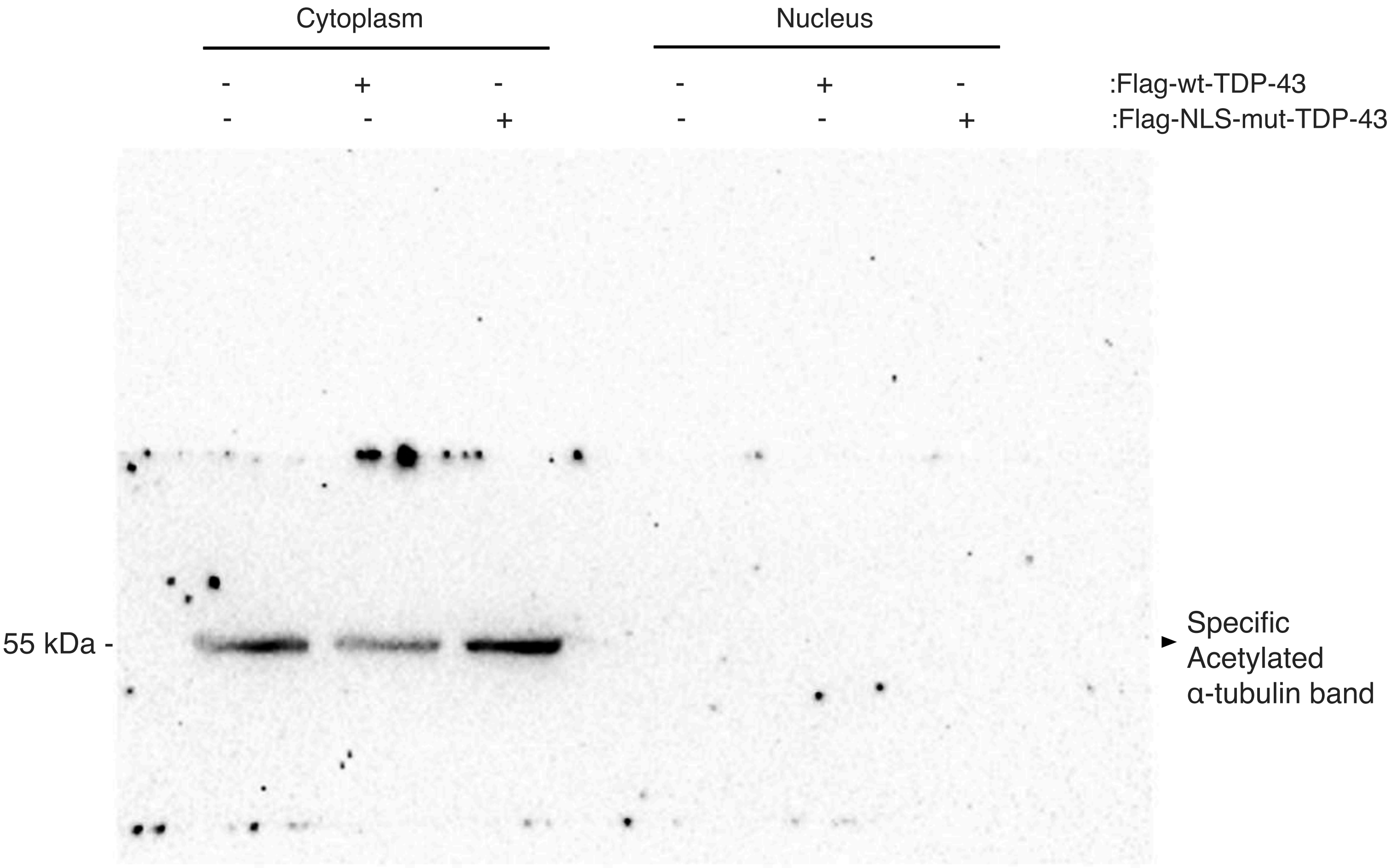

Figure S1. Replicate 2 Total α-tubulin complete gel Western-blot associated with Figure 1A

Cabrera-Rodríguez, R., *et al.*.

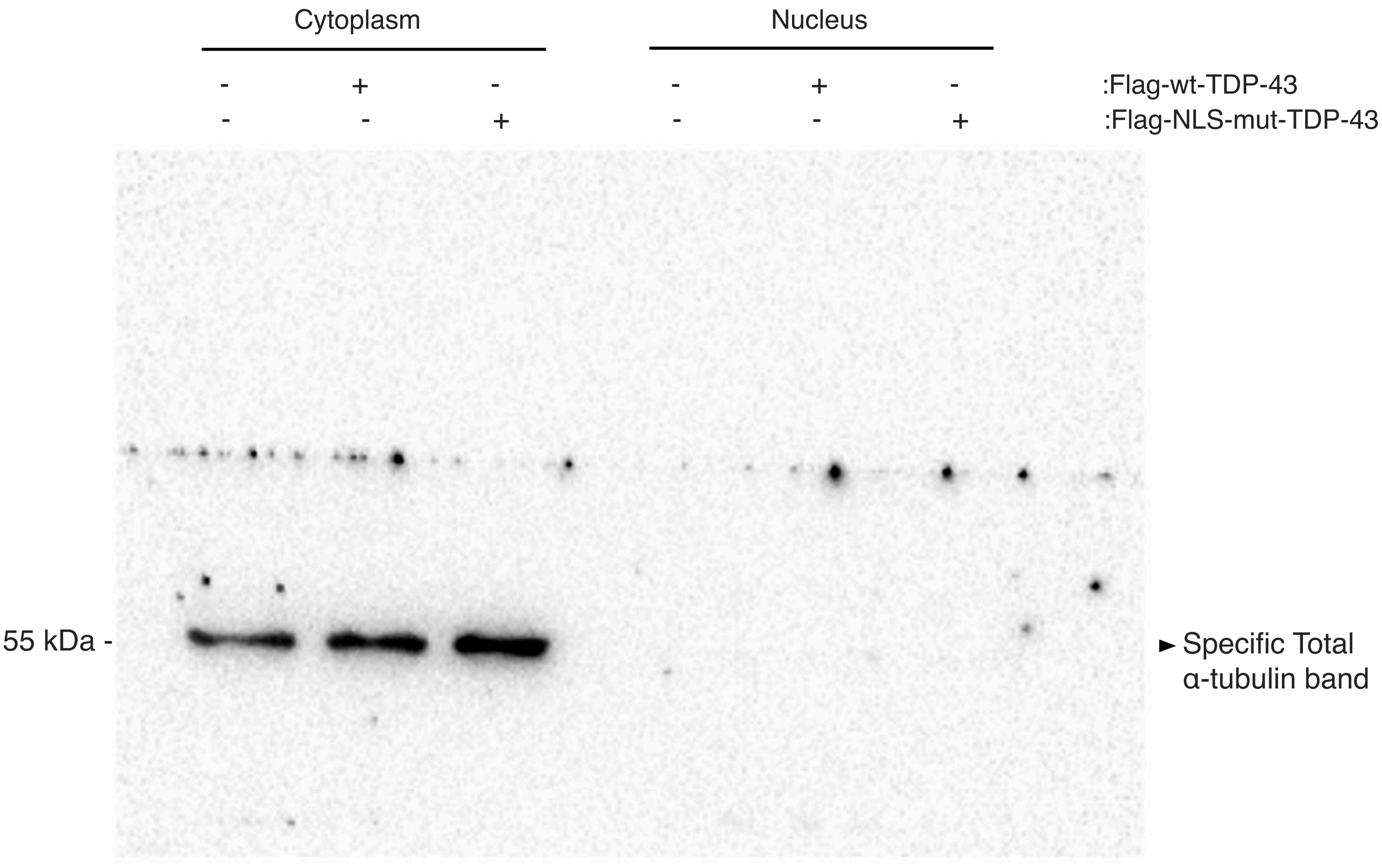

Figure S1. Replicate 3 HDAC6 complete gel Western-blot associated with Figure 1A
Cabrera-Rodríguez, R., *et al.*.

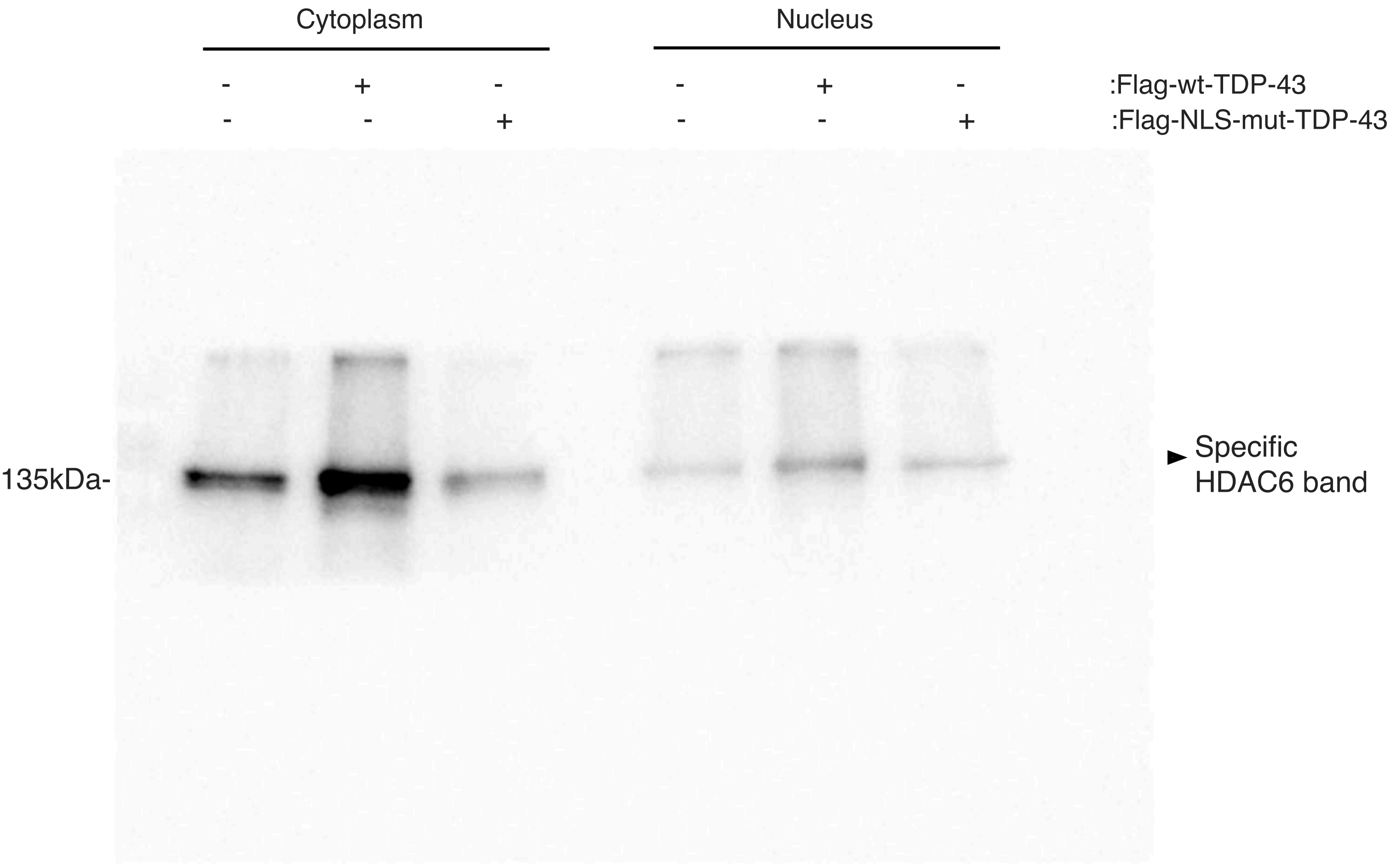

Figure S1. Replicate 3 TDP-43 complete gel Western-blot associated with Figure 1A
Cabrera-Rodríguez, R., *et al.*

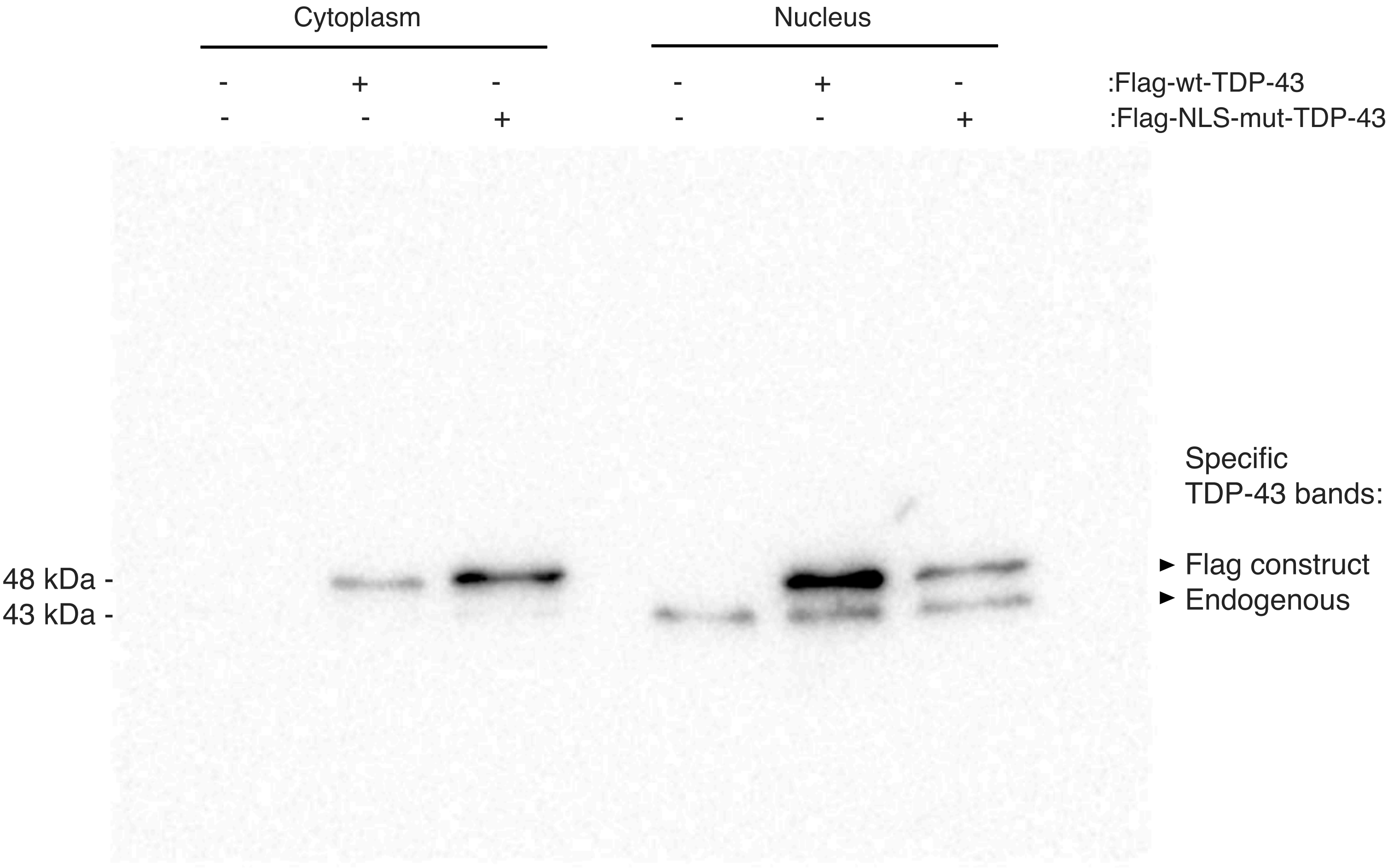

Figure S1. Replicate 3 Lamin B complete gel   Western-blot associated with Figure 1A
Cabrera-Rodríguez, R., *et al.*.

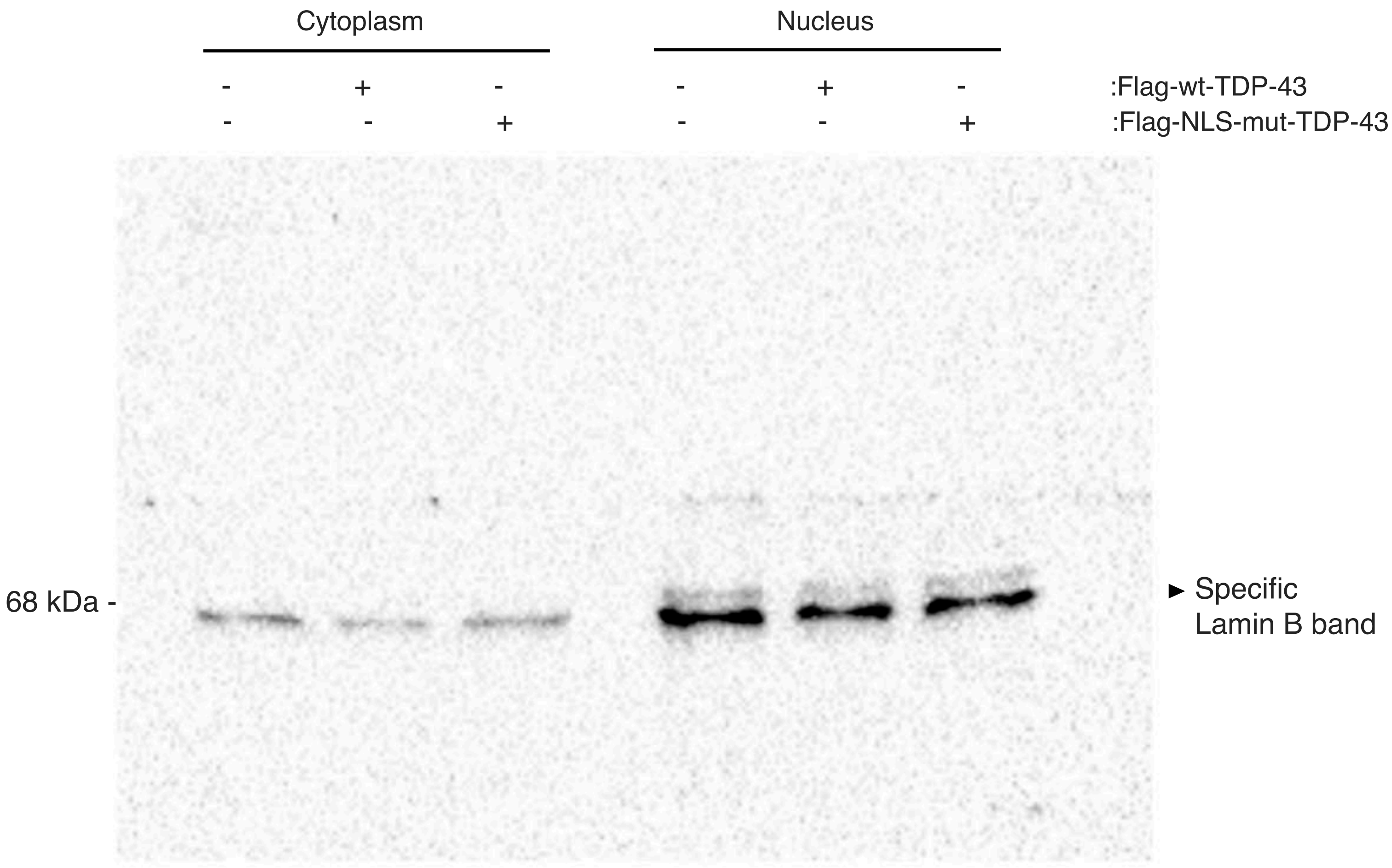

Figure S1. Replicate 3 Acetylated α-tubulin complete gel Western-blot associated with Figure 1A
Cabrera-Rodríguez, R., *et al.*.

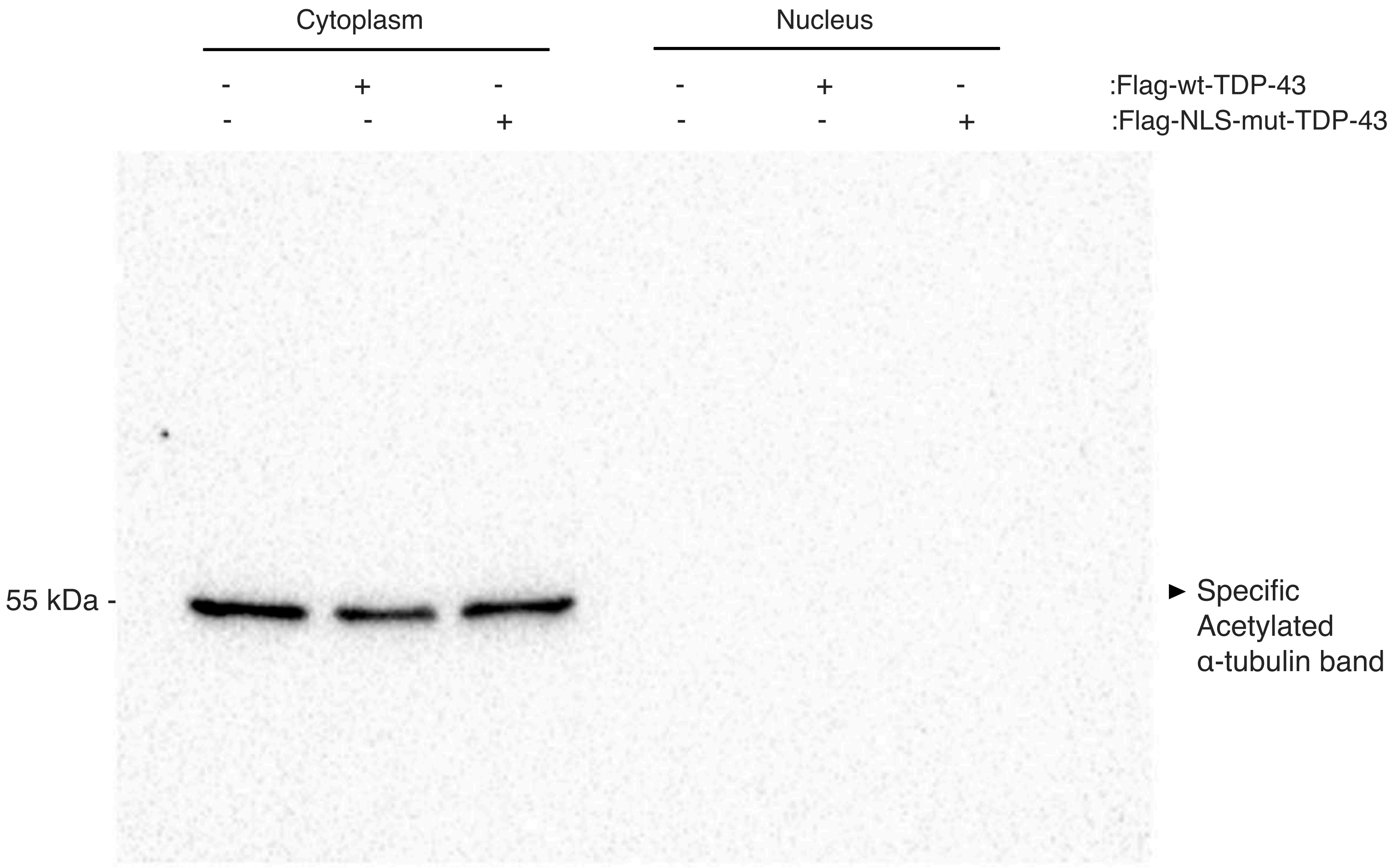

Figure S1. Replicate 3 Total α-tubulin complete gel Western-blot associated with Figure 1A
Cabrera-Rodríguez, R., *et al.*.

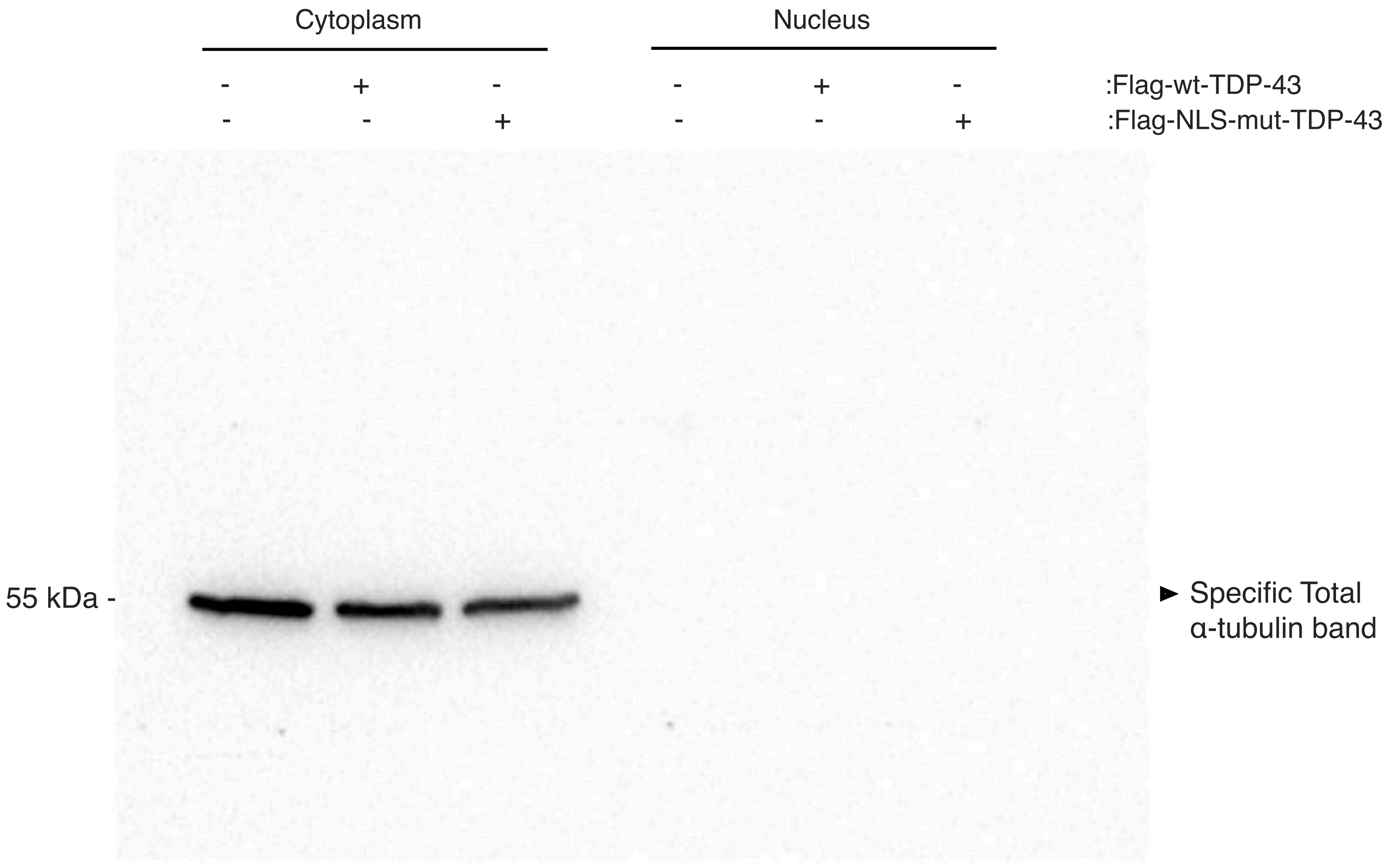

Figure S2. Western-blot replicates associated with Figure 3A Cabrera-Rodríguez R., *et al.*

Replicate 1 as figure format

A

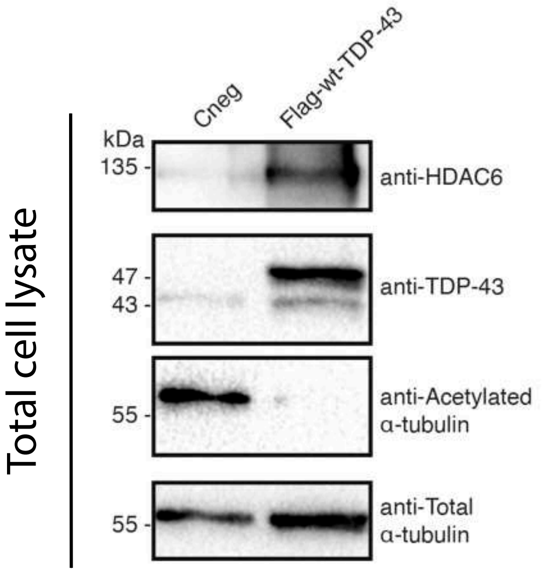

Replicate 1 as figure format

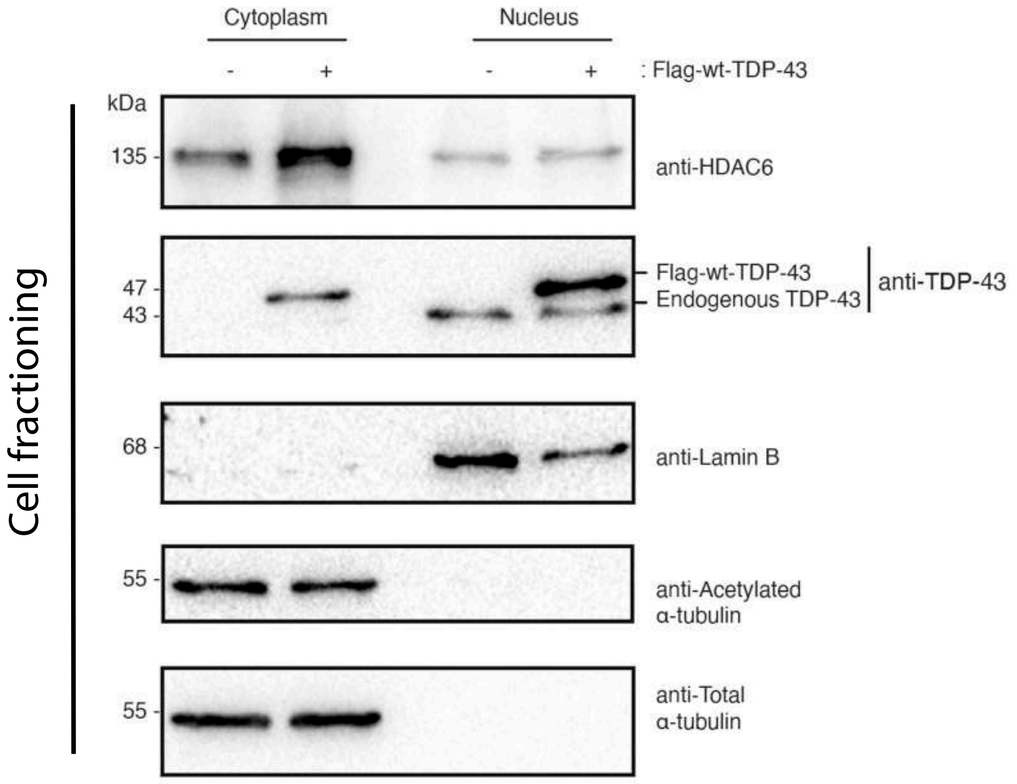

Replicate 2 as figure format

A

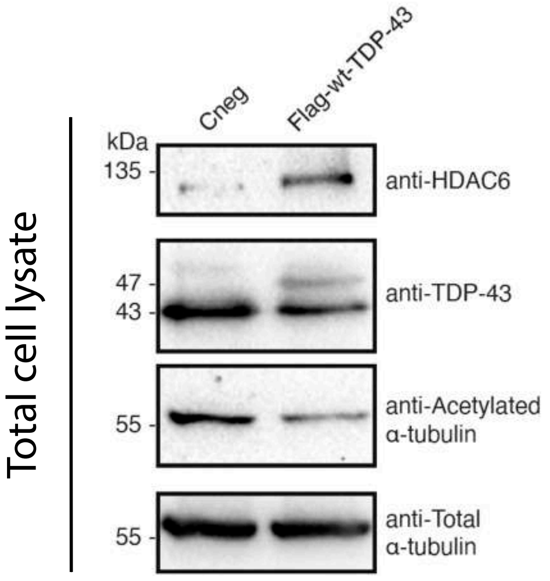

Replicate 2 as figure format

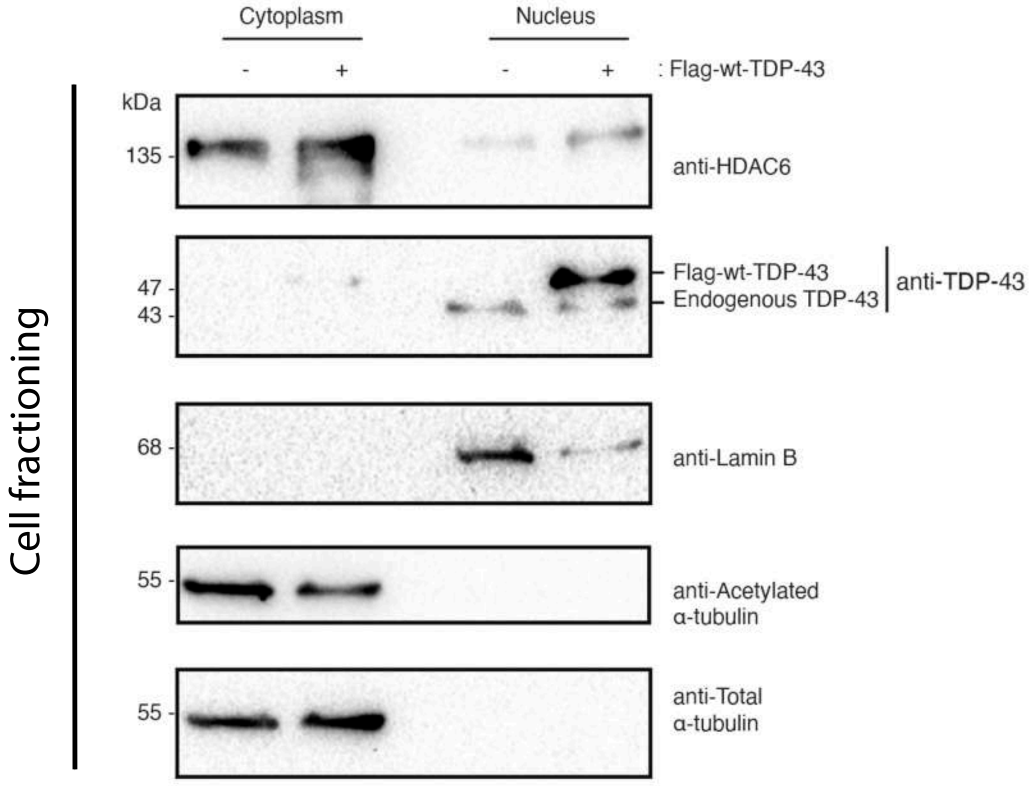

Replicate 3 as figure format

A

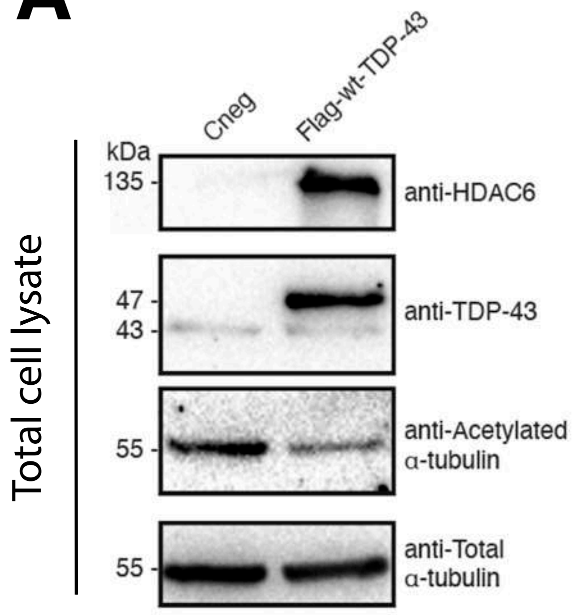

Replicate 3 as figure format

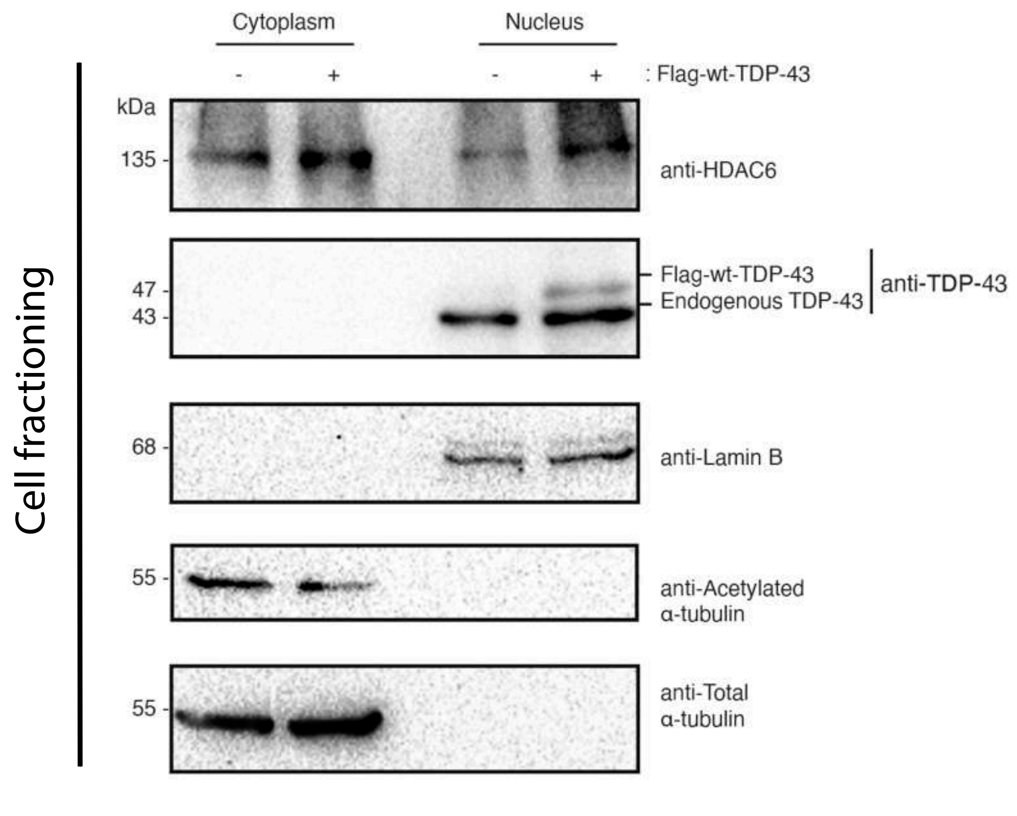

Figure S2. Replicate 1 HDAC6 complete gel Western-blot associated with Figure 3A
Cabrera-Rodríguez, R., *et al.*

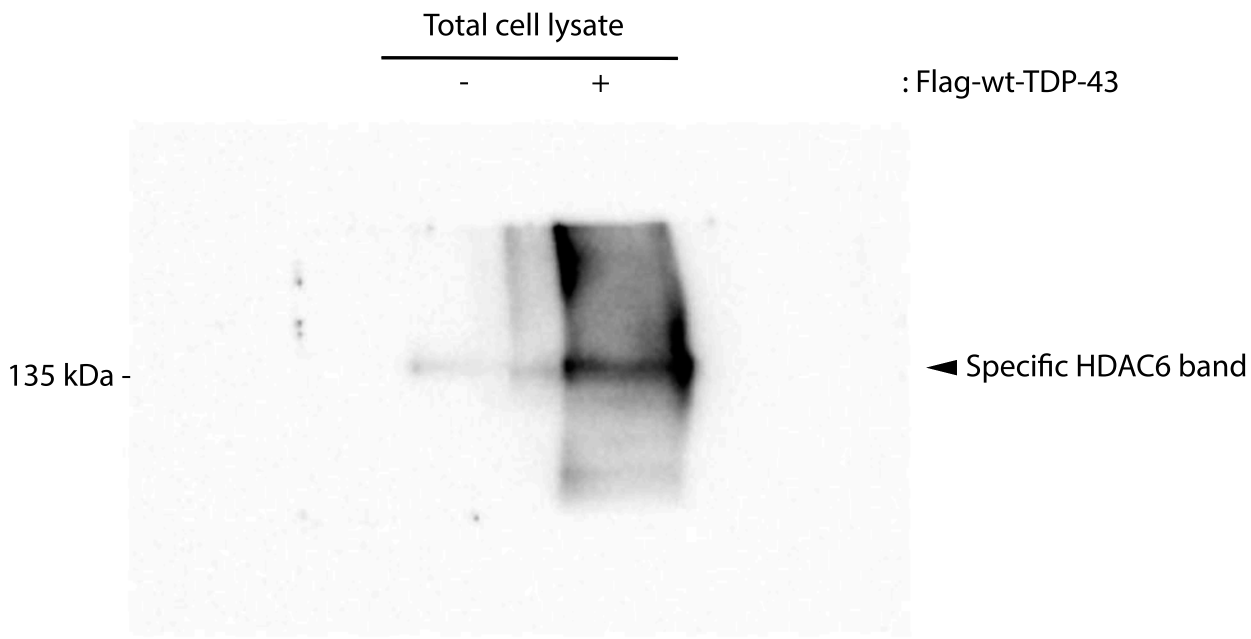

Figure S2. Replicate 1 TDP-43 complete gel Western-blot associated with Figure 3A
Cabrera-Rodríguez, R., *et al.*

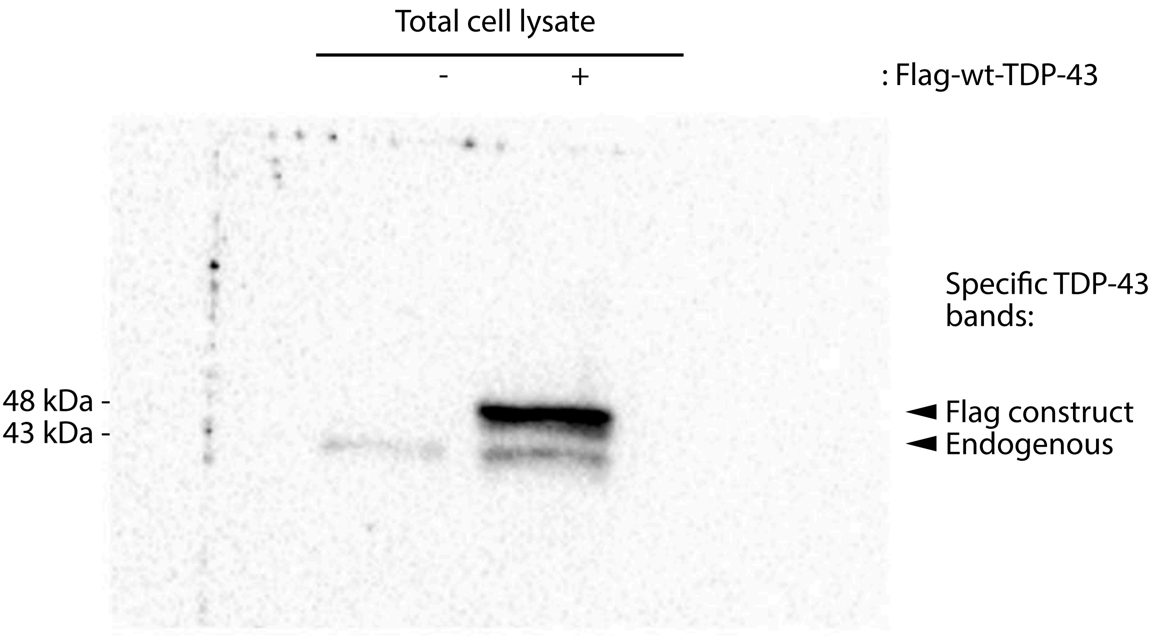

Figure S2. Replicate 1 Acetylated α-tubulin complete gel

Western-blot associated with Figure 3A
Cabrera-Rodríguez, R., *et al.*

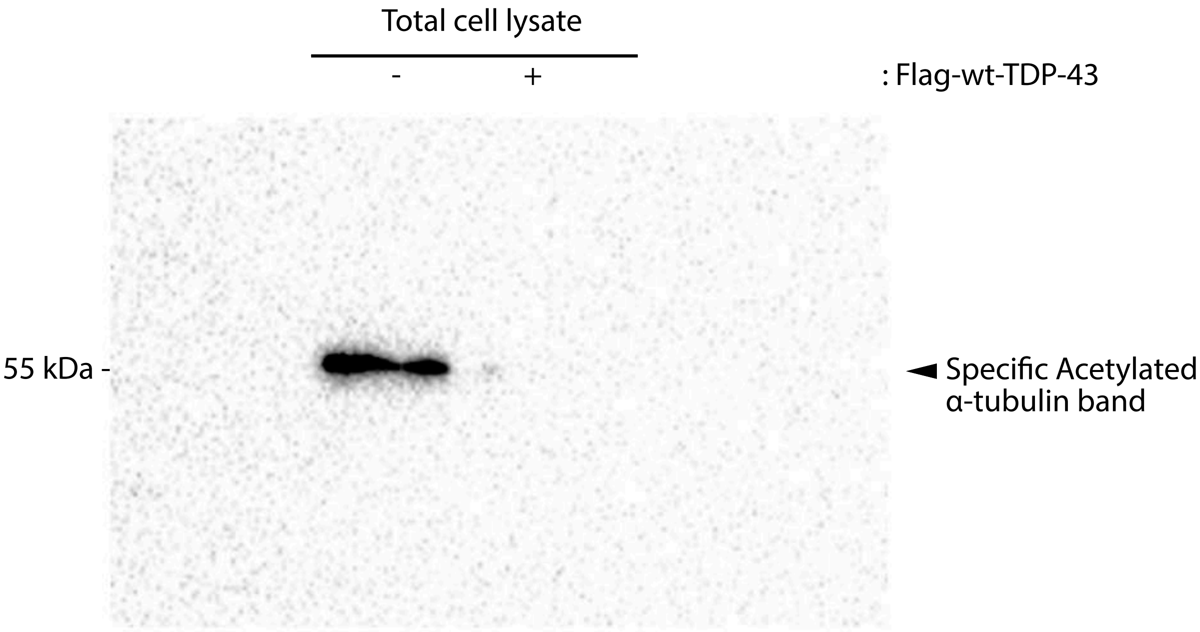

Figure S2. Replicate 1 Total α-tubulin complete gel Western-blot associated with Figure 3A

Cabrera-Rodríguez, R., *et al.*

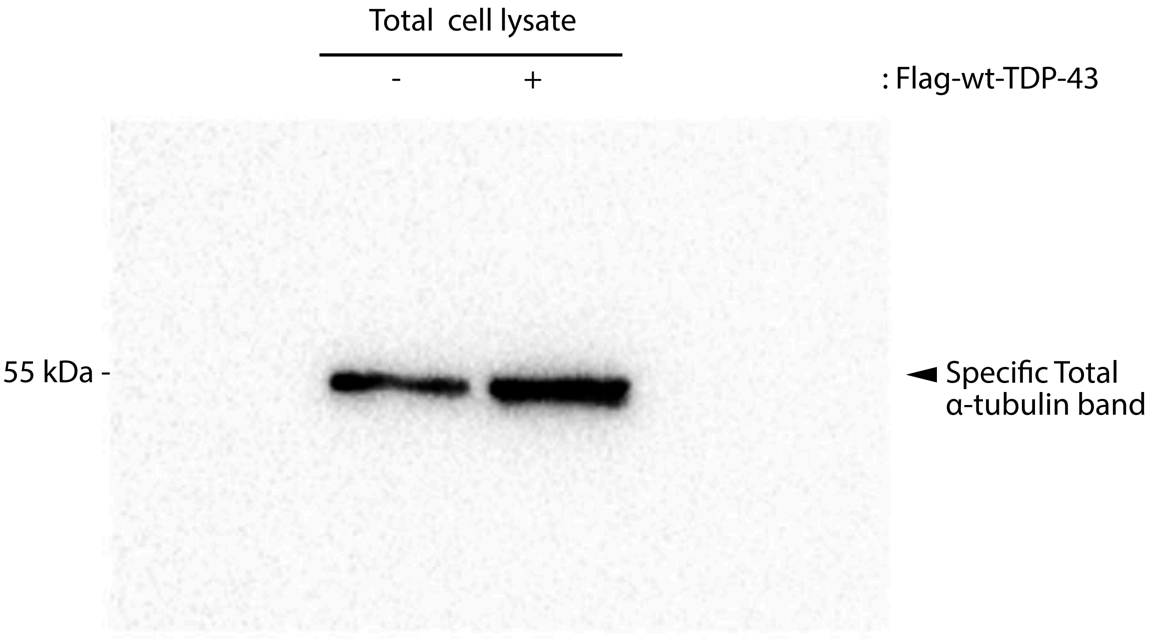

Figure S2. Replicate 1 HDAC6 complete gel Western-blot associated with Figure 3A
Cabrera-Rodríguez, R., *et al.*

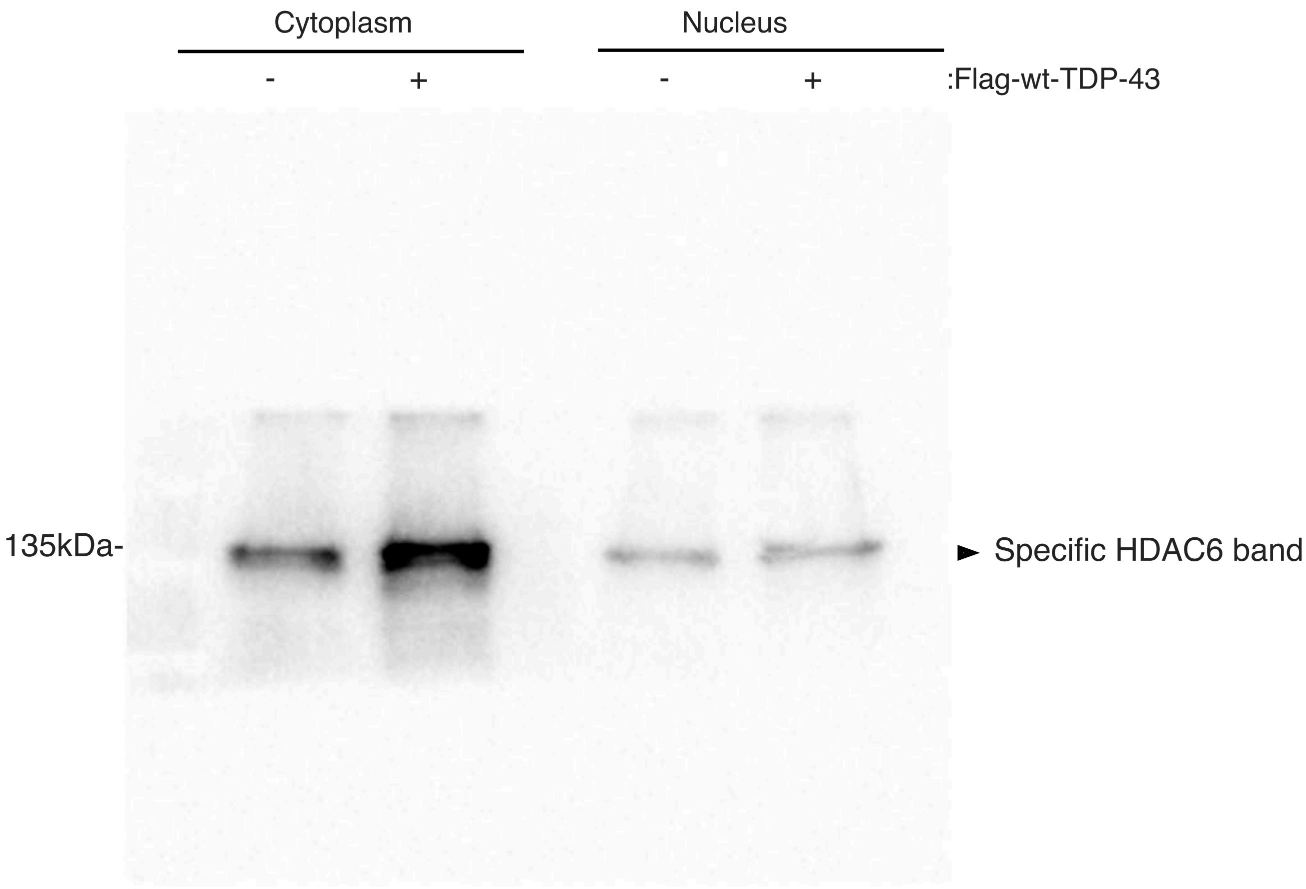

Figure S2. Replicate 1 TDP-43 complete gel Western-blot associated with Figure 3A
Cabrera-Rodríguez, R., *et al.*

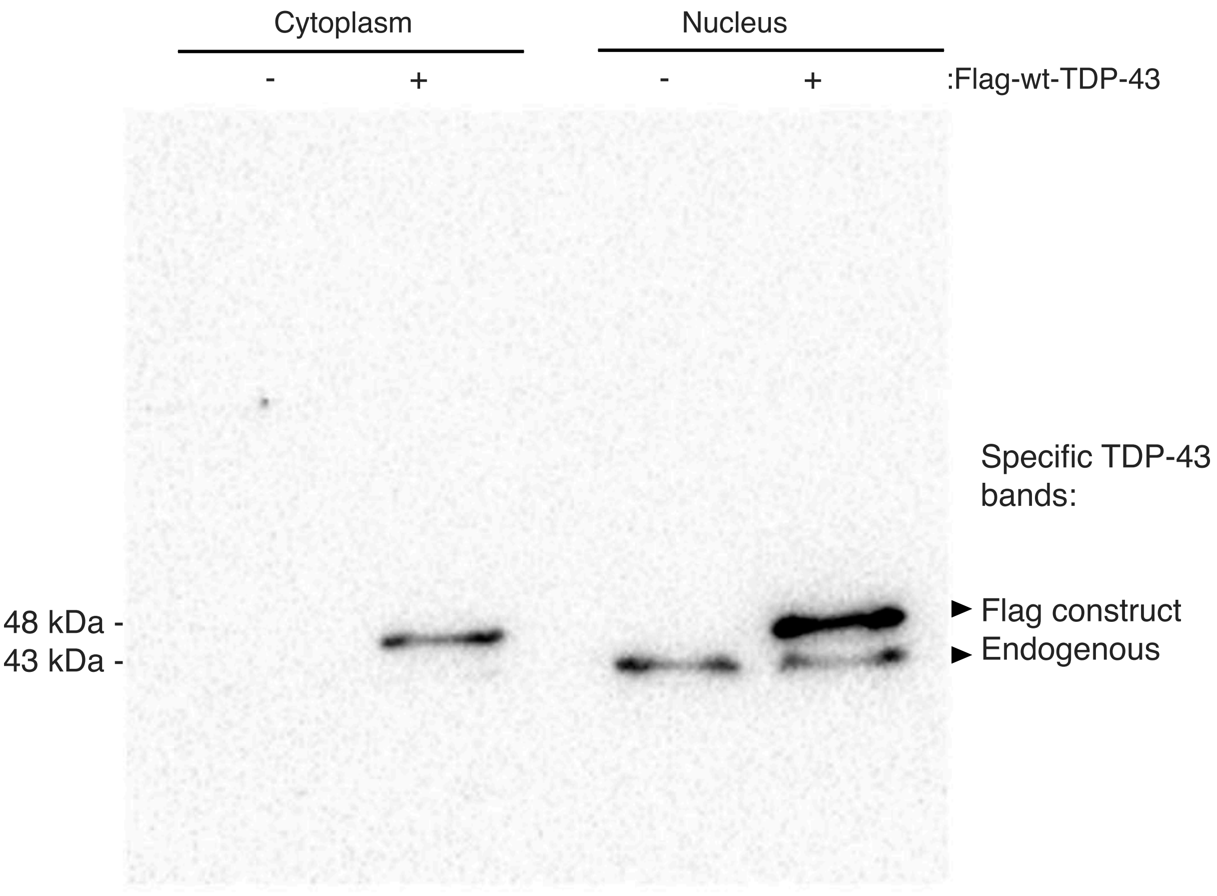

Figure S2. Replicate 1 Lamin B complete gel Western-blot associated with Figure 3A
Cabrera-Rodríguez, R., *et al.*

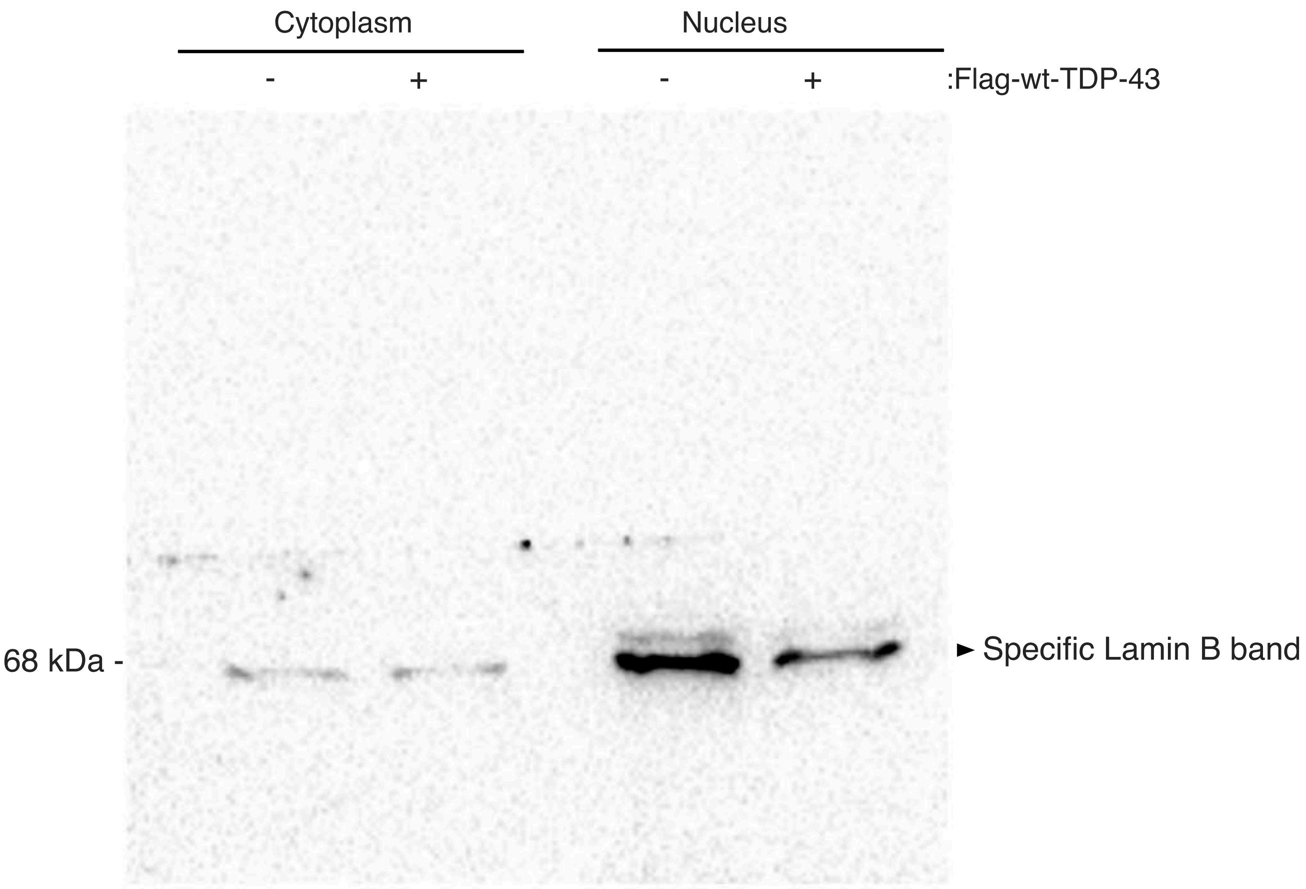

Figure S2. Replicate 1 Acetylated α-tubulin complete gel Western-blot associated with Figure 3A
Cabrera-Rodríguez, R., *et al.*

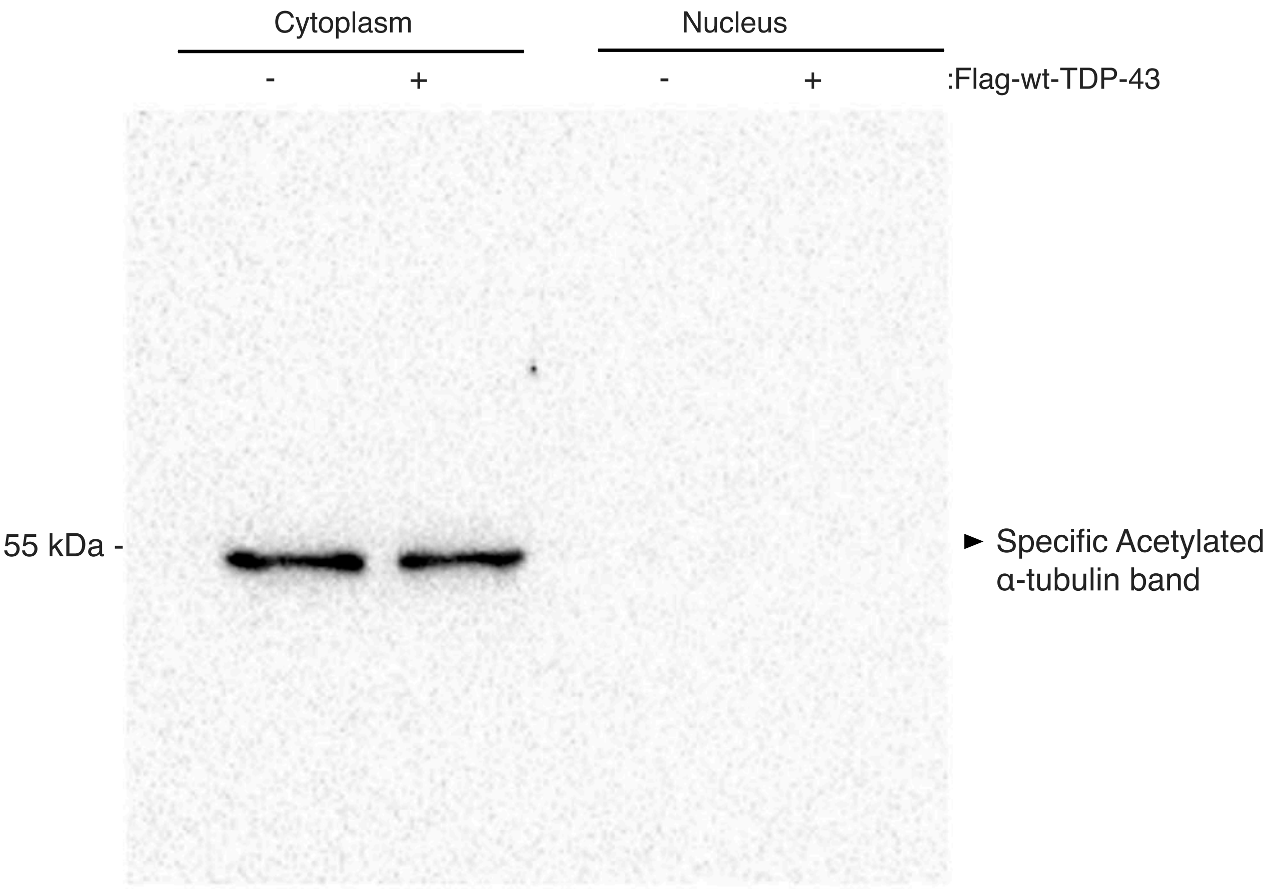

Figure S2. Replicate 1 Total α-tubulin complete gel   Western-blot associated with Figure 3A

Cabrera-Rodríguez, R., *et al.*

Cytoplasm   Nucleus

- + - + :Flag-wt-TDP-43

55 kDa -

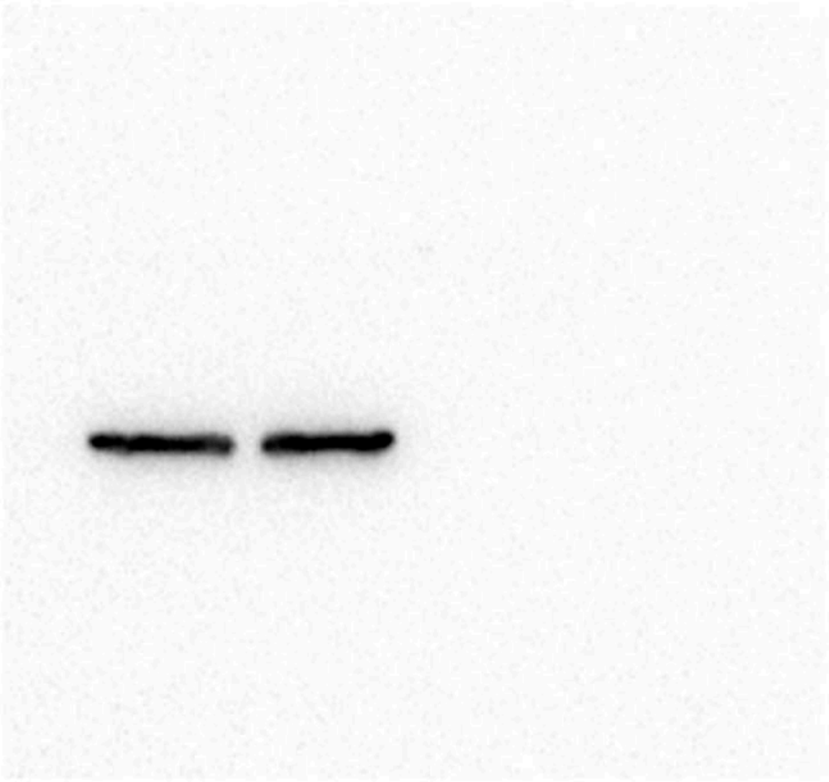

► Specific Total α-tubulin band

Figure S2. Replicate 2 HDAC6 complete gel Western-blot associated with Figure 3A
Cabrera-Rodríguez, R., *et al.*

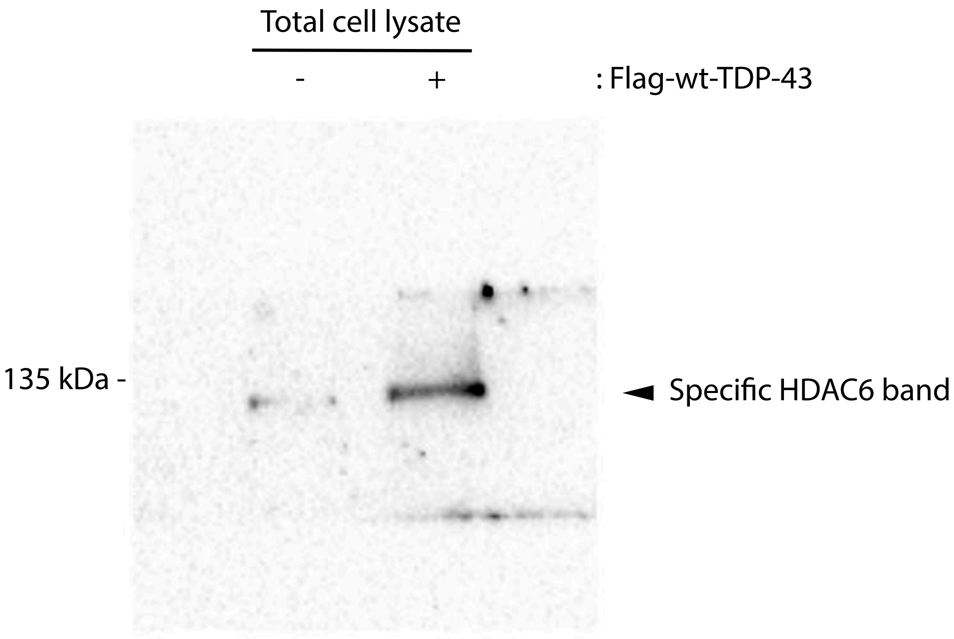

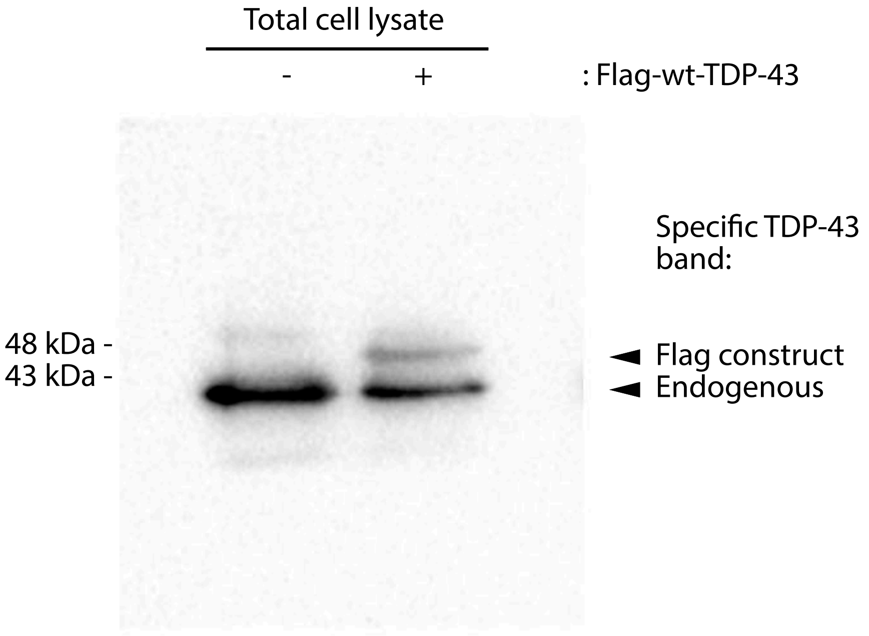
Total cell lysate
-
+
: Flag-wt-TDP-43
48 kDa -
43 kDa -
Specific TDP-43 band:
Flag construct
Endogenous

Figure S2. Replicate 2 Acetylated α-tubulin complete gel Western-blot associated with Figure 3A
Cabrera-Rodríguez, R., *et al.*

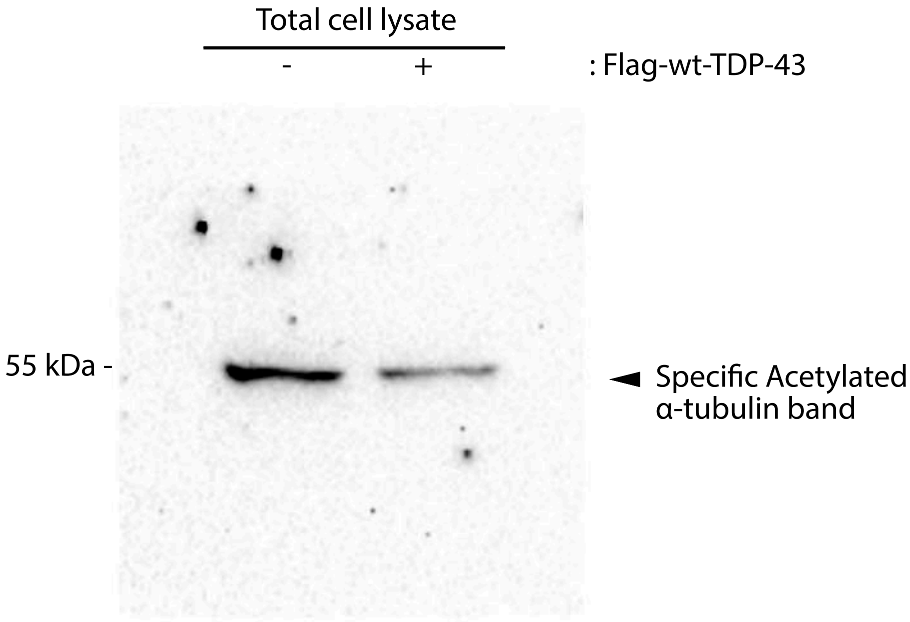

Figure S2. Replicate 2 Total α-tubulin complete gel Western-blot associated with Figure 3A
Cabrera-Rodríguez, R., *et al.*

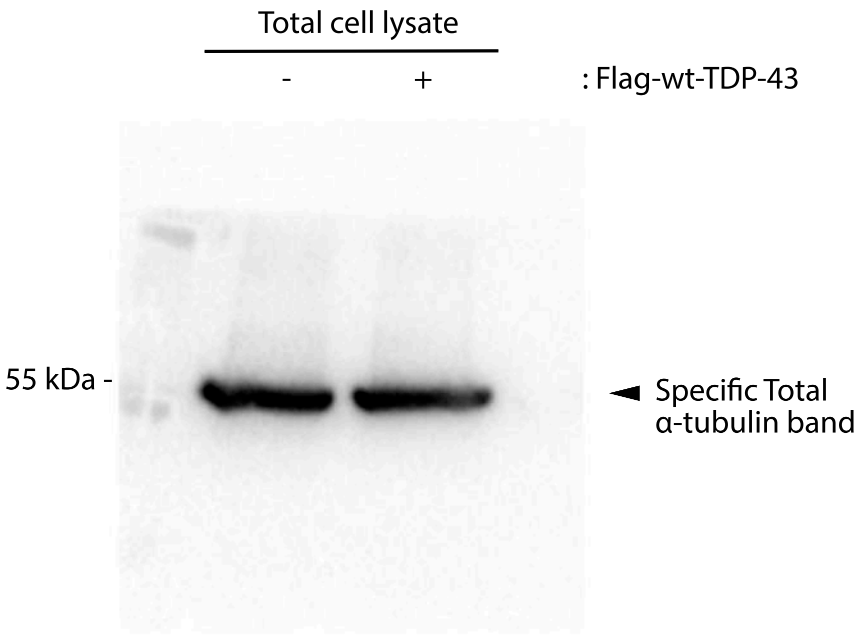

Figure S2. Replicate 2 HDAC6 complete gel Western-blot associated with Figure 3A
Cabrera-Rodríguez, R., *et al.*

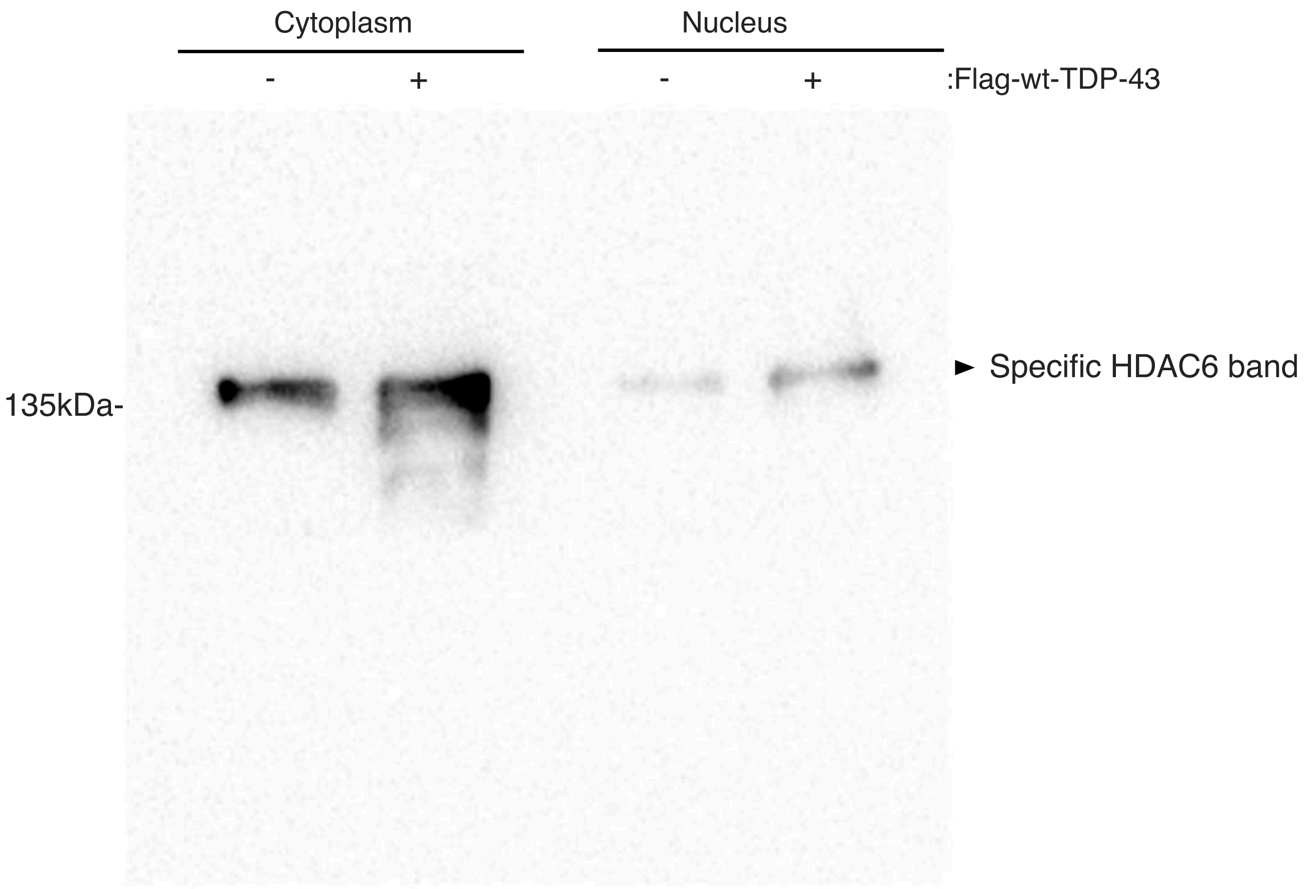

Figure S2. Replicate 2 TDP-43 complete gel Western-blot associated with Figure 3A
Cabrera-Rodríguez, R., *et al.*

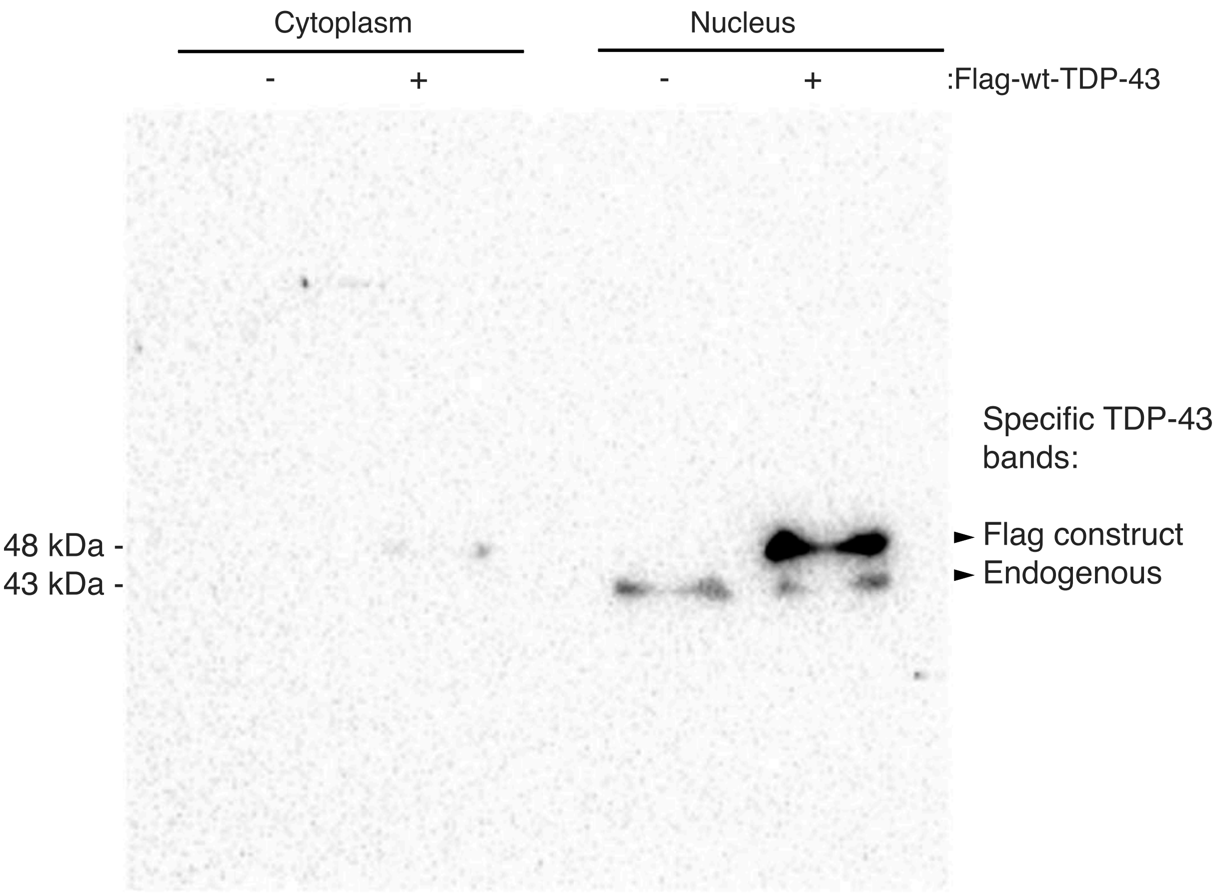

Figure S2. Replicate 2 Lamin B complete gel   Western-blot associated with Figure 3A
Cabrera-Rodríguez, R., *et al.*

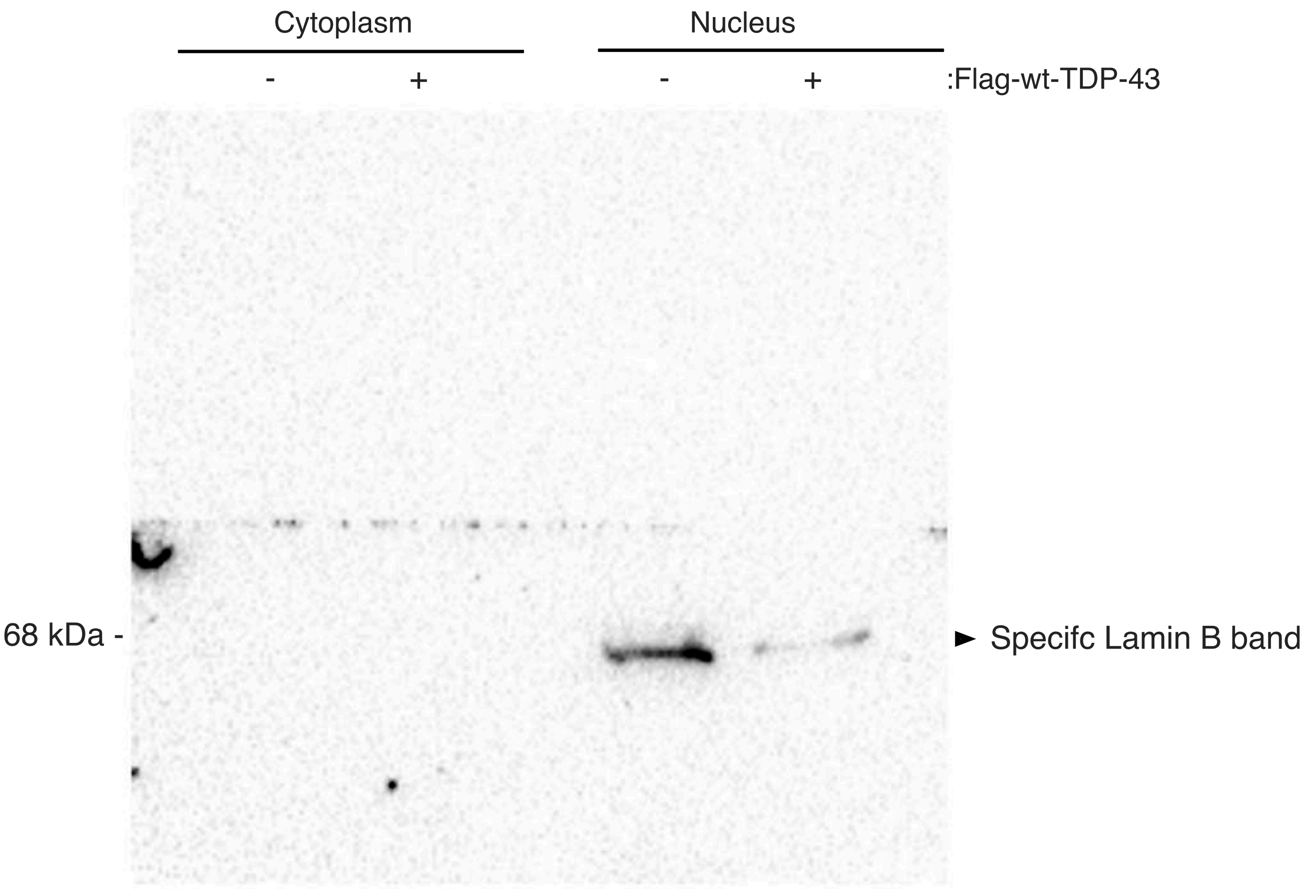

Figure S2. Replicate 2 Acetylated α-tubulin complete gel Western-blot associated with Figure 3A

Cabrera-Rodríguez, R., *et al.*

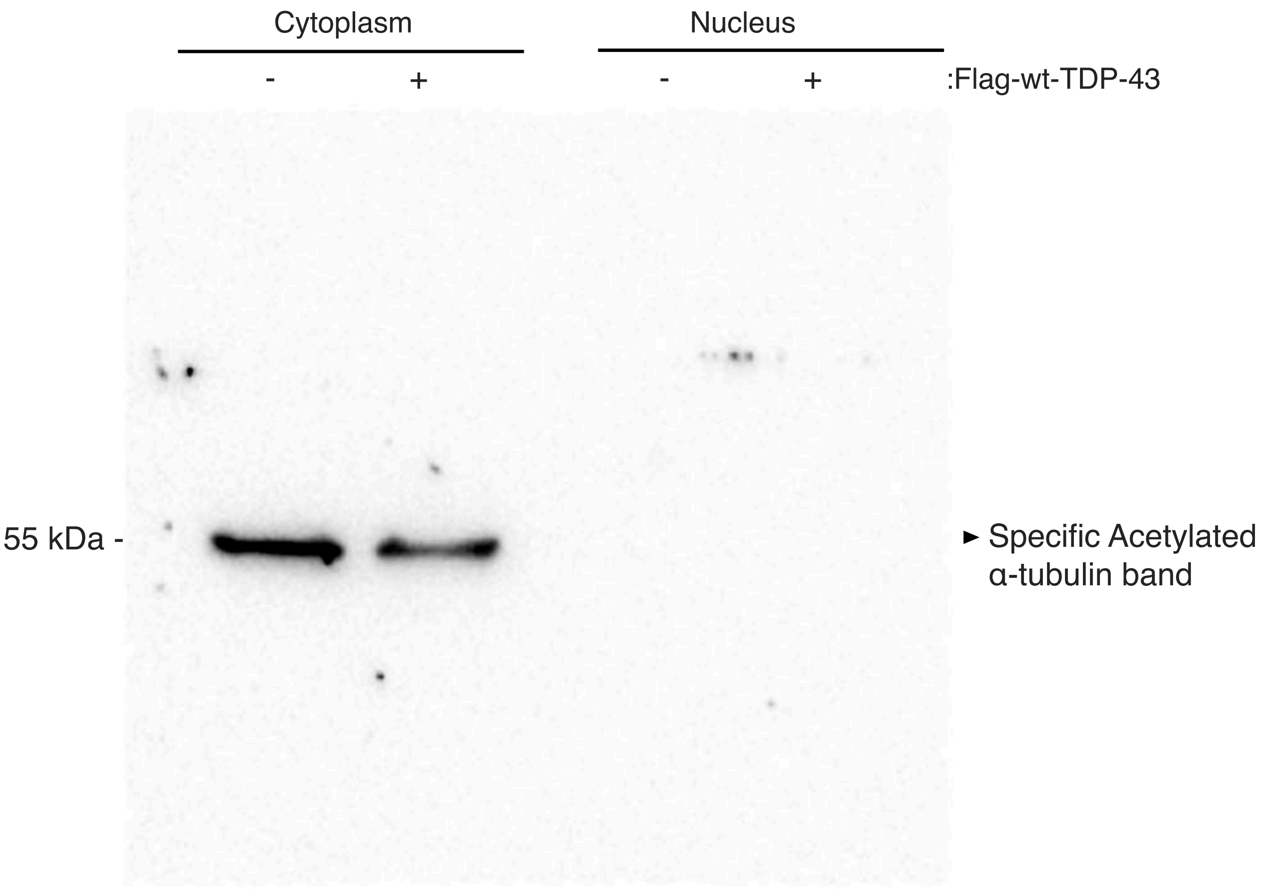

Figure S2. Replicate 2 Total α-tubulin complete gel Western-blot associated with Figure 3A

Cabrera-Rodríguez, R., *et al.*

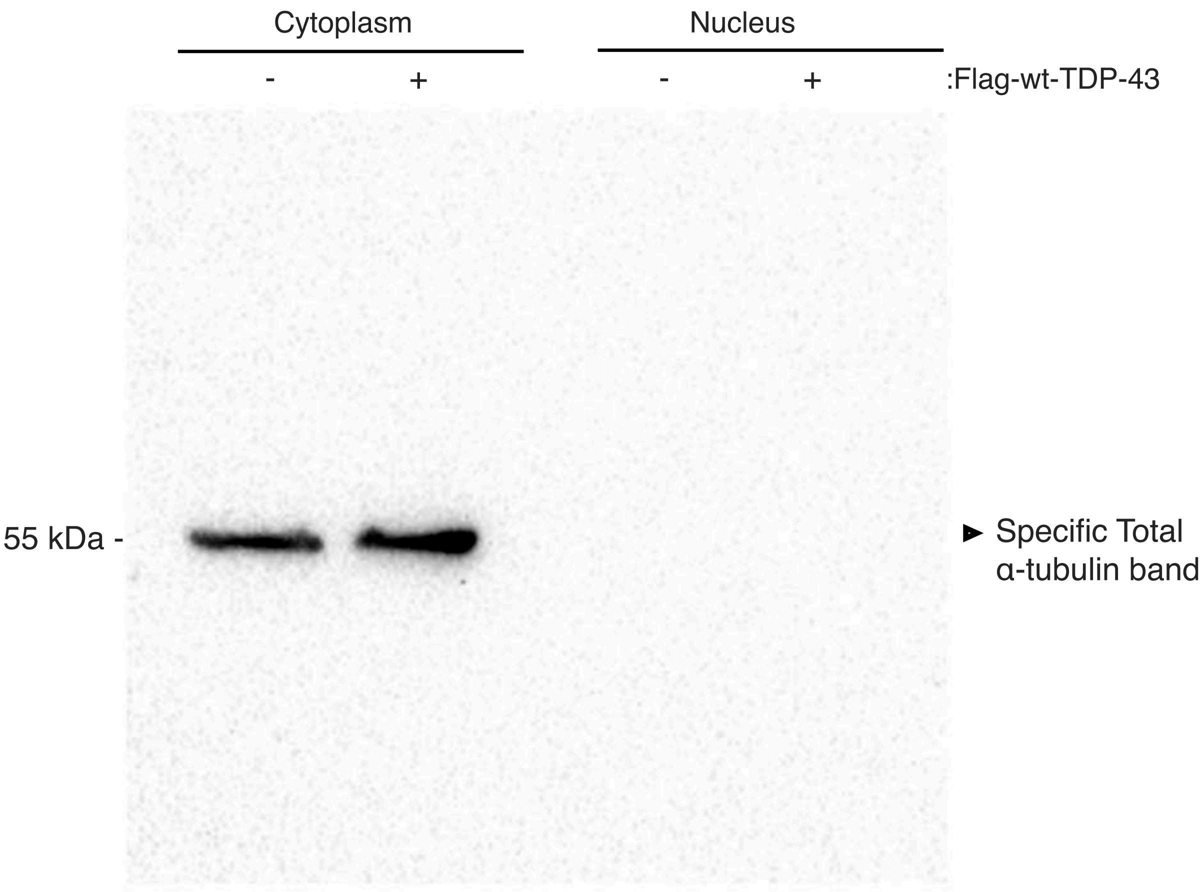

Figure S2. Replicate 3 HDAC6 complete gel

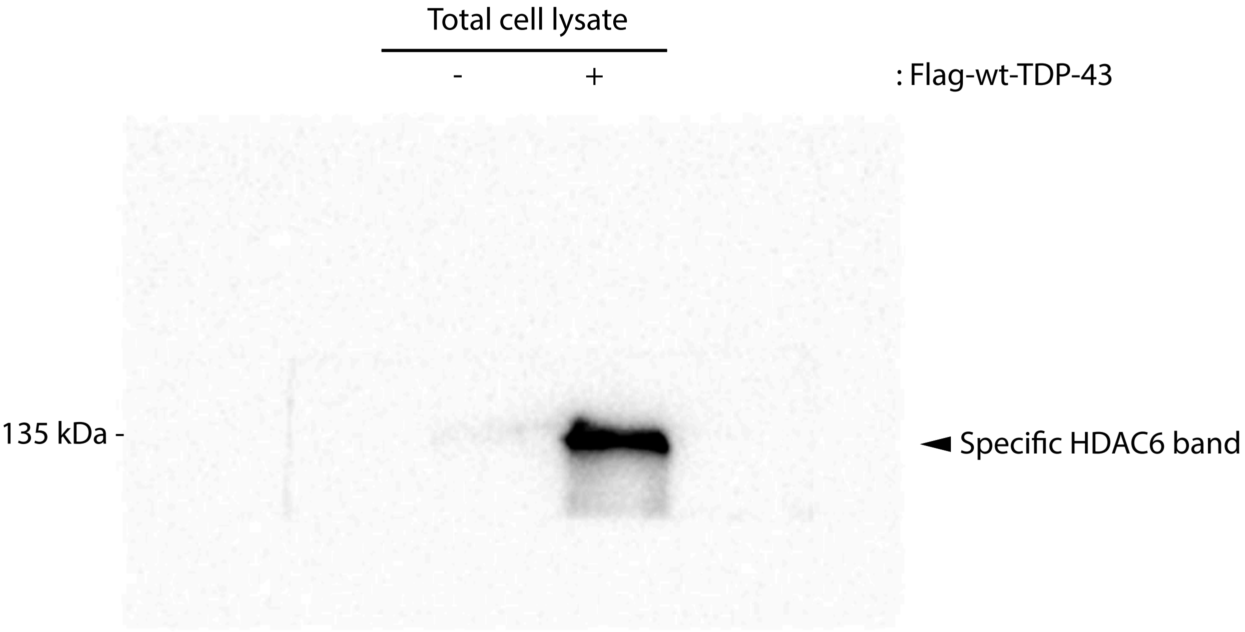

Figure S2. Replicate 3 TDP-43 complete gel Western-blot associated with Figure 3A

Cabrera-Rodríguez, R., *et al.*

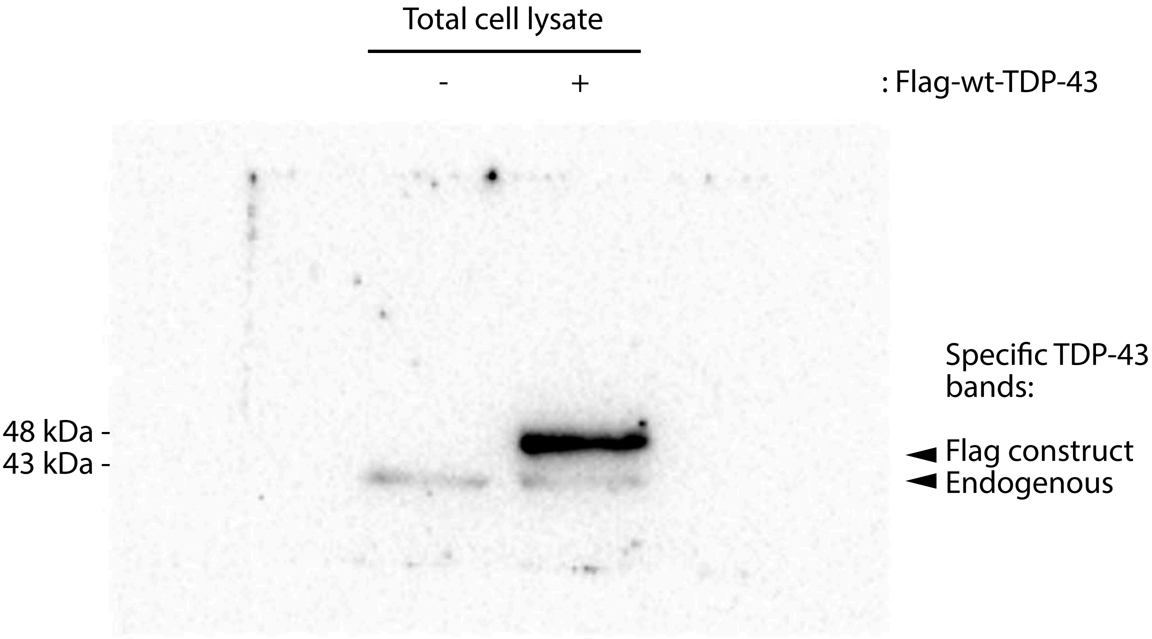

Figure S2. Replicate 3 Acetylated α-tubulin complete gel   Western-blot associated with Figure 3A
Cabrera-Rodríguez, R., *et al.*

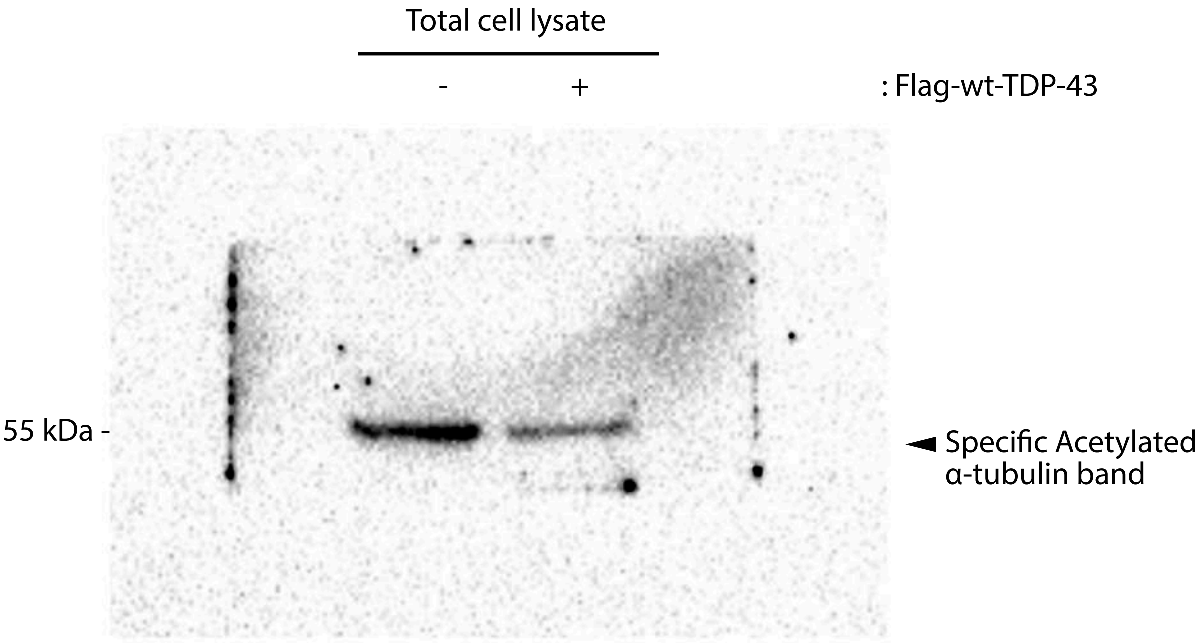

Figure S2. Replicate 3 Total α-tubulin complete gel   Western-blot associated with Figure 3A
Cabrera-Rodríguez, R., *et al.*

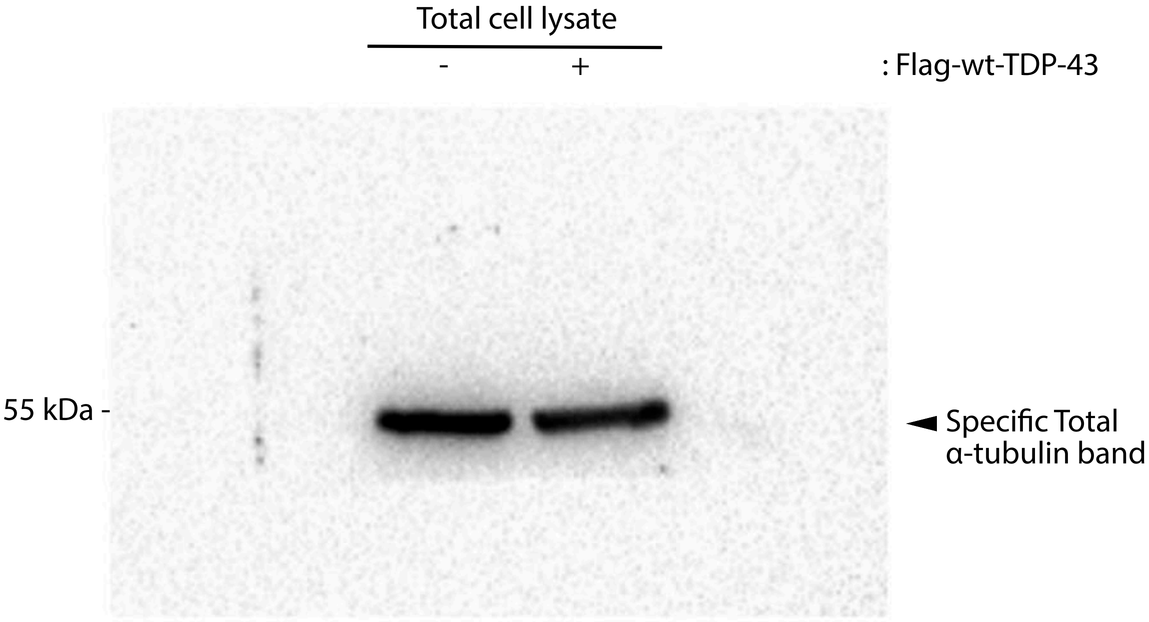

Figure S2. Replicate 3 HDAC6 complete gel Western-blot associated with Figure 3A
Cabrera-Rodríguez, R., *et al.*

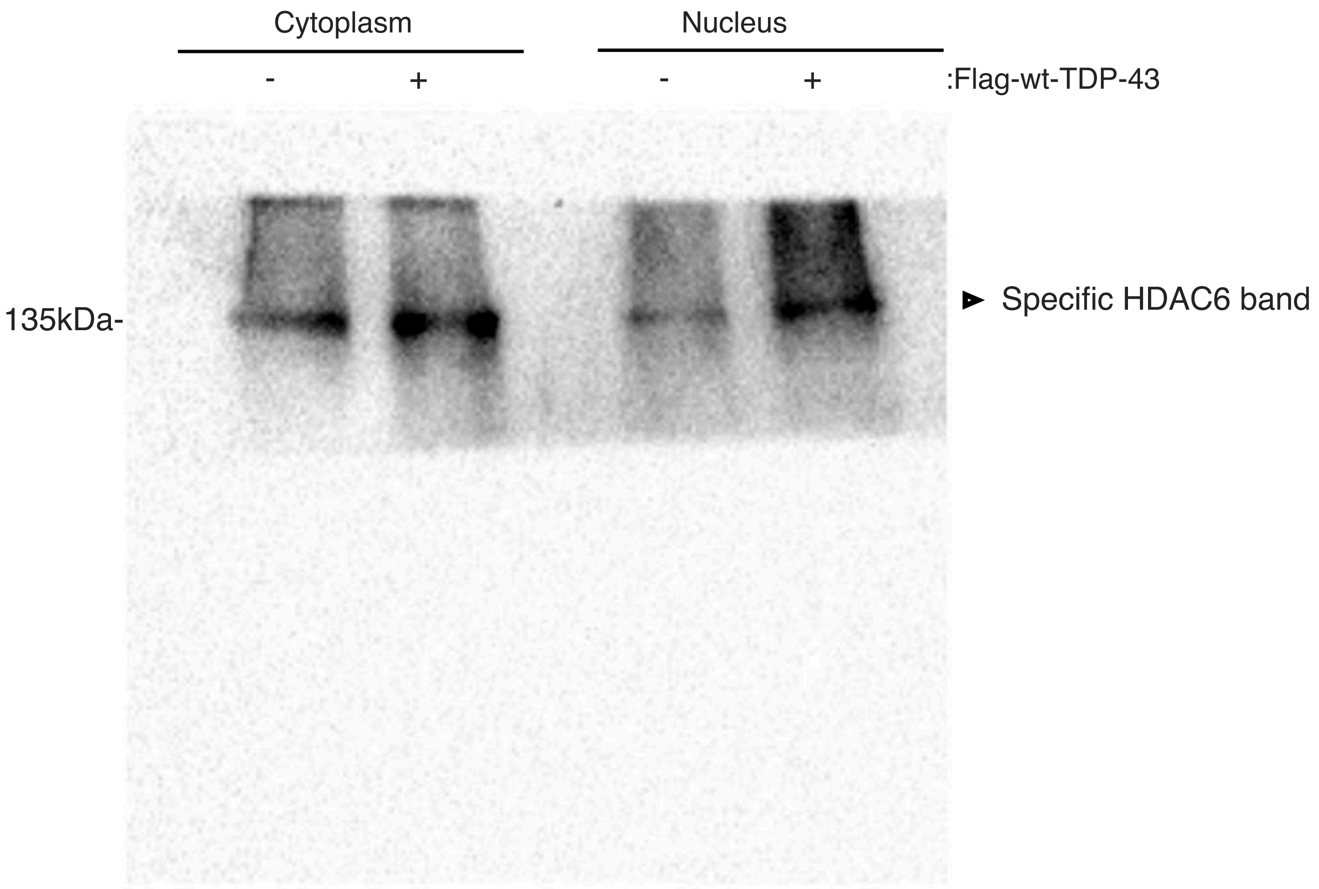

Figure S2. Replicate 3 TDP-43 complete gel   Western-blot associated with Figure 3A
Cabrera-Rodríguez, R., *et al.*

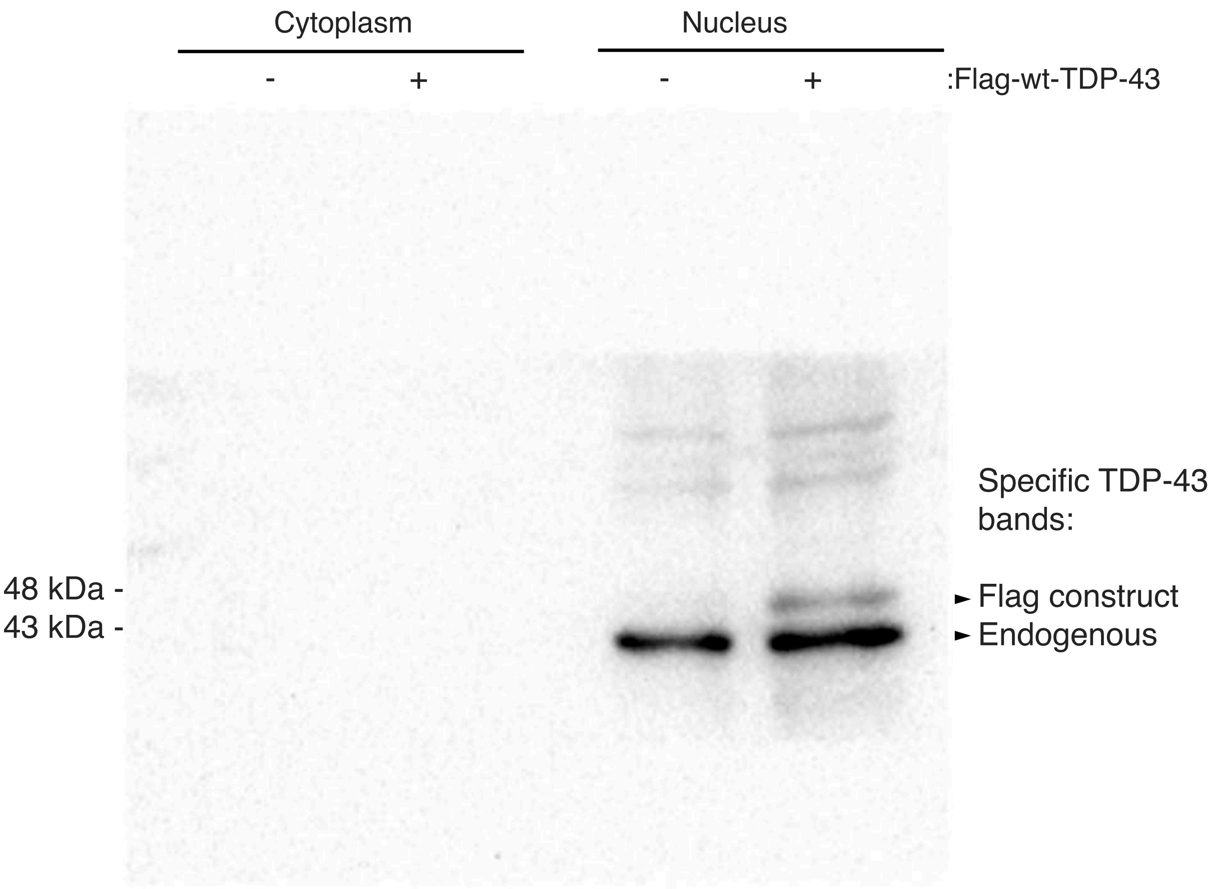

Figure S2. Replicate 3 Lamin B complete gel   Western-blot associated with Figure 3A
Cabrera-Rodríguez, R., *et al.*

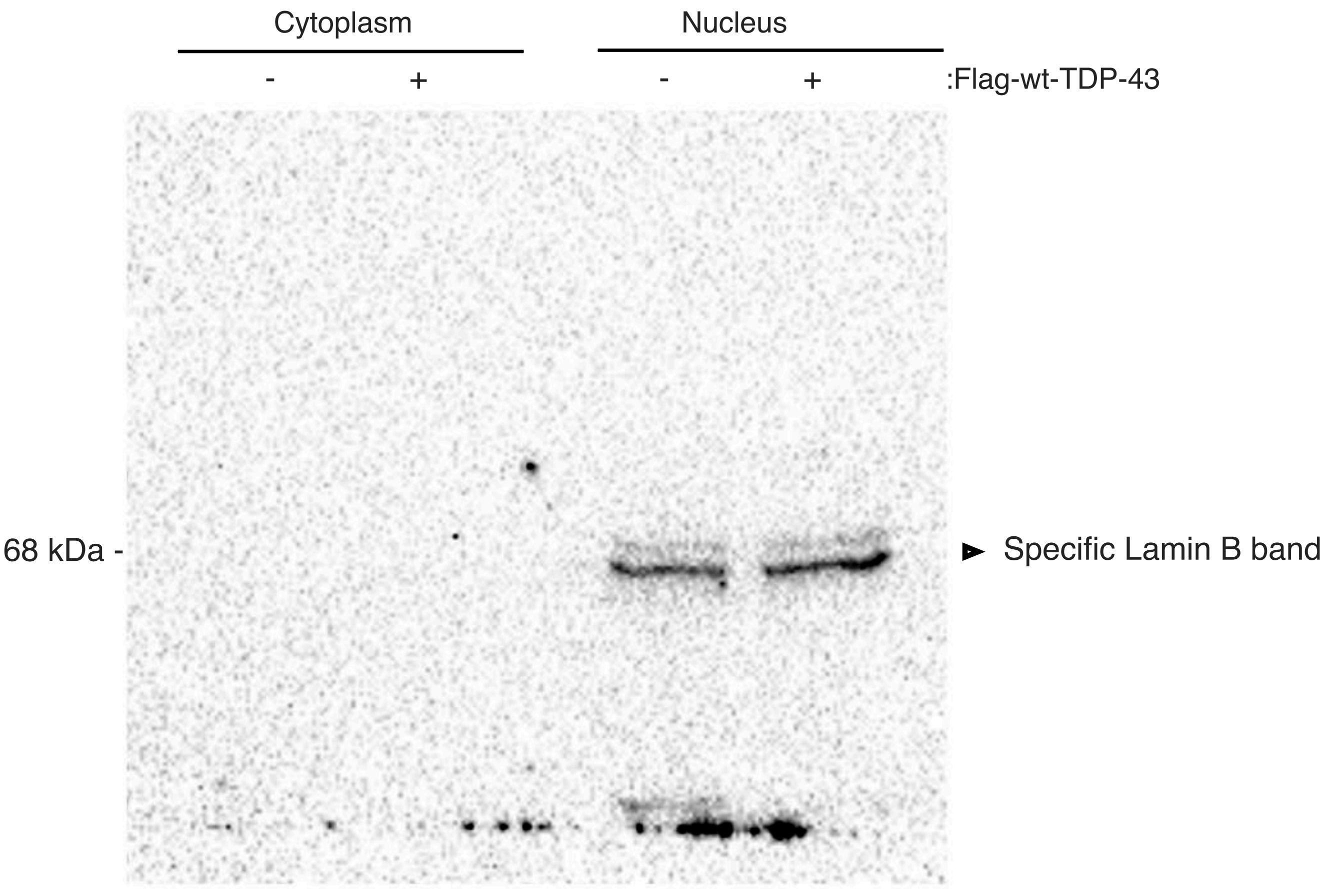

Figure S2. Replicate 3 Acetylated α-tubulin complete gel Western-blot associated with Figure 3A

Cabrera-Rodríguez, R., *et al.*

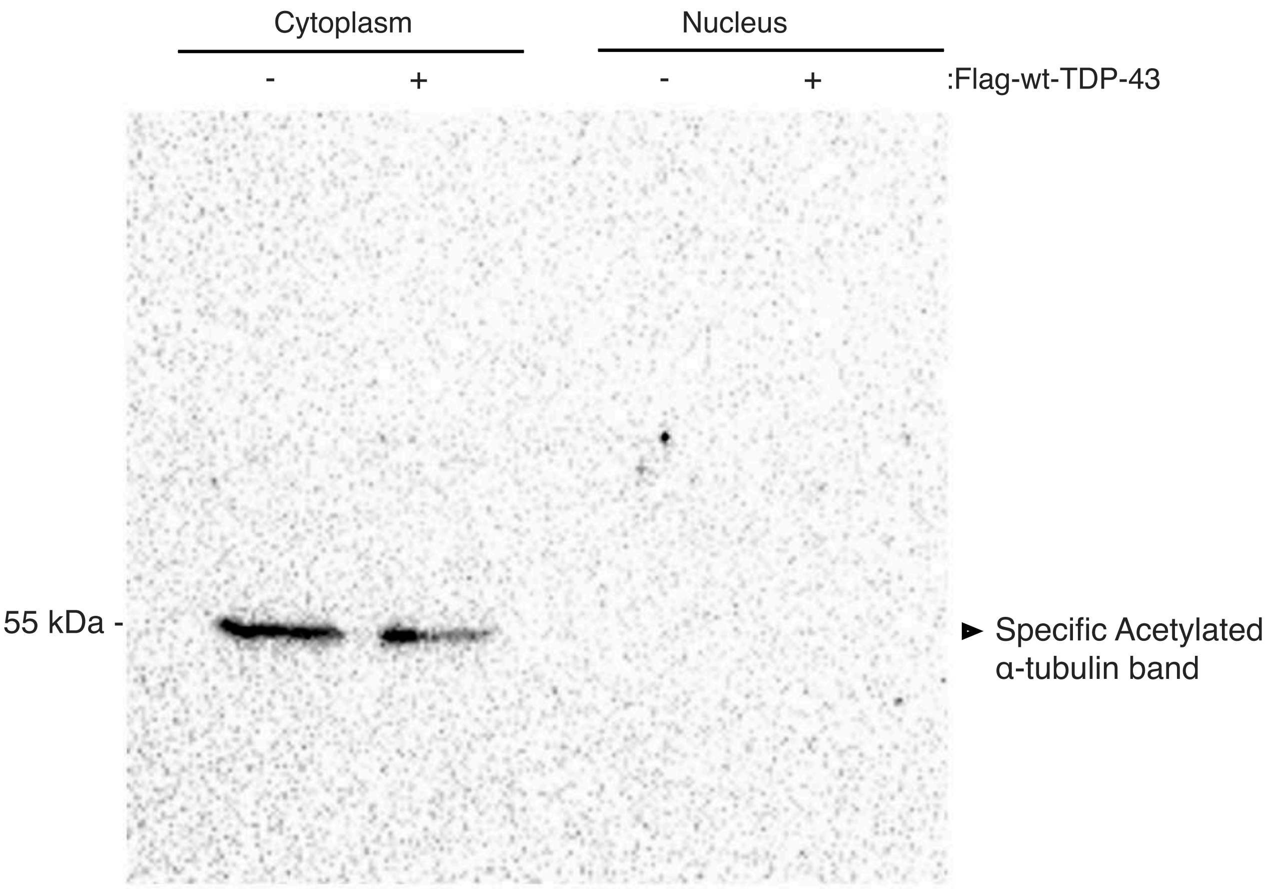

Figure S2. Replicate 3 Total α-tubulin complete gel Western-blot associated with Figure 3A

Cabrera-Rodríguez, R., *et al.*

Cytoplasm Nucleus

- + - + :Flag-wt-TDP-43

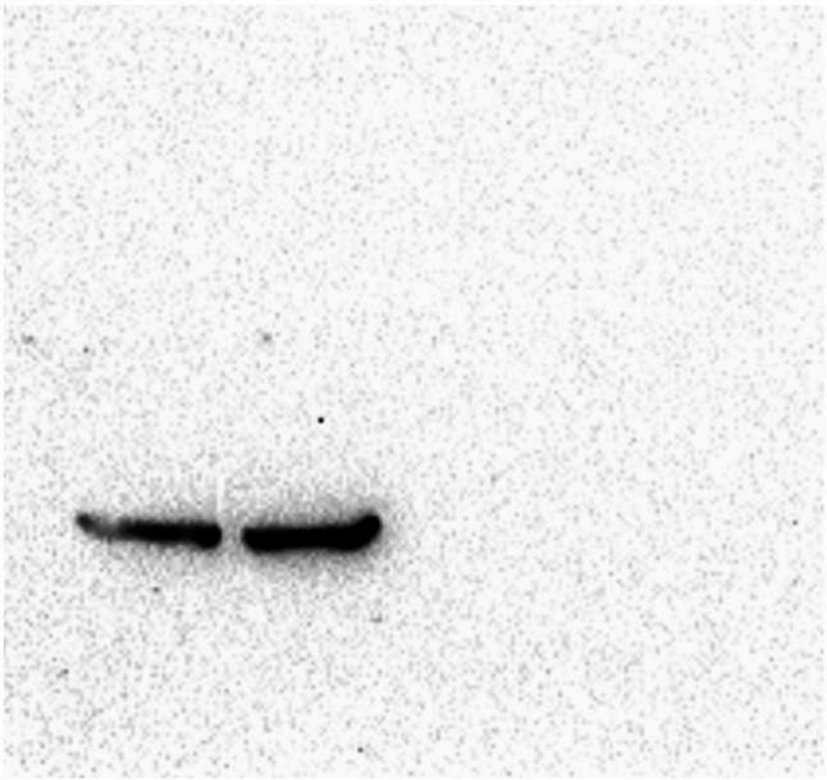

55 kDa -

► Specific Total α-tubulin band

Figure S3. Western-blot replicates associated with Figure 4A Cabrera-Rodríguez R., *et al.*

Replicate 1 as figure format

A

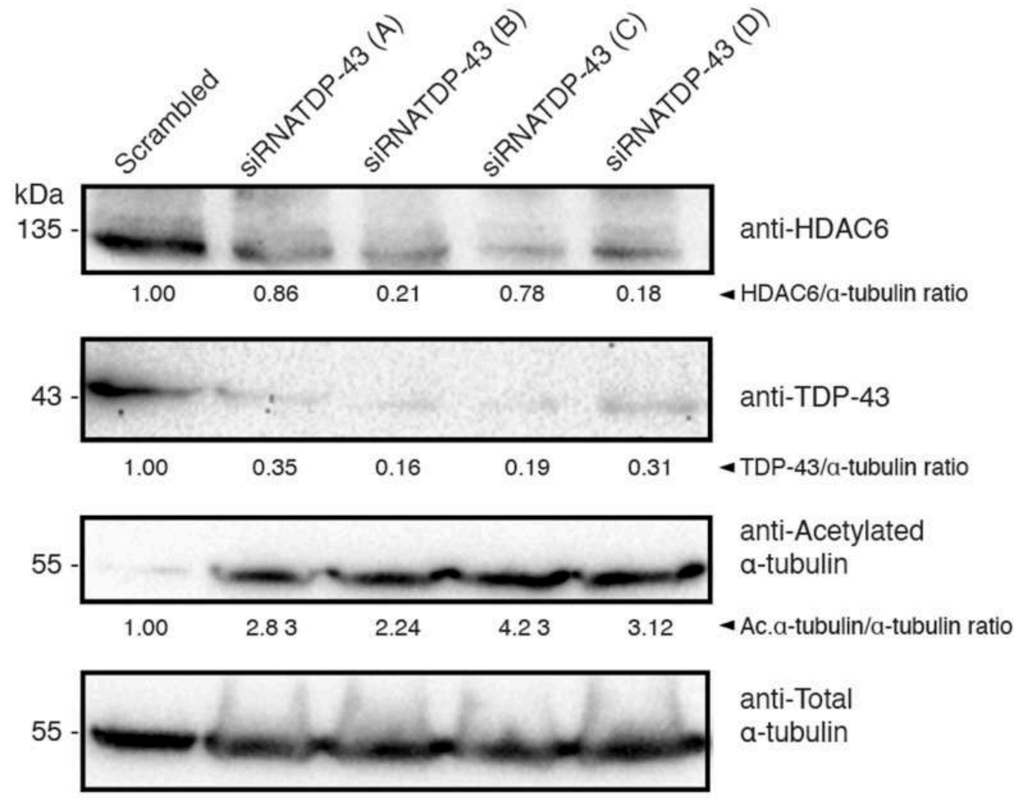

Replicate 2 as figure format

A

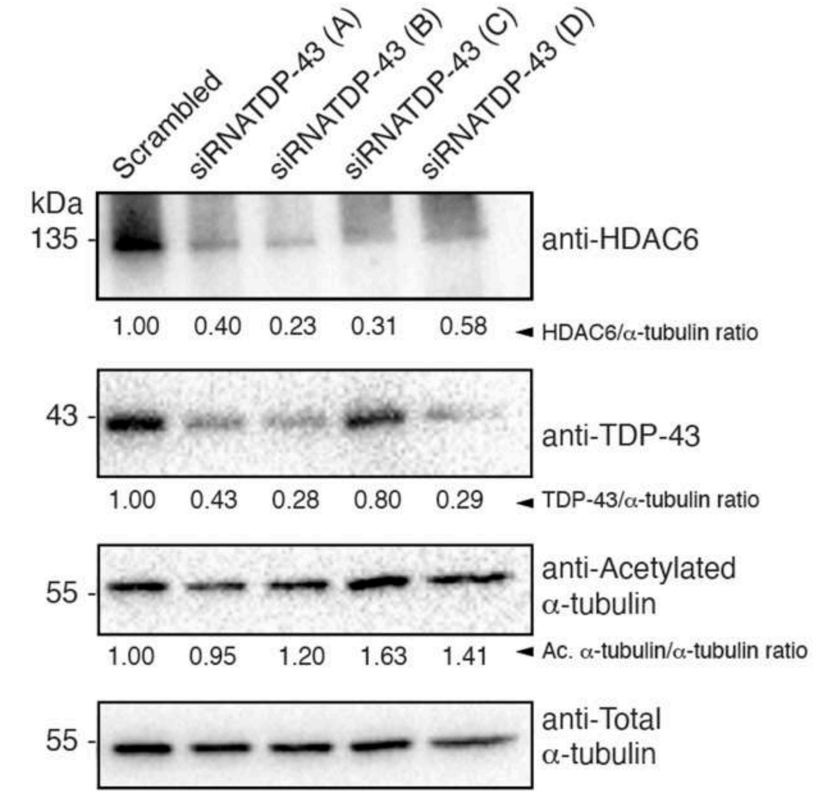

Replicate 3 as figure format

A

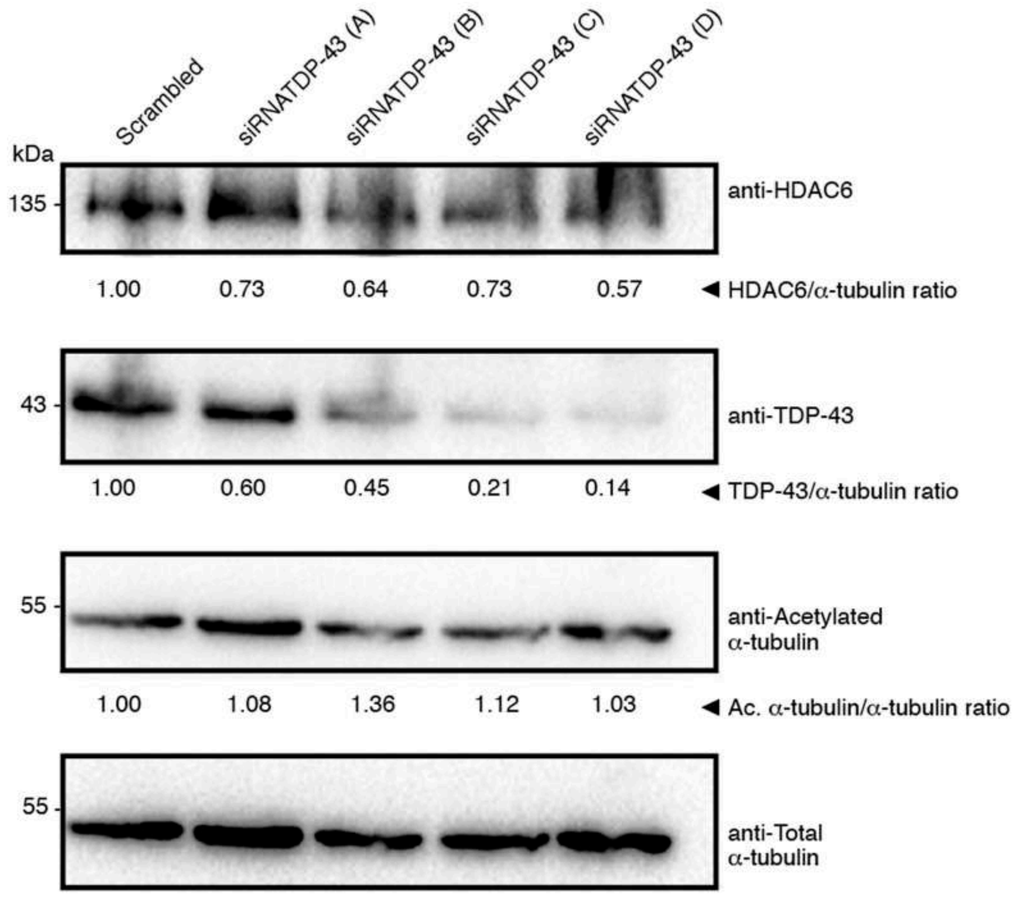

Figure S3. Replicate 1 HDAC6 complete gel Western-blot associated with Figure 4A
Cabrera-Rodríguez, R., *et al.*

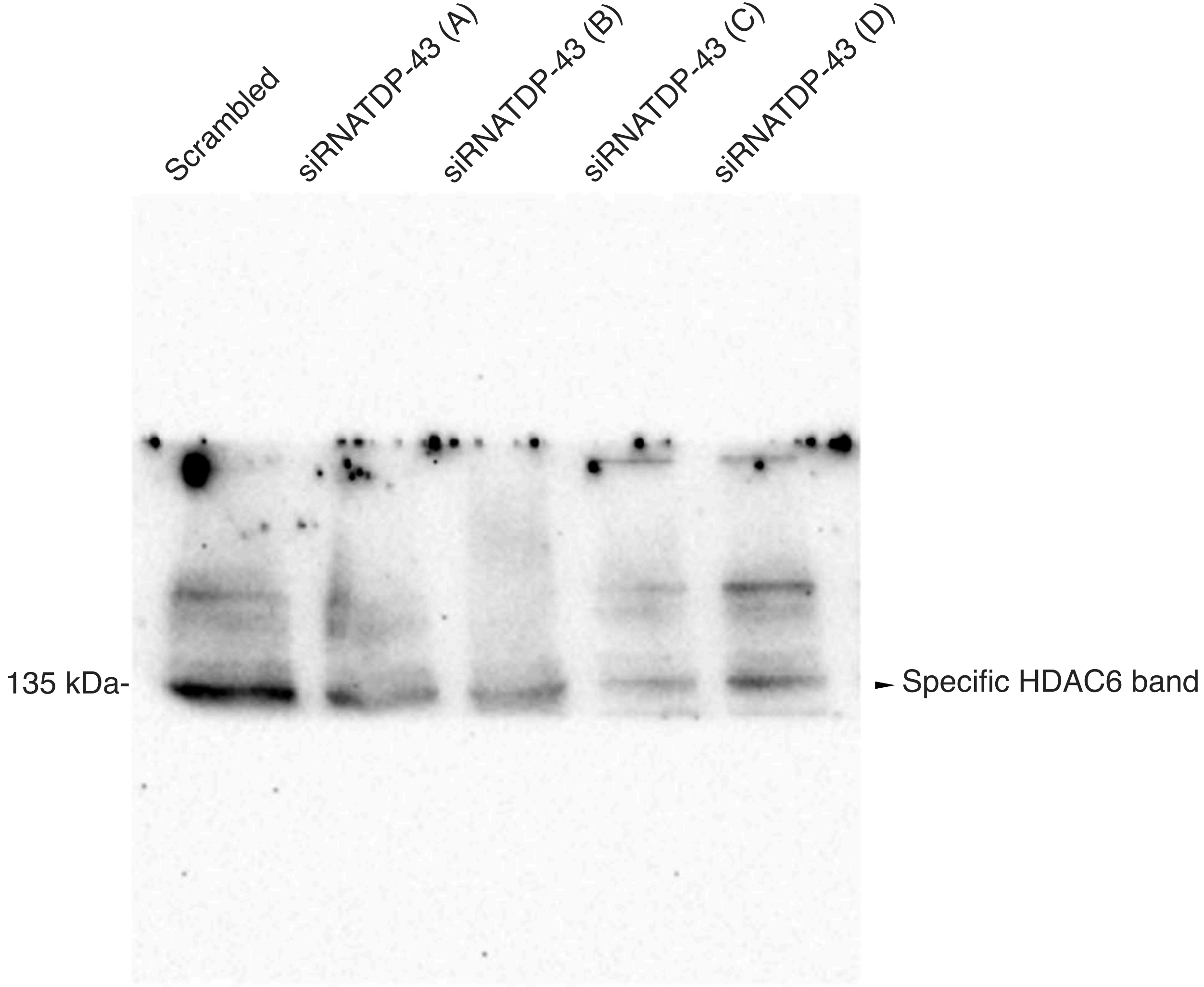

Figure S3. Replicate 1 TDP-43 complete gel Western-blot associated with Figure 4A

Cabrera-Rodríguez, R., *et al.*

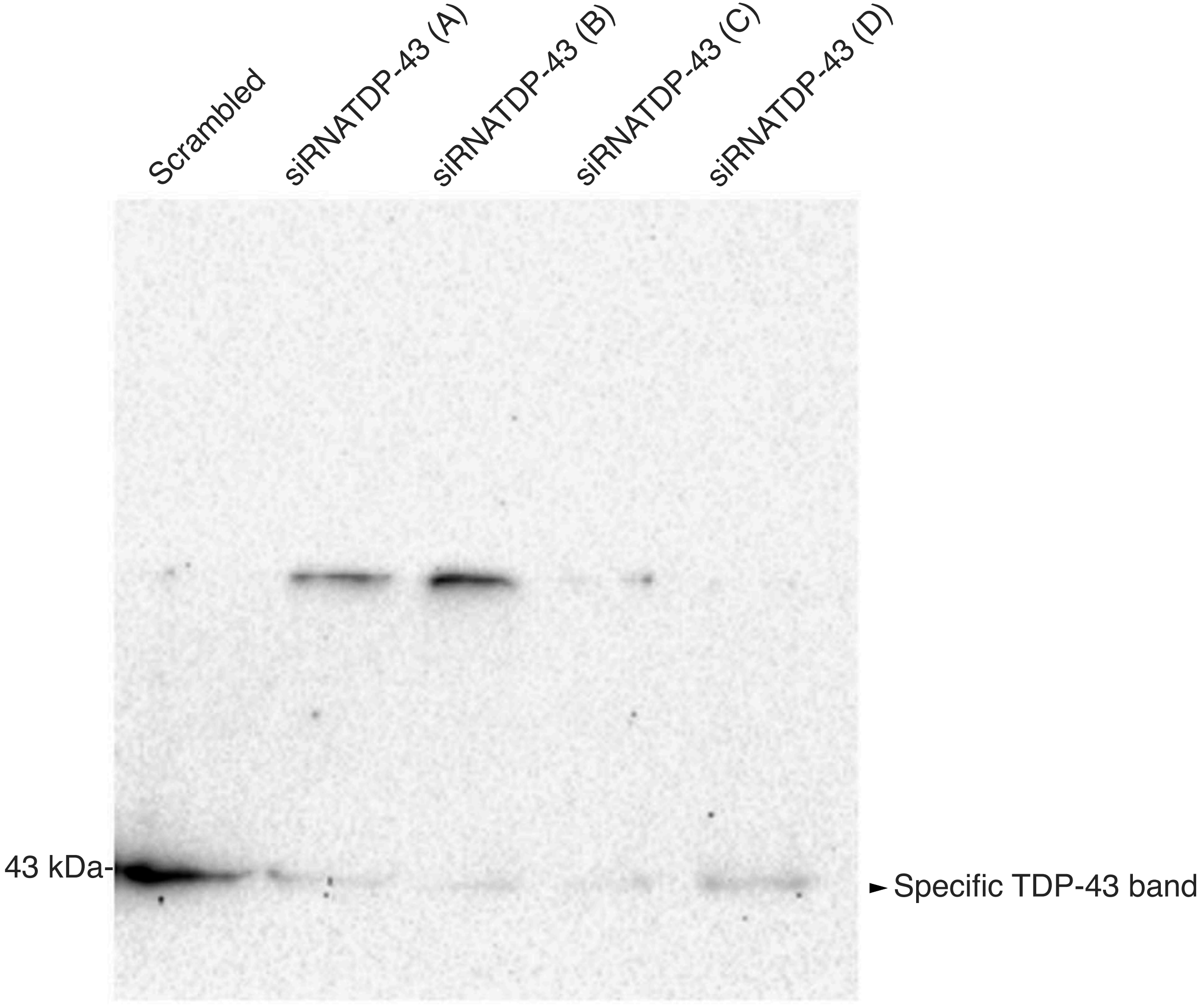

Figure S3. Replicate 1 Acetylated α-tubulin complete gel Western-blot associated with Figure 4A
Cabrera-Rodríguez, R., *et al.*

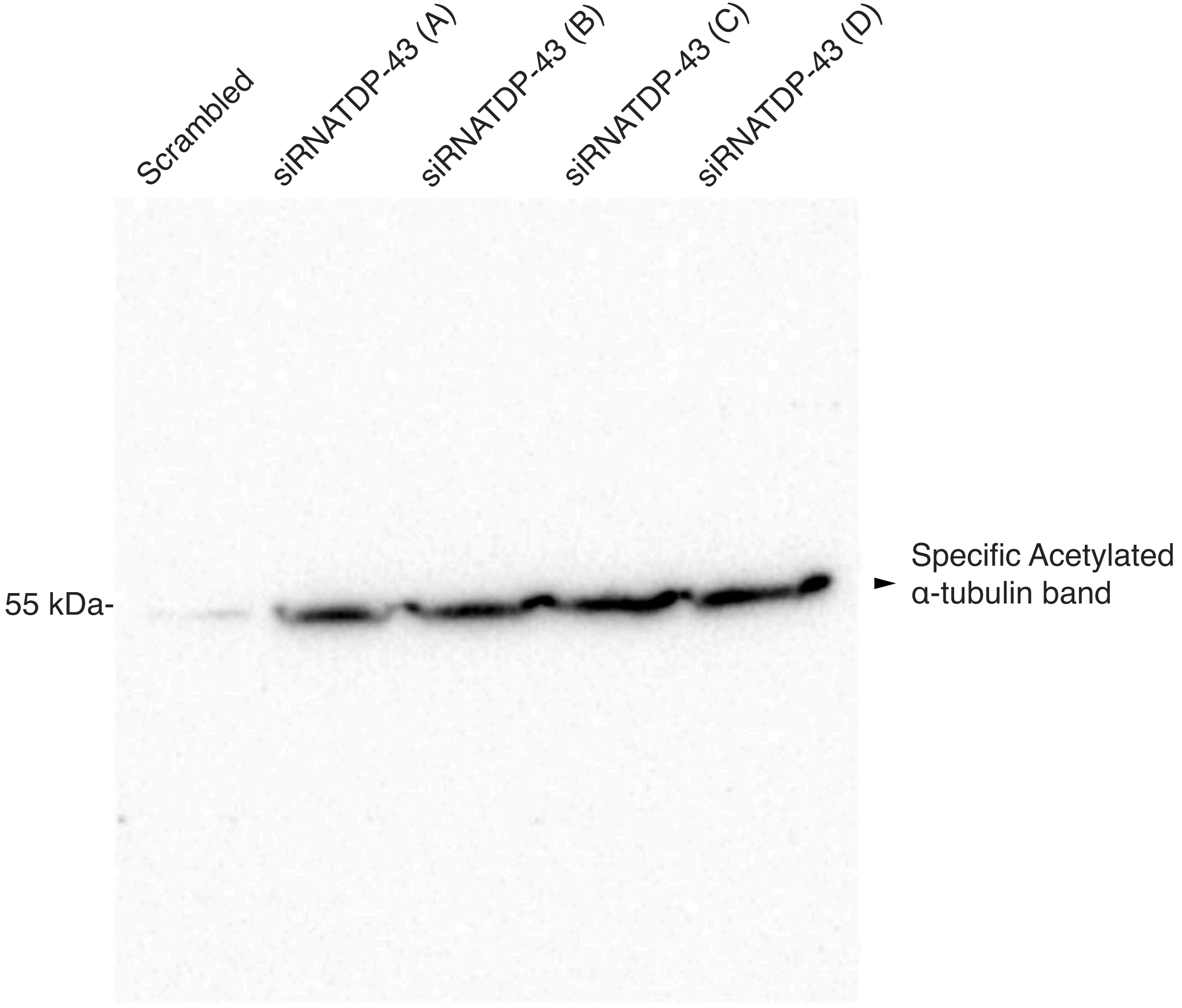

Figure S3. Replicate 1 Total α-tubulin complete gel   Western-blot associated with Figure 4A
Cabrera-Rodríguez, R., *et al.*

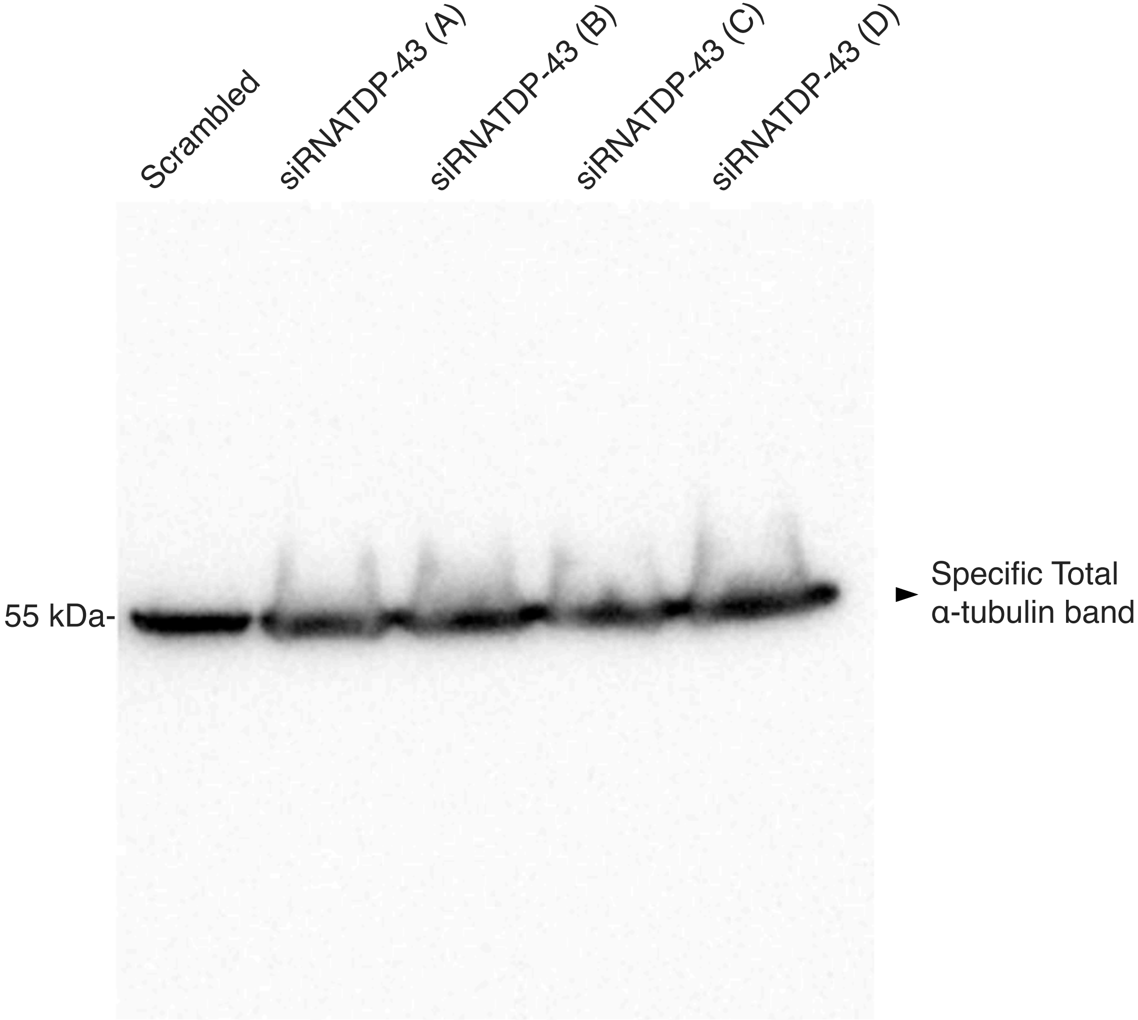

Figure S3. Replicate 2 HDAC6 complete gel Western-blot associated with Figure 4A

Cabrera-Rodríguez, R., *et al.*

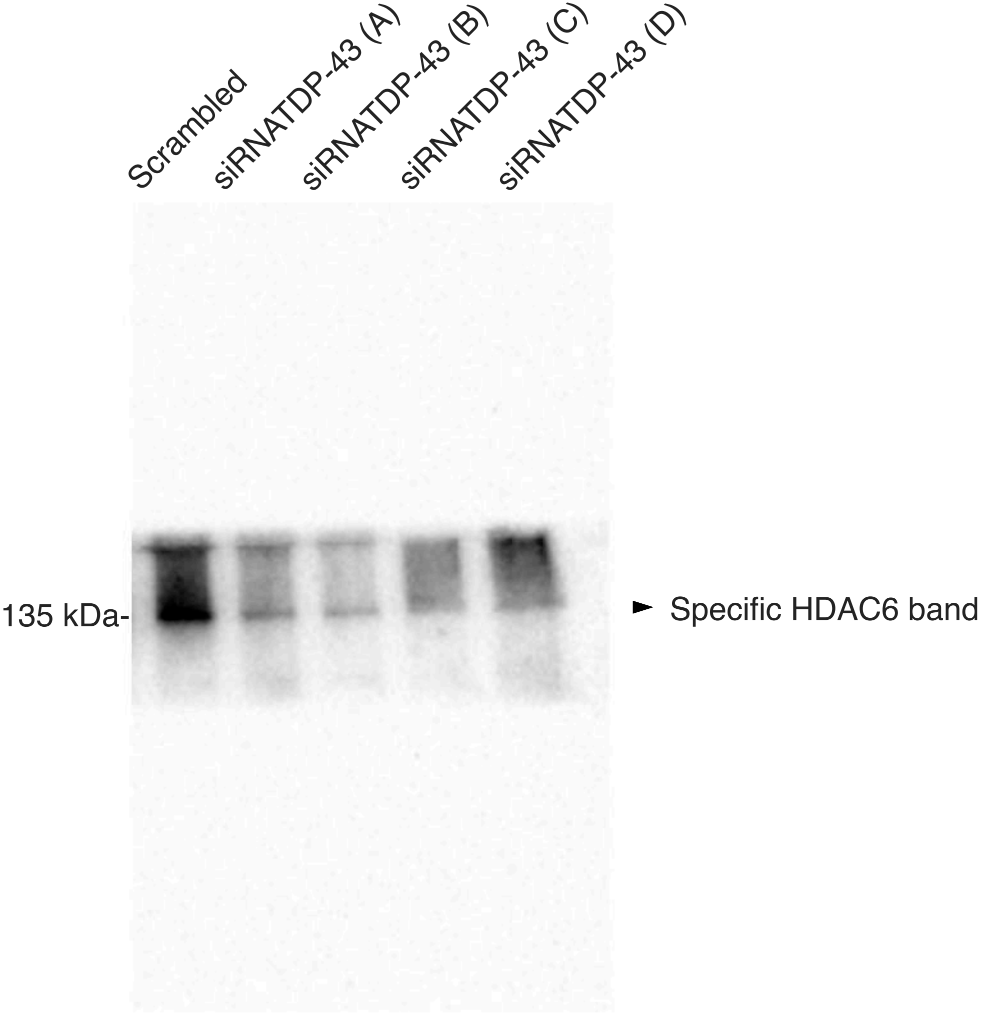

Figure S3. Replicate 2 TDP-43 complete gel   Western-blot associated with Figure 4A

Cabrera-Rodríguez, R., *et al.*

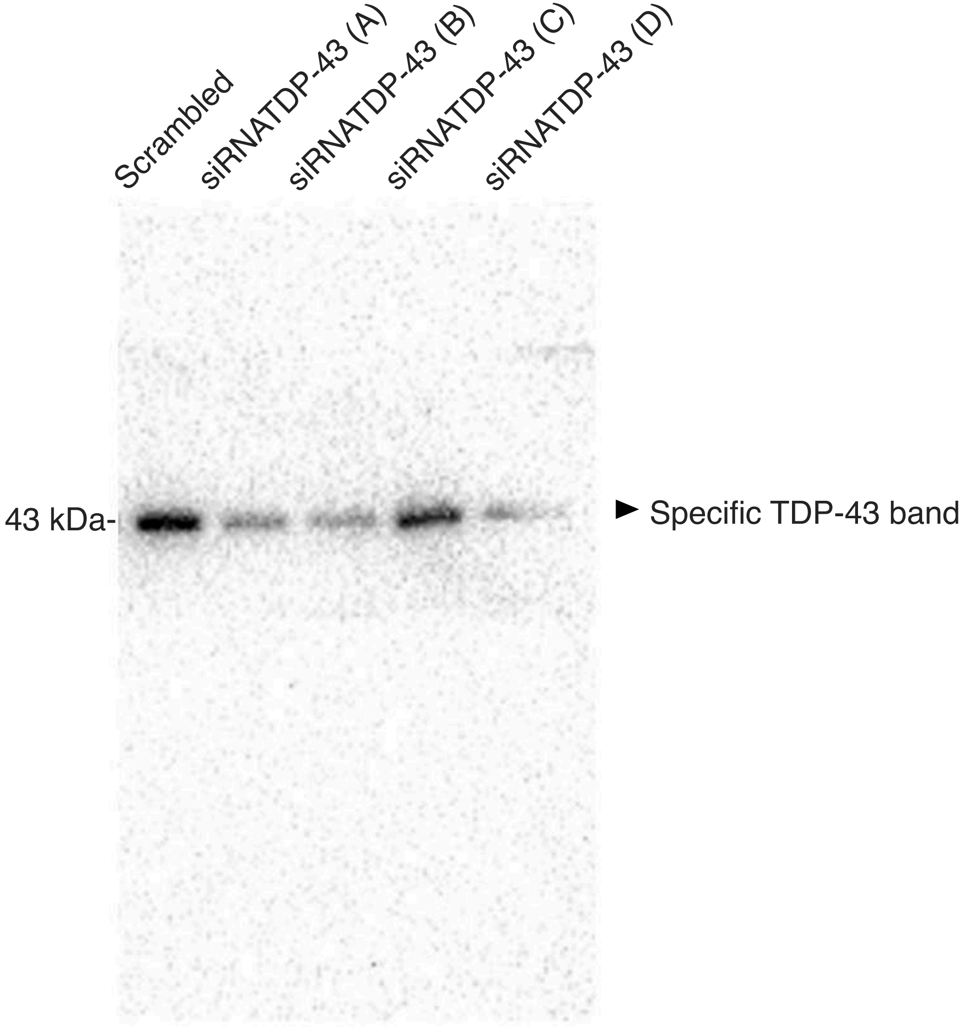

Figure S3. Replicate 2 Acetylated α-tubulin complete gel Western-blot associated with Figure 4A

Cabrera-Rodríguez, R., *et al.*

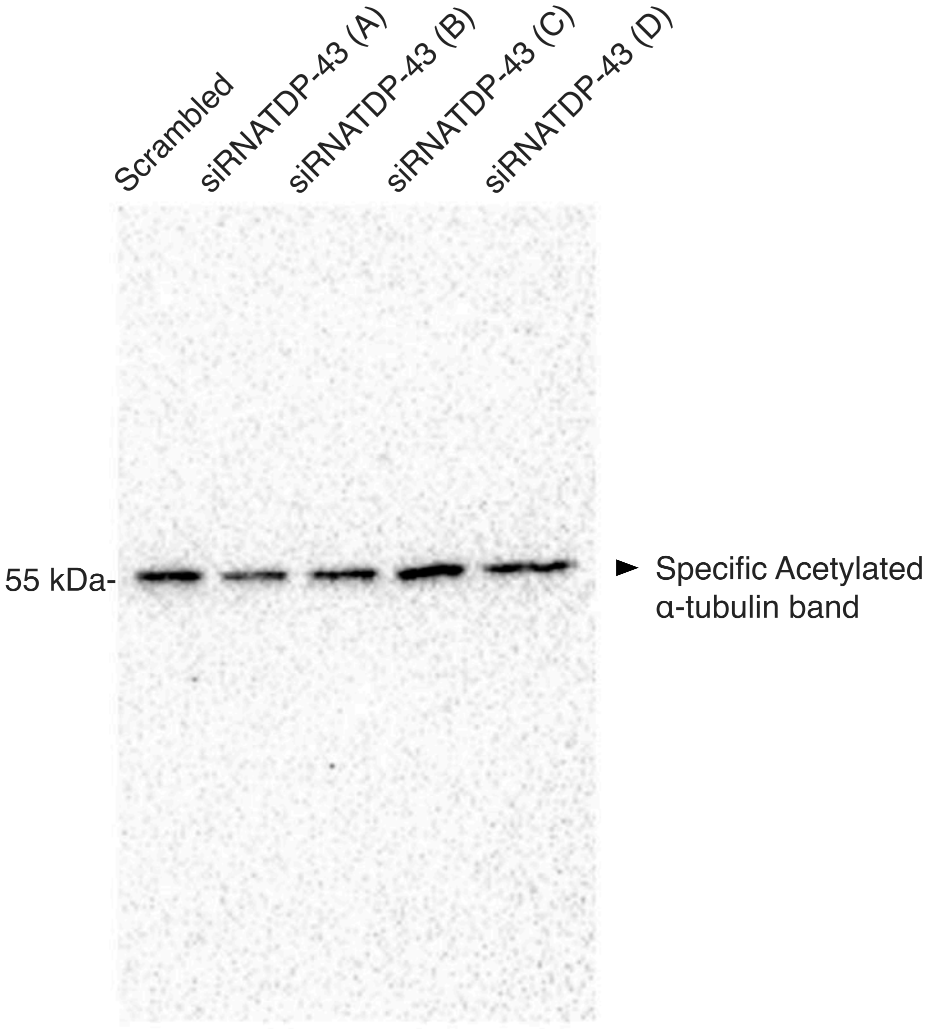

Figure S3. Replicate 2 Total α-tubulin complete gel Western-blot associated with Figure 4A
Cabrera-Rodríguez, R., *et al.*

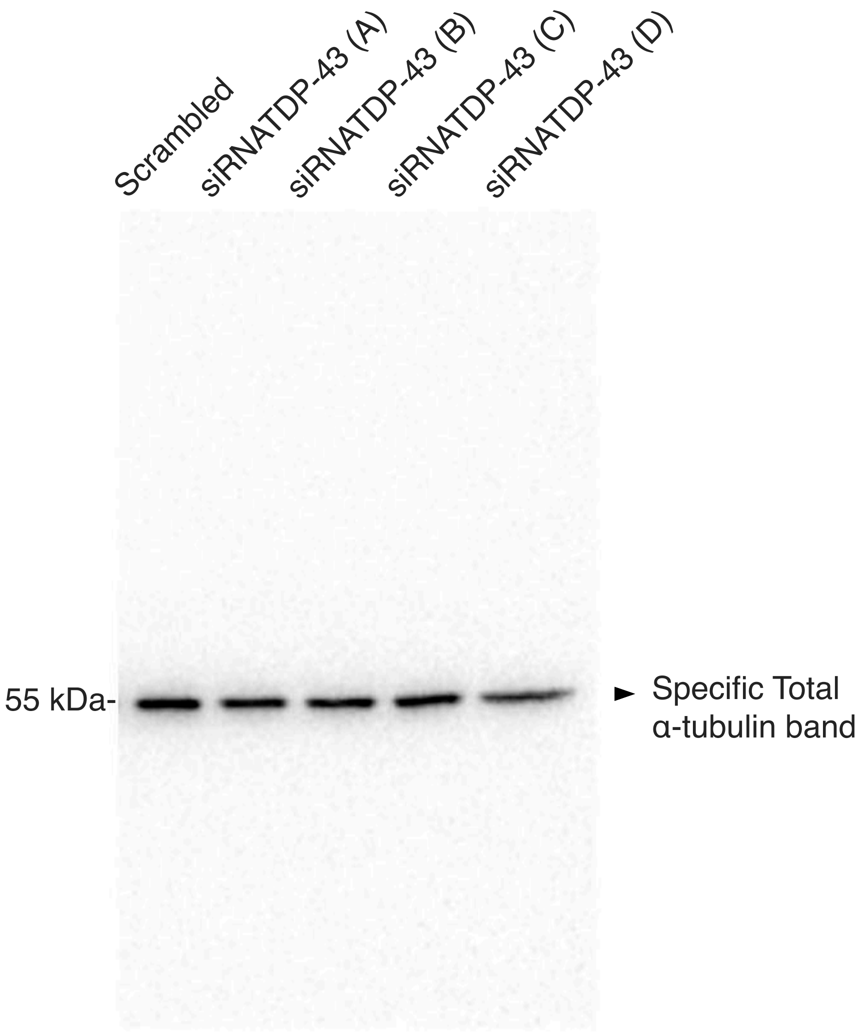

Figure S3. Replicate 3 HDAC6 complete gel Western-blot associated with Figure 4A

Cabrera-Rodríguez, R., *et al.*

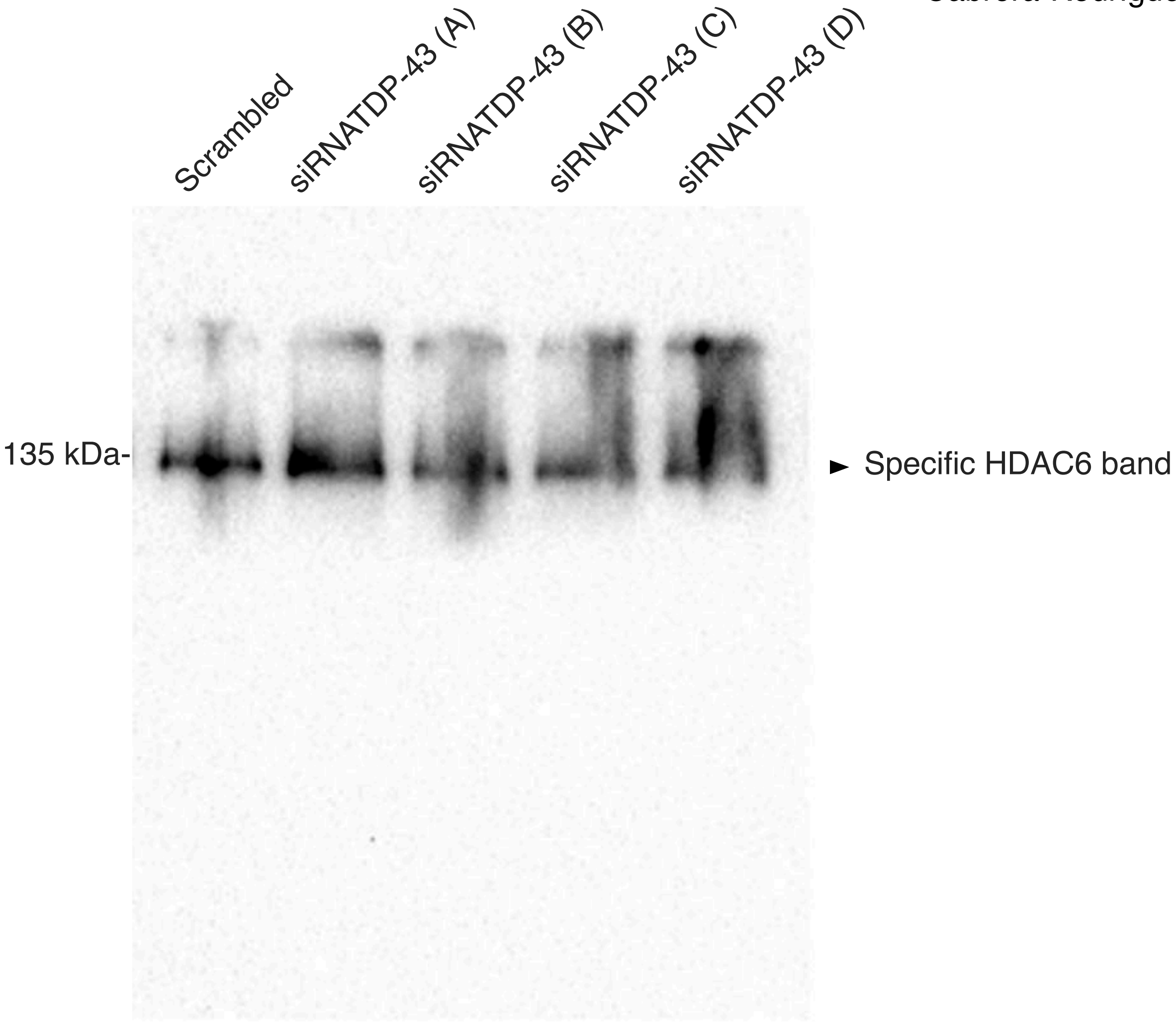

Figure S3. Replicate 3 TDP-43 complete gel Western-blot associated with Figure 4A

Cabrera-Rodríguez, R., *et al.*

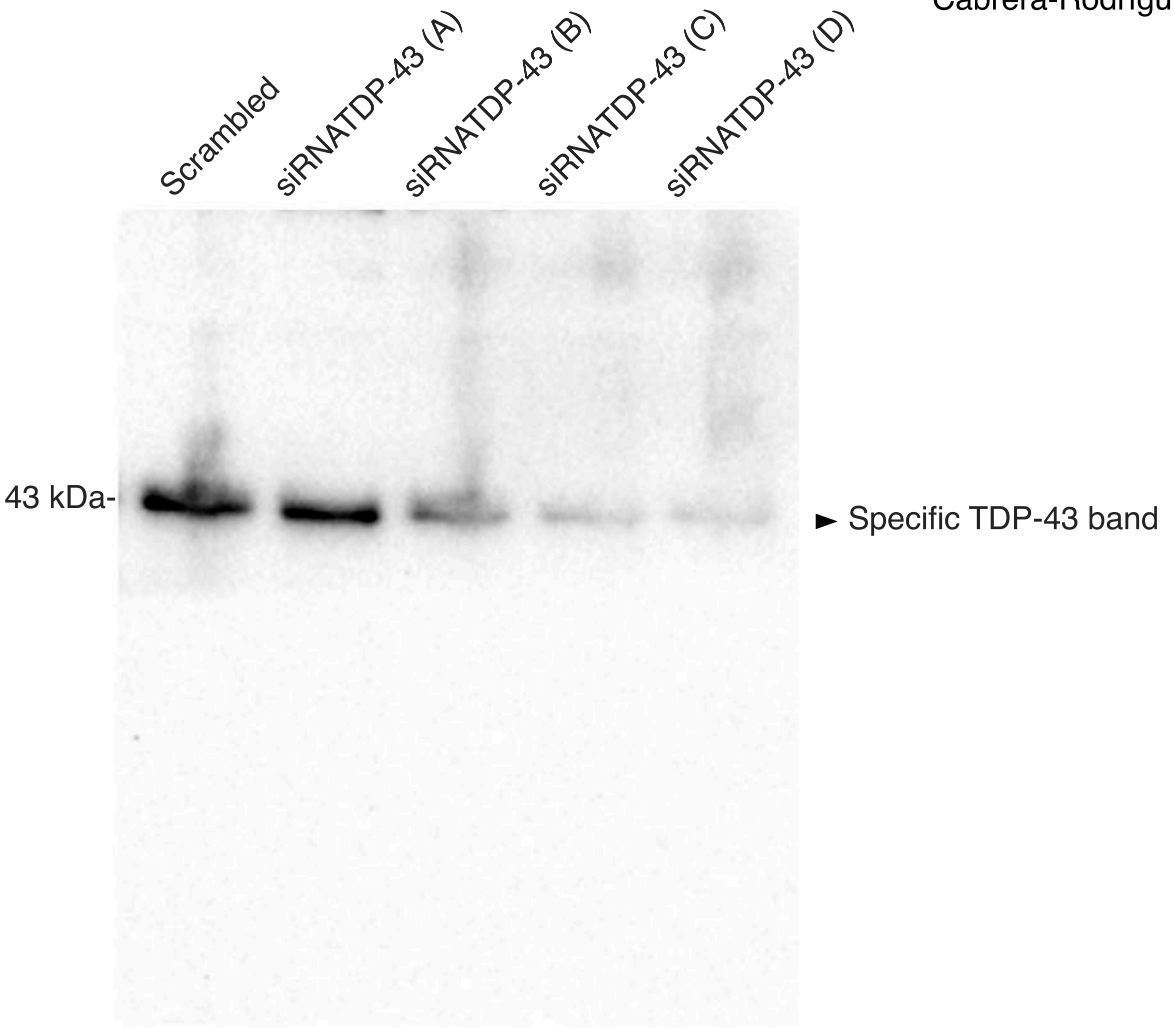

Figure S3. Replicate 3 Acetylated α-tubulin complete gel Western-blot associated with Figure 4A
Cabrera-Rodríguez, R., *et al.*

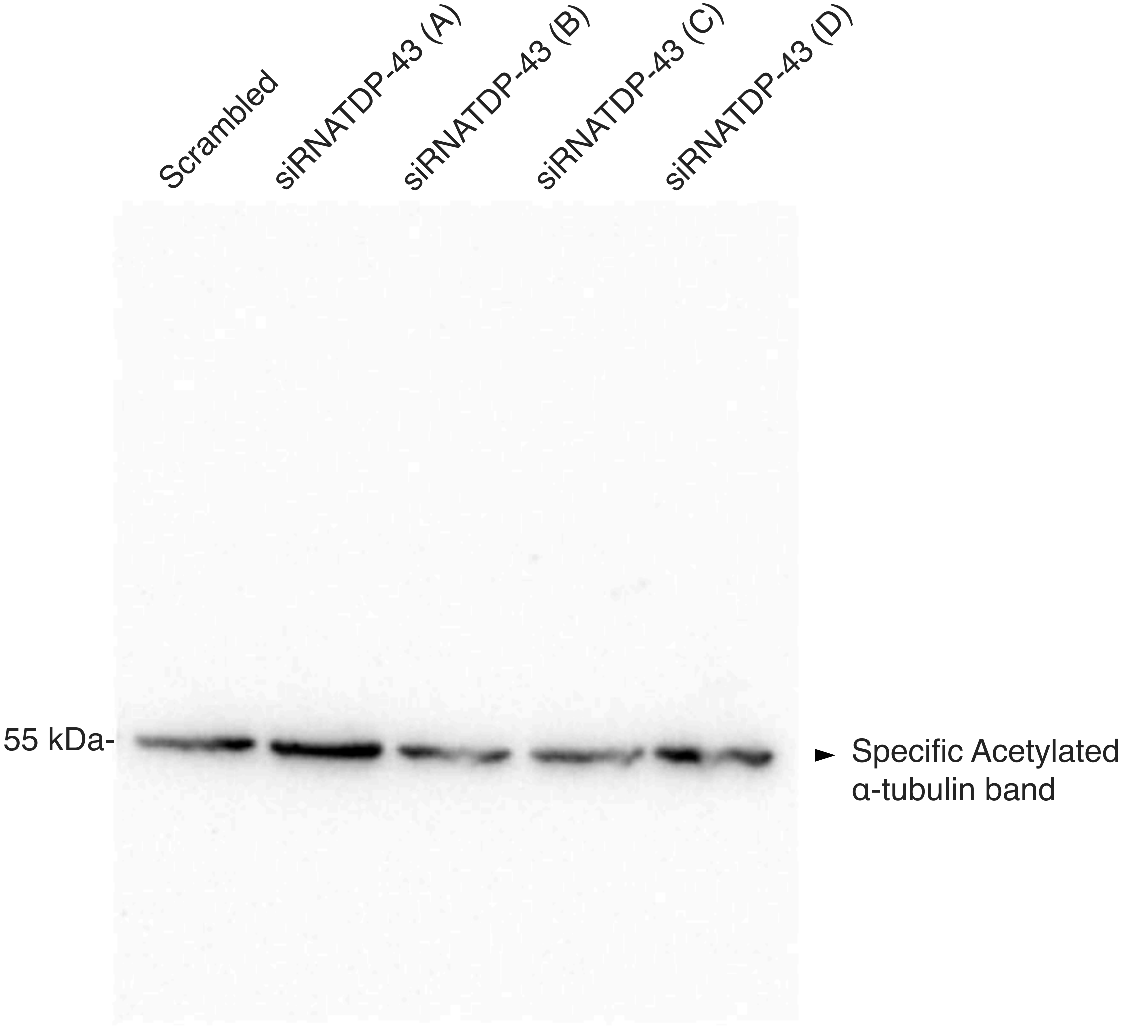

Figure S3. Replicate 3 Total α-tubulin complete gel   Western-blot associated with Figure 4A
Cabrera-Rodríguez, R., *et al.*

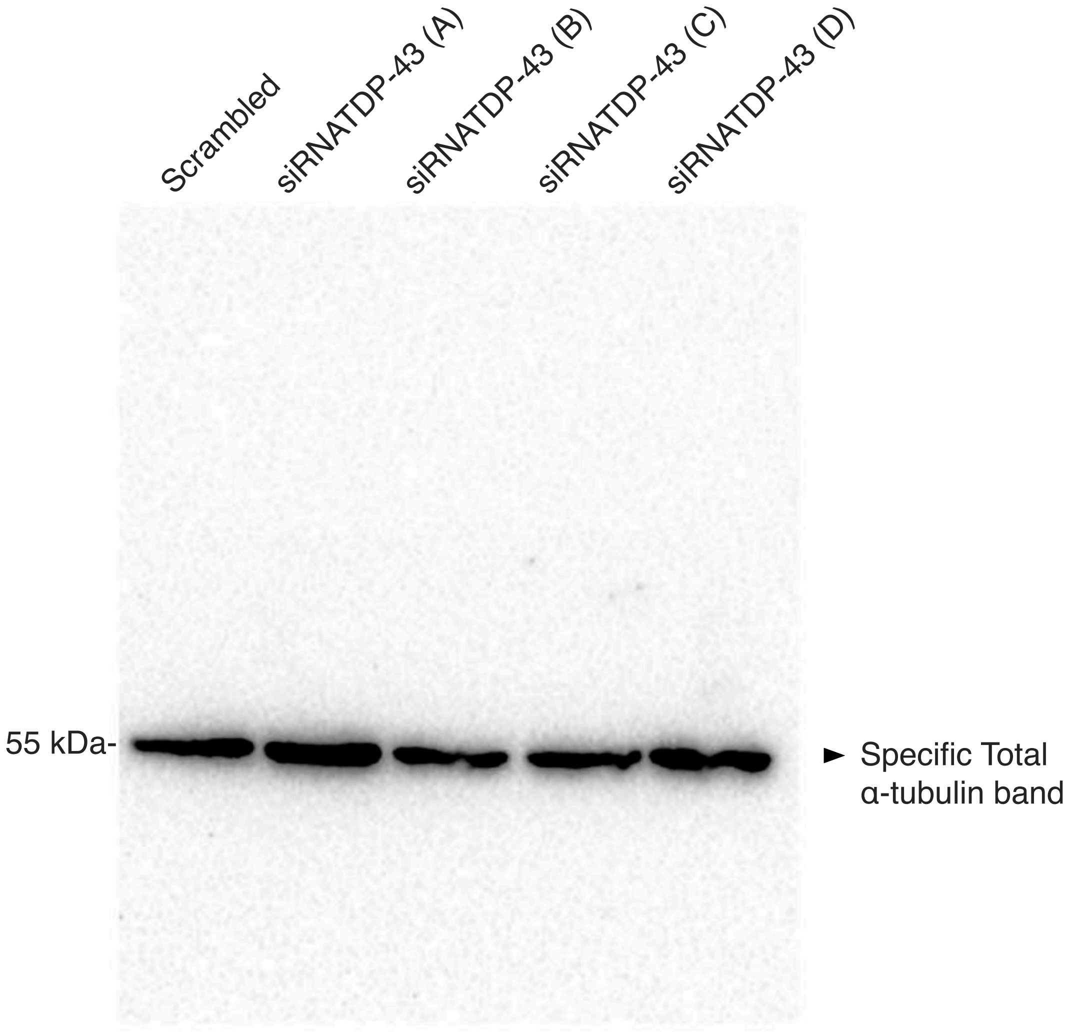

Replicate 1 as figure format

A

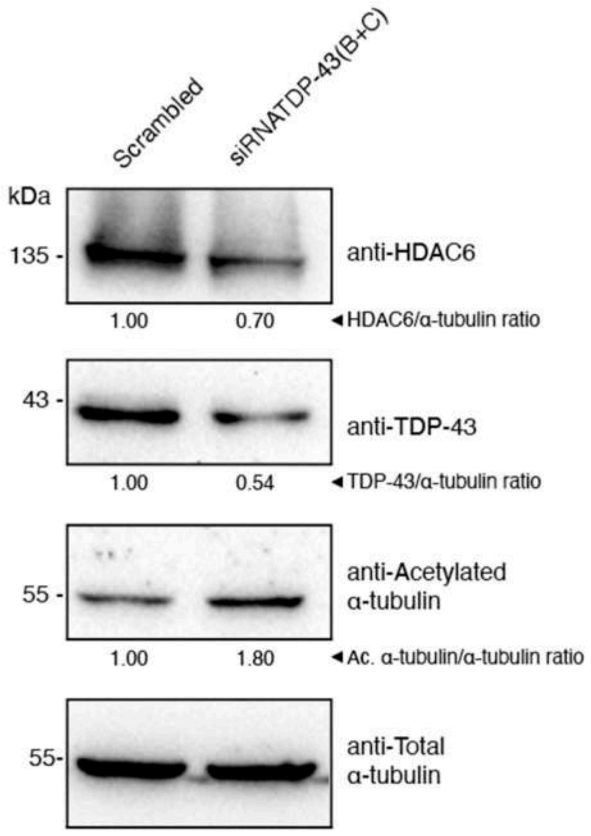

Replicate 2 as figure format

A

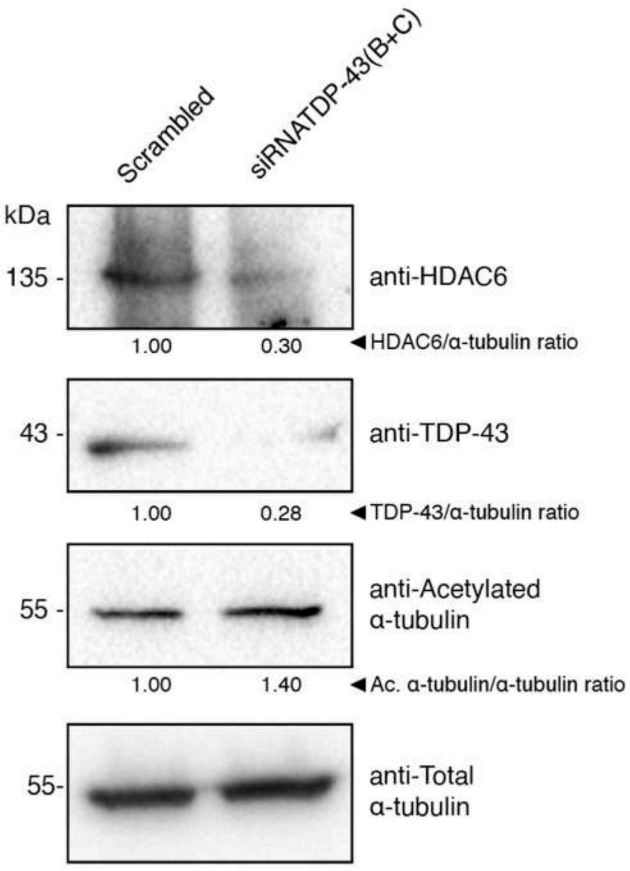

Replicate 3 as figure format

A

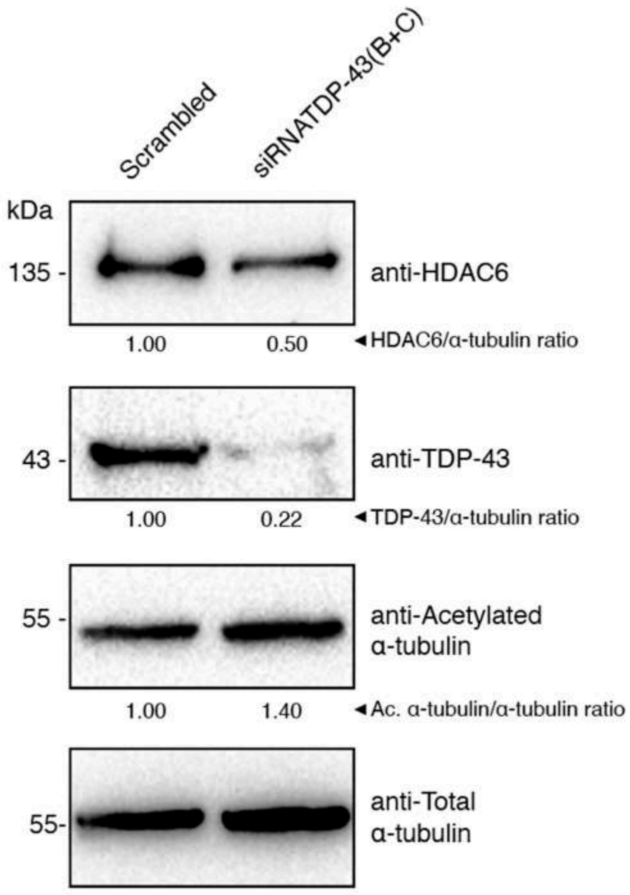

Figure S4. Replicate 1 HDAC6 complete gel Western-blot associated with Figure 6A
Cabrera-Rodríguez, R., *et al.*

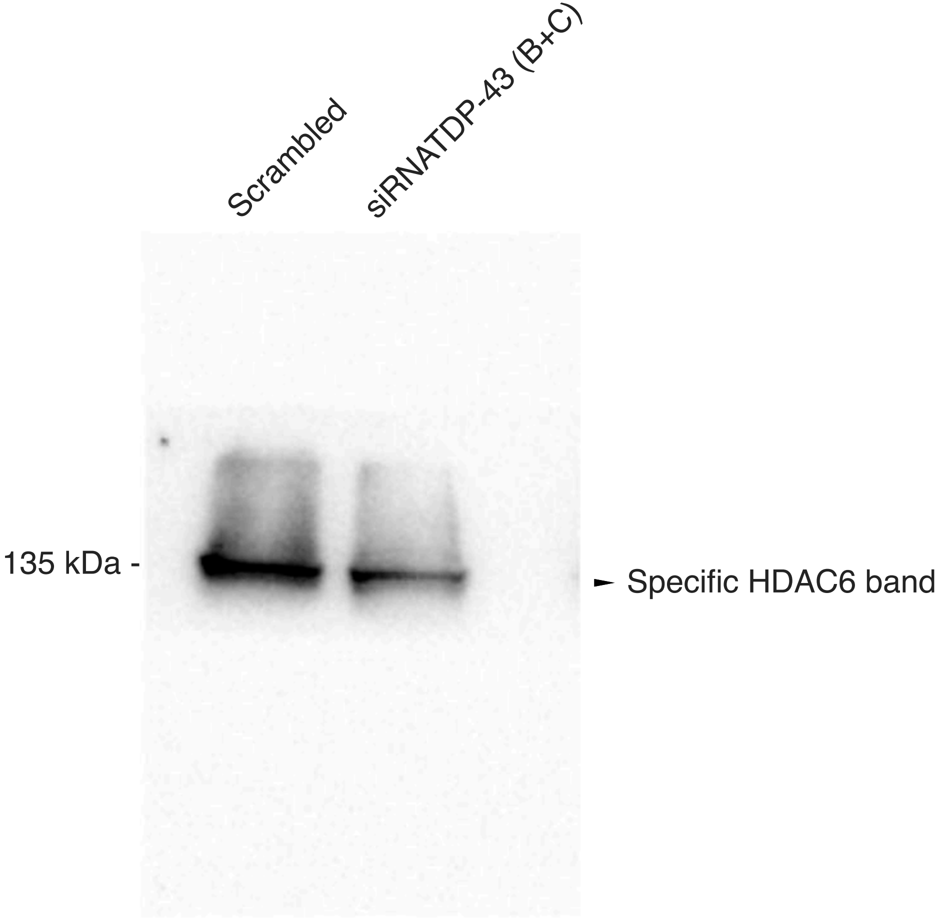

Figure S4. Replicate 1 TDP-43 complete gel   Western-blot associated with Figure 6A

Cabrera-Rodríguez, R., *et al.*

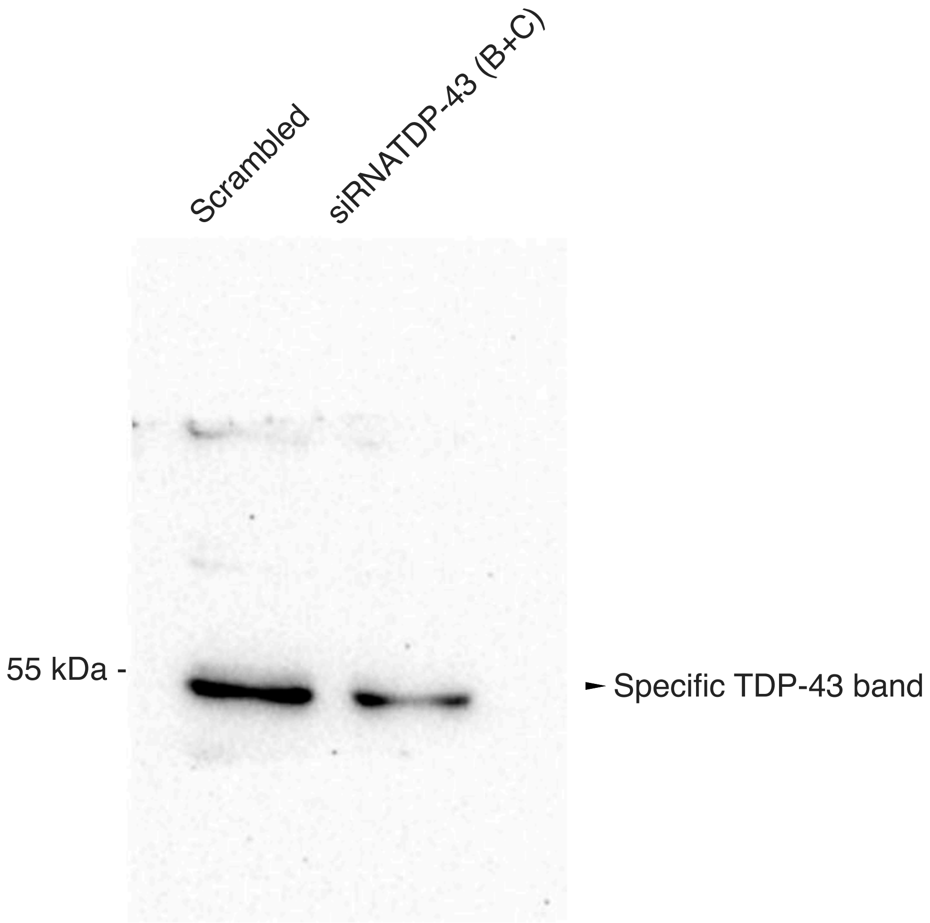

Figure S4. Replicate 1 Acetylated α-tubulin complete gel   Western-blot associated with Figure 6A
Cabrera-Rodríguez, R., *et al.*

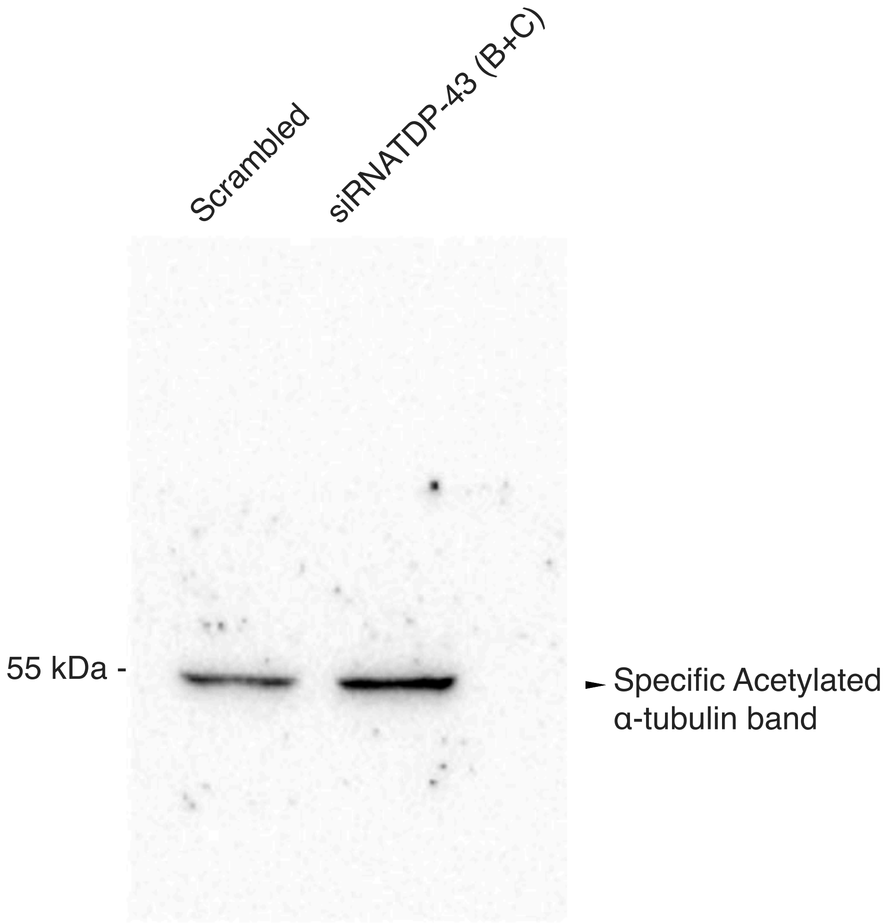

Figure S4. Replicate 1 Total α-tubulin complete gel Western-blot associated with Figure 6A

Cabrera-Rodríguez, R., *et al.*

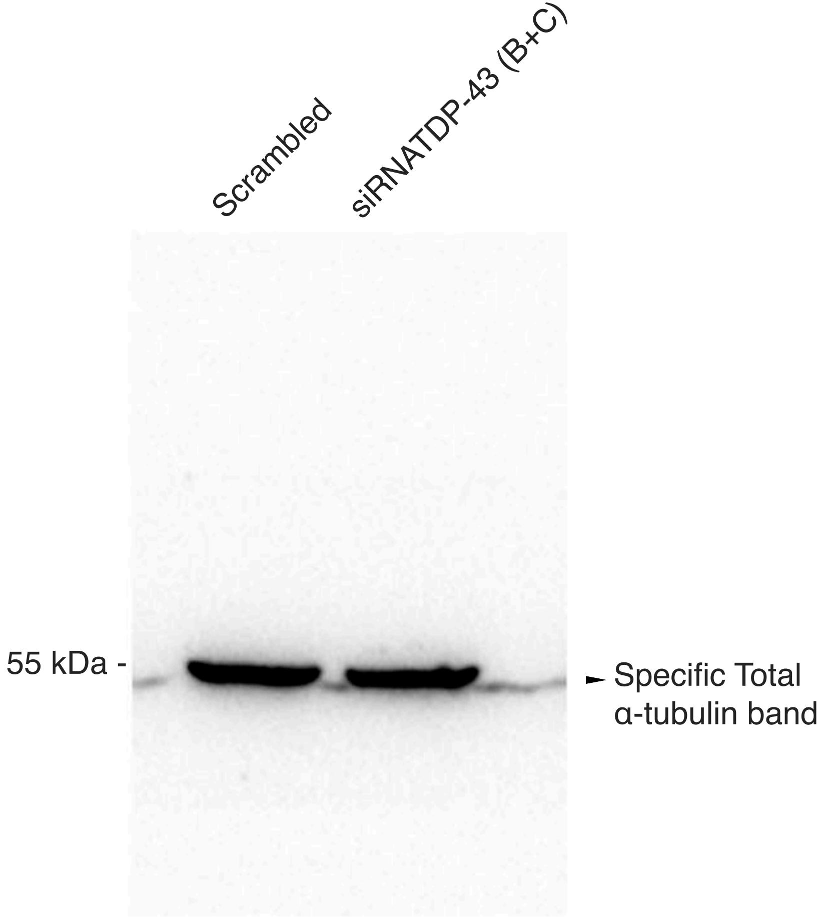

Figure S4. Replicate 2 HDAC6 complete gel Western-blot associated with Figure 6A
Cabrera-Rodríguez, R., *et al.*

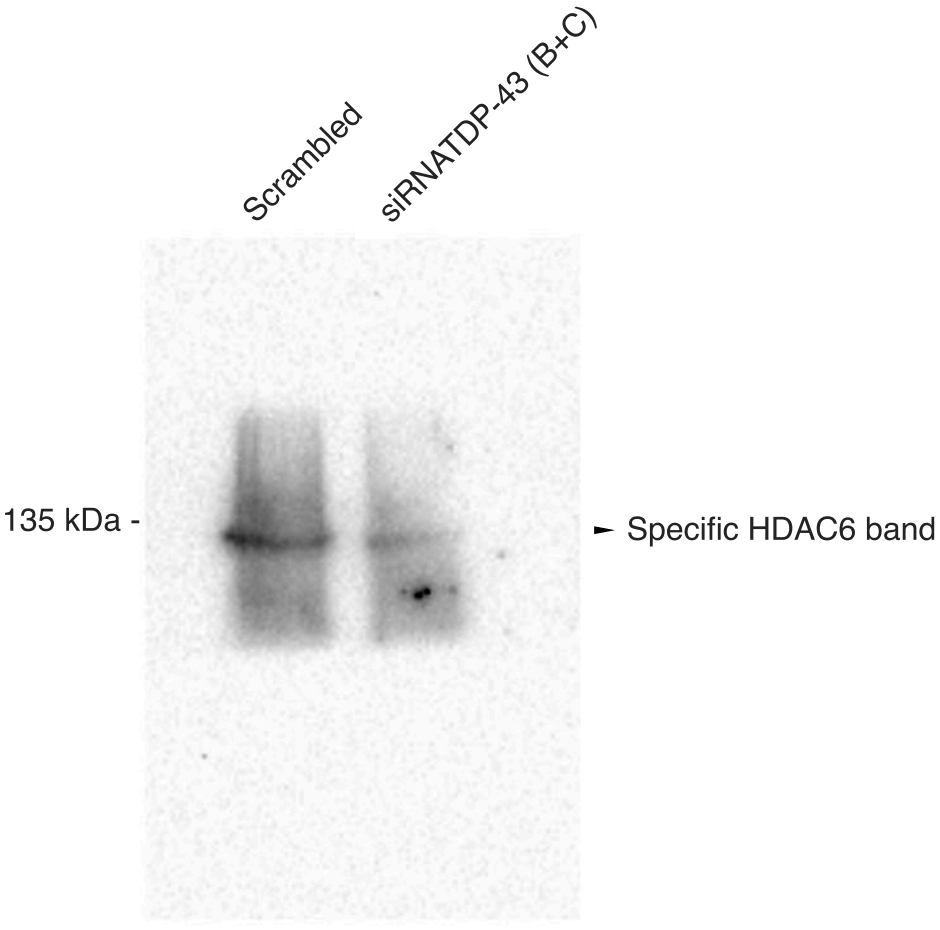

Figure S4. Replicate 2 TDP-43 complete gel   Western-blot associated with Figure 6A

Cabrera-Rodríguez, R., *et al.*

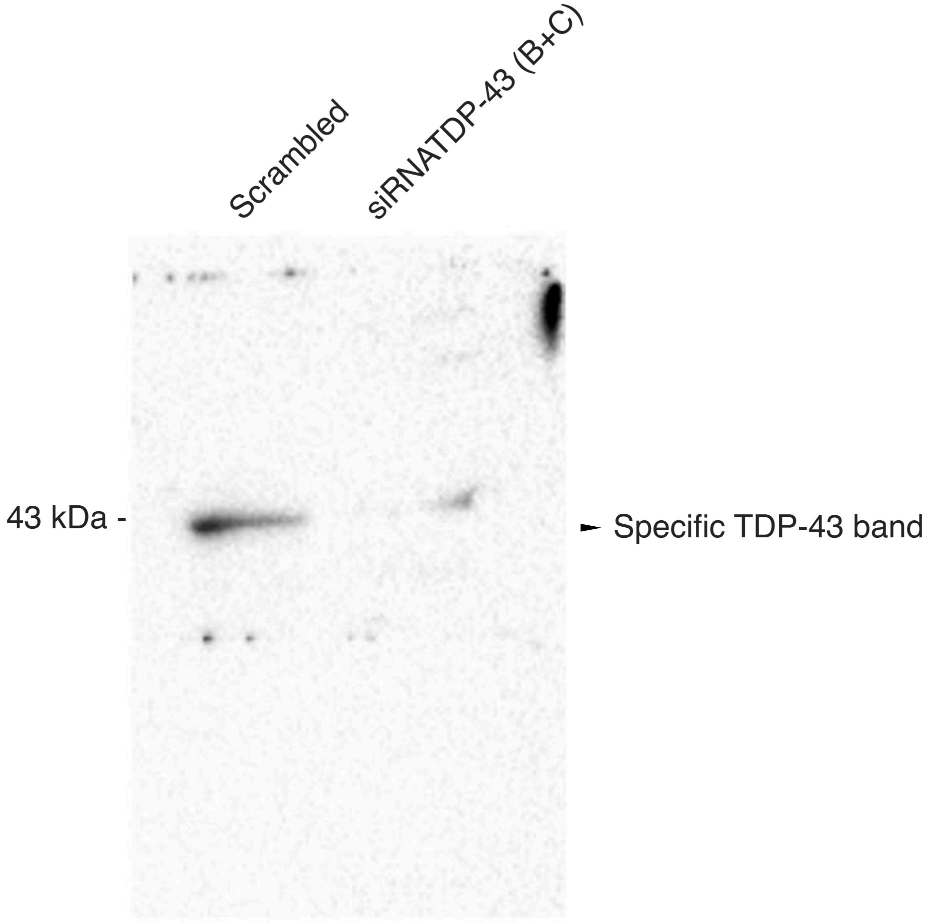

Figure S4. Replicate 2 Acetylated α-tubulin complete gel   Western-blot associated with Figure 6A
Cabrera-Rodríguez, R., *et al.*

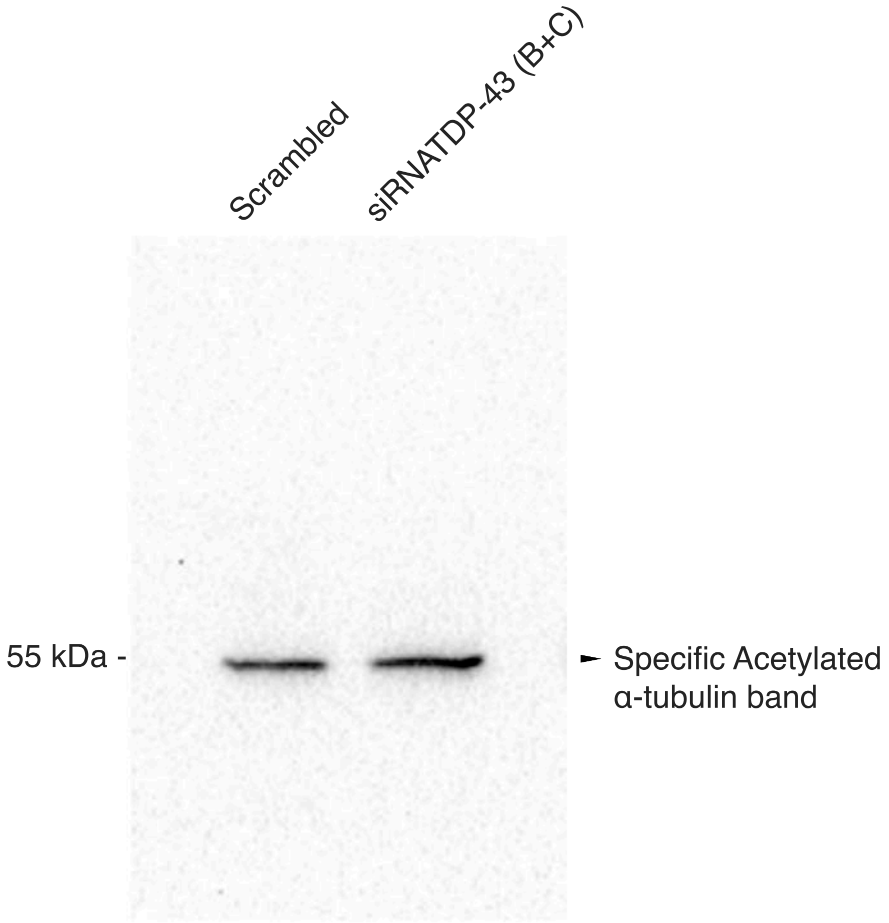

Figure S4. Replicate 2 Total α-tubulin complete gel Western-blot associated with Figure 6A

Cabrera-Rodríguez, R., *et al.*

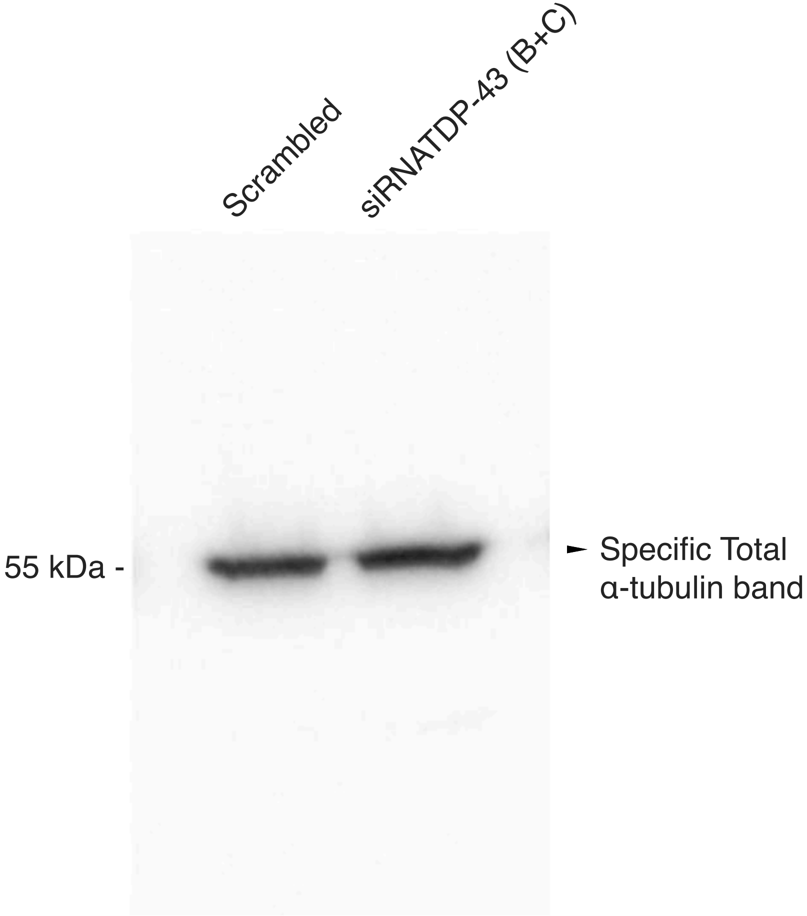

Figure S4. Replicate 3 HDAC6 complete gel Western-blot associated with Figure 6A
Cabrera-Rodríguez, R., *et al.*

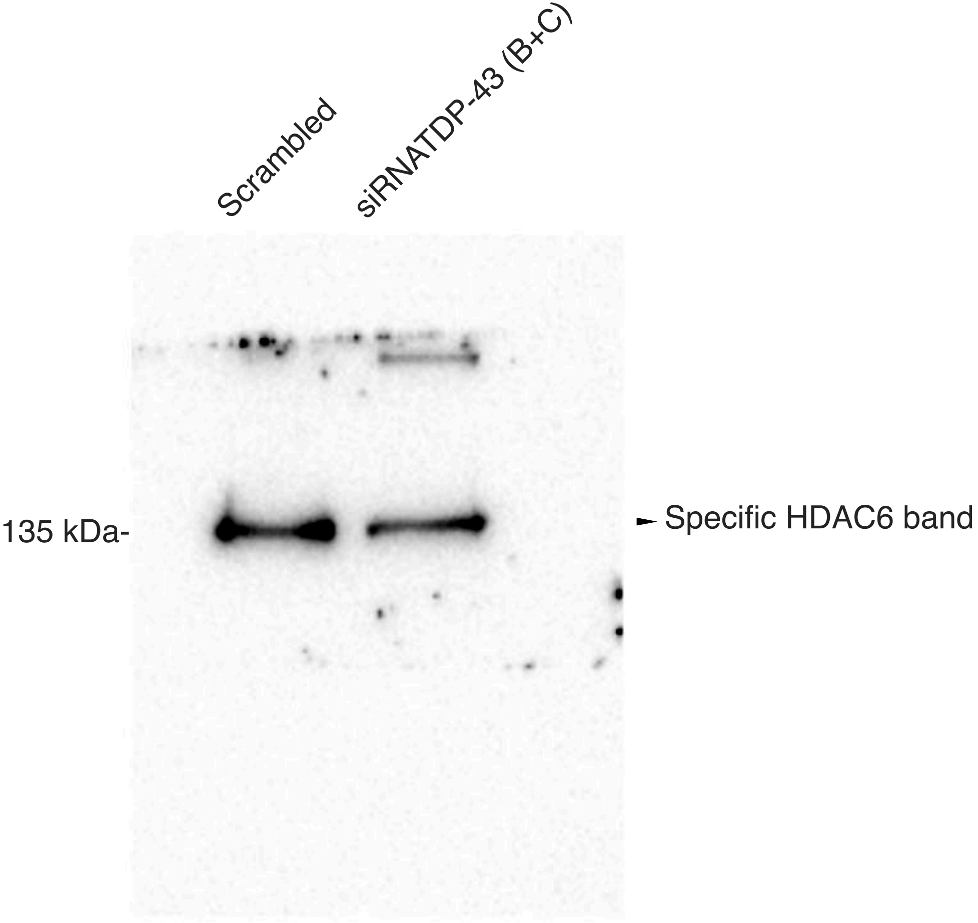

Figure S4. Replicate 3 TDP-43 complete gel Western-blot associated with Figure 6A
Cabrera-Rodríguez, R., *et al.*

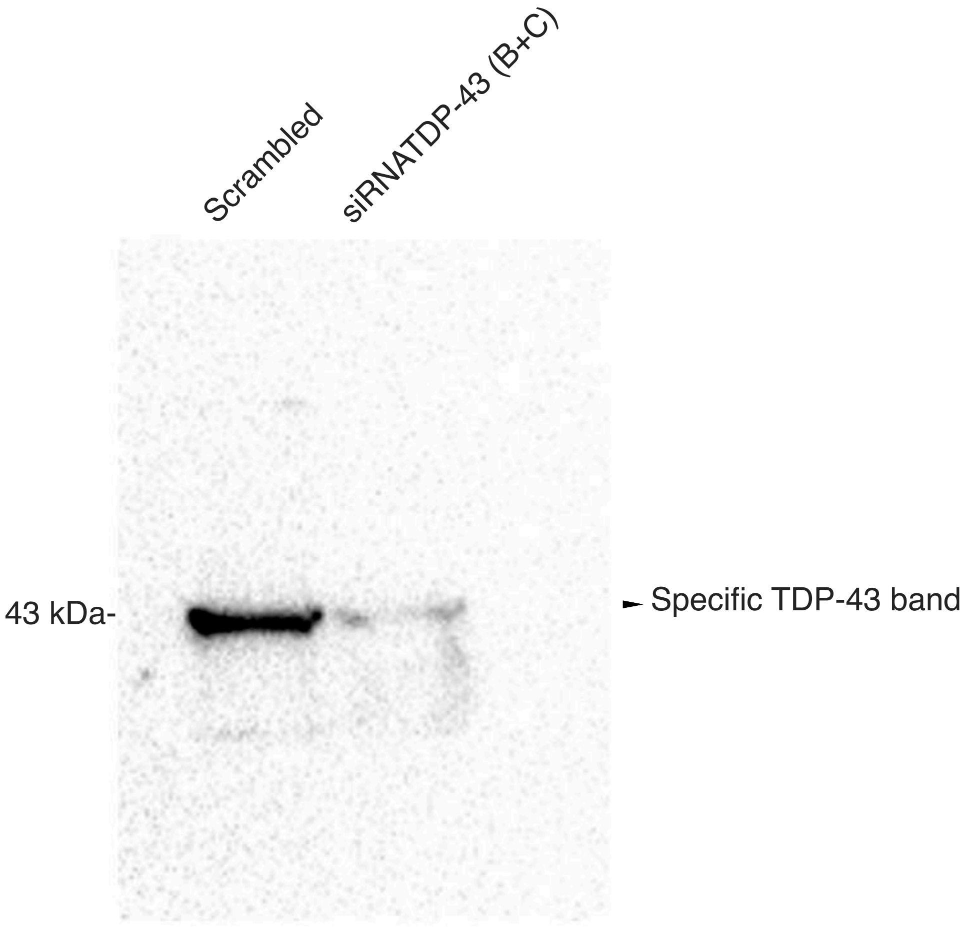

Figure S4. Replicate 3 Acetylated α-tubulin complete gel Western-blot associated with Figure 6A
Cabrera-Rodríguez, R., *et al.*

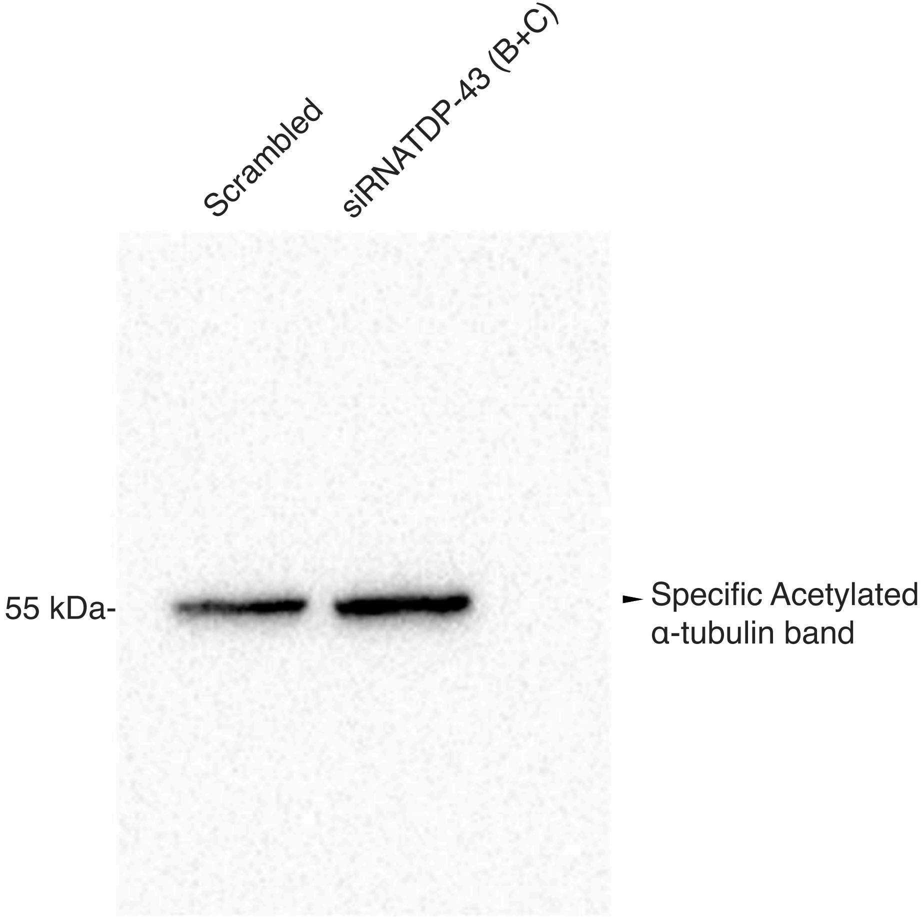

Figure S4. Replicate 3 Total α-tubulin complete gel Western-blot associated with Figure 6A
Cabrera-Rodríguez, R., *et al.*

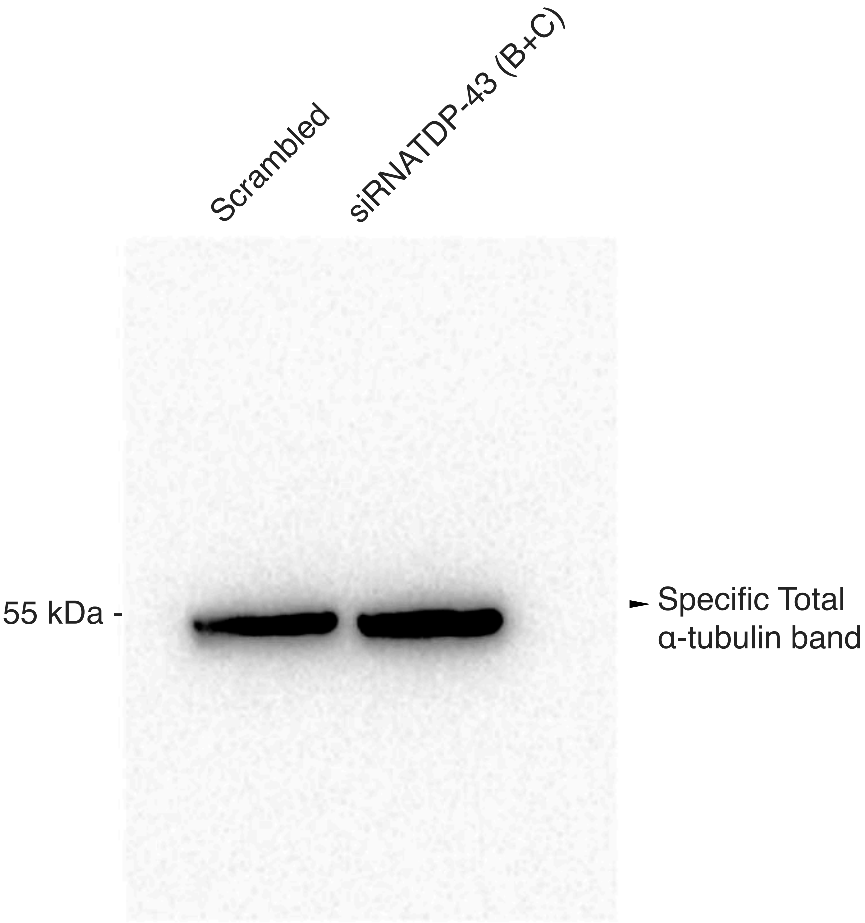

Supplement: Supplementary file 1 [file ijms-23-06180-s001.zip › ijms-1736305-supplementary.pdf]
